# Detection of Chimeric Cellular: HIV mRNAs Generated Through Aberrant Splicing in HIV-1 Latently Infected Resting CD4+ T Cells

**DOI:** 10.3389/fcimb.2022.855290

**Published:** 2022-04-28

**Authors:** Michelle Y-H Lee, Georges Khoury, Moshe Olshansky, Secondo Sonza, Glen P. Carter, James McMahon, Timothy P. Stinear, Stephen J. Turner, Sharon R. Lewin, Damian F. J. Purcell

**Affiliations:** ^1^ Department of Microbiology and Immunology, The University of Melbourne at the Peter Doherty Institute for Infection and Immunity, Melbourne, VIC, Australia; ^2^ Department of Microbiology, Biomedical Discovery Institute, Monash University, Melbourne, VIC, Australia; ^3^ Doherty Applied Microbial Genomics, The University of Melbourne at the Peter Doherty Institute for Infection and Immunity, Melbourne, VIC, Australia; ^4^ Department of Infectious Diseases, Monash University and Alfred Hospital, Melbourne, VIC, Australia; ^5^ Department of Infectious Diseases, The University of Melbourne at the Peter Doherty Institute for Infection and Immunity, Melbourne, VIC, Australia; ^6^ Victorian Infectious Diseases Service, The University of Melbourne at the Peter Doherty Institute for Infection and Immunity, Melbourne, VIC, Australia

**Keywords:** HIV latency, readthrough transcription, chimeric mRNAs, Tat trans-activator, proviral DNA integration, integration site analysis

## Abstract

Latent HIV-1 provirus in infected individuals on suppressive therapy does not always remain transcriptionally silent. Both HIV-1 LTR and human gene promoter derived transcriptional events can contribute HIV-1 sequences to the mRNA produced in the cell. In addition, chimeric cellular:HIV mRNA can arise through readthrough transcription and aberrant splicing. Using target enrichment coupled to the Illumina Mi-Seq and PacBio RS II platforms, we show that 3’ LTR activation is frequent in latently infected cells from both the CCL19-induced primary cell model of HIV-1 latency as well as *ex vivo* samples. In both systems of latent HIV-1 infection, we detected several chimeric species that were generated *via* activation of a cryptic splice donor site in the 5’ LTR of HIV-1. Aberrant splicing involving the major HIV-1 splice donor sites, SD1 and SD4 disrupts post-transcriptional processing of the gene in which HIV-1 is integrated. In the primary cell model of HIV-1 latency, Tat-encoding sequences are incorporated into the chimeric mRNA transcripts through the use of SD4. Our study unravels clues to the characteristics of HIV-1 integrants that promote formation of chimeric cellular:HIV mRNA and improves the understanding of the HIV-1 RNA footprint in latently infected cells.

## Introduction

Knowledge of the HIV-1 RNA footprint in cells with latent provirus is currently incomplete. In most individuals on suppressive therapy, cell-associated (CA) HIV-1 RNA can be consistently detected throughout infection at levels close to the limit of detection (Fischer et al., 2002). Very low copy numbers (<10 copies/million cells) of both unspliced (US RNA) and multiply spliced (MS RNA) HIV-1 mRNA have been detected in resting memory CD4+ (rCD4+) T cells isolated from virally suppressed individuals (Hermankova et al., 2003). Short, abortive HIV-1 LTR-derived transcripts no longer than 181 nucleotides were found to be abundant in the rCD4+ T cells from the same study.

At any given time in a latently infected cell, there are multiple layers of repression at the latent HIV-1 promoter (Khoury et al., 2018a). However, stochastic reactivation of HIV-1 transcription can result from fluctuations in the levels of cellular factors involved in reinforcement of silent infection (Burnett et al., 2009). These transcriptional events likely result in production of the aforementioned abortive transcripts and would not lead to protein production. Occasionally, however, this could facilitate the process of intermittent reactivation (IR) of proviruses, which manifest as the viral RNA blips, observed in individuals who have been on uninterrupted treatment (Ramratnam et al., 2000). The exact mechanisms driving IR have not been determined but encounter of the rCD4+ T cell with its antigen or activation stimuli are thought to be involved (Chun et al., 1998; Rong and Perelson, 2009). IR of latent proviral transcription is a source of authentic viral promoter derived HIV-1 RNA in a latently infected cell. Other pathways of HIV-1 RNA accumulation in a latently infected cell may involve transcription initiated at a gene promoter.

As an artefact arising from the propensity of HIV-1 to integrate into introns of actively transcribed genes (Han et al., 2004), readthrough transcripts containing both cellular and HIV-1 sequence represent an additional HIV-1 RNA footprint in latently infected cells. These transcripts could potentially be used for the expression of HIV-1 gene products. RNA processing of cellular:HIV readthrough transcripts by the cellular splicing machinery removes the HIV-1 sequences for degradation. Alternatively, it may create chimeric mRNAs that do not present junctions at the integration sites of HIV-1 (Cesana et al., 2017; Pinzone et al., 2019). Mature transcripts that contain specifically an exon or both exons encoding Tat will produce functional protein, which could go on to reactivate the latently infected cell. Inducing the expression of Tat in latently infected cells will overcome blocks at the elongation step, mediated by its role as an adaptor for the recruitment of P-TEFb to the HIV-1 LTR (Mancebo et al., 1997; Zhu et al., 1997). Although Tat cannot directly initiate transcription, it has been shown that addition of Tat to latently infected T-cell lines, primary models of latent infection (*in vitro*) and CD4+ T cells derived from HIV infected individuals on cART (*ex vivo*) activates viral expression outside the context of T cell activation (Lin et al., 2003; Lassen et al., 2006; Razooky et al., 2015; Khoury et al., 2018b; Khoury et al., 2019). This suggests that the Tat positive feedback circuitry alone is sufficient for overcoming the early steps of transcriptional repression and is also more effective than cellular activation for inducing transcription from silenced full-length proviruses (Razooky et al., 2015). In addition, the role of Tat in HIV-1 replication extends beyond the transactivation of transcription, hence inducing expression of Tat in a latently infected cell would exert beneficial effects throughout the pathways encompassed within the domain of mRNA biogenesis. These include interactions with several histone-modifying or nucleosome remodelling complexes such as p300/CBP, PCAF and SWI/SNF, activation of NFκB for initiation of transcription and influence of splicing through association with p32 and ASF/SF-2 (Benkirane et al., 1998; Hottiger and Nabel, 1998; Marzio et al., 1998; Demarchi et al., 1999; Lusic et al., 2003; Agbottah et al., 2006; Berro et al., 2006; Mahmoudi et al., 2006). Considering the evidence presented above, further exploration of this pathway of Tat expression in latently infected cells is warranted. Use of induction of Tat expression for reactivation of the HIV-1 LTR would be a more potent and specific mechanism for reversing HIV-1 latency.

Previous studies have briefly reported on readthrough transcripts and mostly described these as containing sequences of the 5’ LTR which do not usually appear in authentic HIV-1 LTR derived mRNA (Han et al., 2008; Bullen et al., 2014; Elliott et al., 2014; Sherrill-Mix et al., 2015; Pasternak et al., 2016). The study by Sherrill-Mix et al.examined cellular:HIV chimeric reads from primary cells productively infected with HIV_89.6_ by Illumina HiSeq 2000 and described chimeras with junctions elsewhere in the HIV-1 genome, including the major HIV-1 splice donor site, SD4 (Sherrill-Mix et al., 2015). Further studies characterised the nature of aberrant splicing and investigated chimeric cellular:HIV mRNA in the context of HIV-1 integration into transcriptionally active cellular genes and its impact on persistence of HIV-1 infected clones in infected individuals on suppressive therapy (Cesana et al., 2017; Liu et al., 2020). A recent study detected chimeric cellular:HIV mRNAs from HIV-1-infected individuals on suppressive therapy by sequencing cells isolated using HIV-1 RNA specific probes during early latency reversal (Liu et al., 2020). They showed disruption of host gene transcription driven by HIV-1-to-host aberrant splicing. The HIV-1 protein coding potential of the chimeras in these studies were not explored.

Here, we interrogate the existence of cellular:*tat* chimeric mRNAs in the CCL19-induced primary cell model of HIV-1 latency by short read sequencing on the Illumina Mi-Seq platform, whilst the long read PacBio RS II platform is used for sequencing of samples from virally suppressed infected individuals. Both approaches are coupled with a target enrichment step, where custom-made probe libraries contain pools of 50-120 bp primers that cover the entire HIV-1 genome. In this study, we identify Tat exon 2 containing chimeras in latently infected primary cells but not in samples from infected individuals on combination antiretroviral therapy (cART). These chimeras are generated through the use of HIV-1 SD4 in conjunction with either canonical or cryptic splice acceptors in the human genome. In addition, a frequently activated cryptic splice donor site was observed in chimeras derived from both the primary cell model as well as patient samples. Overall, our results reveal production of HIV-1 proteins is not favoured from chimeric transcripts and thus readthrough transcription is unlikely to perturb HIV-1 latency.

## Results

### A Chimeric PPP5C:5’LTR/*tat* mRNA Is Detected in J-Lat6.3 Cells

**Figure 1:**
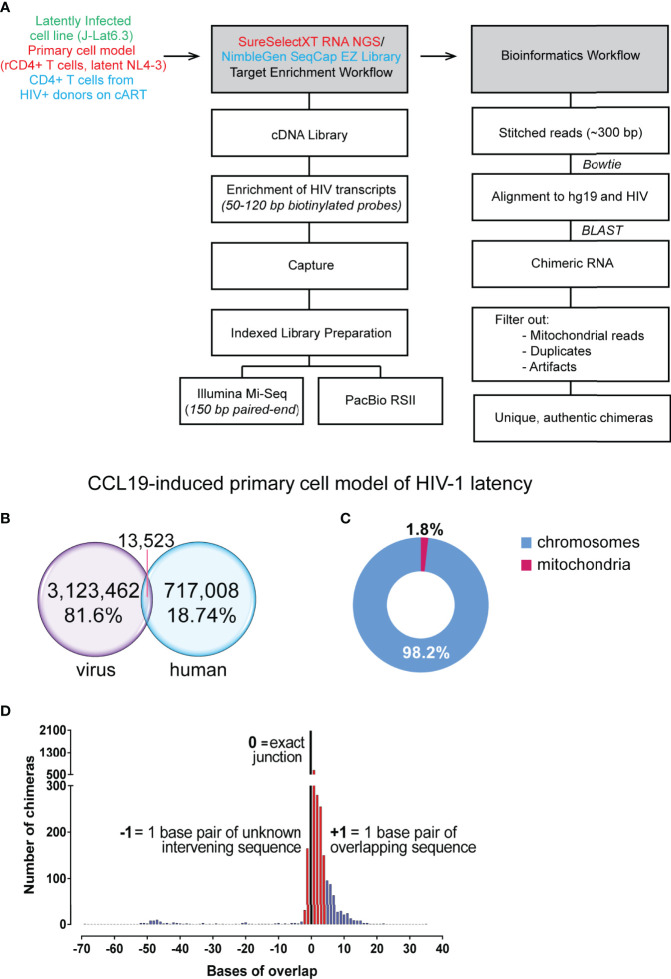
Summary of reads from primary cell model of HIV-1 latency. **(A)** Workflow followed for conversion of RNA to cDNA, target enrichment and sequencing on the Illumina Mi-Seq or PacBio RSII platforms and bioinformatics pipeline utilised for detection of chimeric reads. **(B)** Target enrichment through the Agilent SureSelect platform was of high efficiency with 81.6% of reads on target for HIV-1 sequences. **(C)** 1.8% of chimeric reads had a junction associated with mitochondrial RNA, a reflection of a fraction of the background levels of PCR-mediated recombination events. **(D)** Filtered chimeric reads had exact junctions between the human and virus sequences (black bar), contained less than 3 base pairs of unknown intervening or contained less than 5 base pairs of overlapping sequence (red bars).

To assess the suitability of the chosen target enrichment and Illumina sequencing methodology for detection of chimeric cellular:HIV mRNA, we used whole cell RNA extracted from the latently infected J-Lat6.3 cell line as bait. In this T cell line, the single integration site of HIV-1 has been annotated to intron 4 of the PPP5C gene (Jordan et al., 2003). Poly(A) RNA was purified, followed by fragmentation, reverse transcription and finally hybridisation with a customized NL4-3 specific biotinylated RNA probe library (pool of 438 distinct probes that cover the entire HIV genome) for target capture of HIV-1 sequences. The HIV-1-sequence-enriched cDNA library was then indexed and sequenced on the Illumina Mi-Seq platform, generating paired-end reads of 150 base pairs (bp) (**Figure 1A**; **Supplementary Figure 1**). *In silico*, where possible, paired-end reads were stitched together by use of at least 10 base pairs of overlap. Both stitched and non-stitched pairs were aligned to the NL4-3 HIV-1 genome and the human genome (hg19 build) for detection of chimeric reads (**Figure 1A**; **Supplementary Figure 1**). Paired-end reads are referred to as single reads from this point forward. Out of the 217,743 reads, 435 cellular:HIV chimeras were detected in the J-Lat6.3 sample, representing 0.2% of reads (**Supplementary Figure 2A**). Ten of these were not associated with the PPP5C gene and is reflective of the background recombination rates (2.3%) induced by PCR in the process of cDNA library generation. The junction between human and HIV-1 sequences in all of the PPP5C:HIV chimeras was at the 5’ integration site of HIV-1 in intron 4 of PPP5C. Splicing between a canonical cellular splice donor (SD) at the 3’ boundary of exon 4 of PPP5C and a cryptic splice acceptor (SA) site upstream of the HIV-1 5’ LTR was the predominant species of chimeric mRNA observed (**Supplementary Figure 2B**, top - chimeric mRNA species #1). Similar PPP5C:HIV chimera were detected from untreated J-Lat6.3 cells by Sanger Sequencing (data not shown). A single chimeric PPP5C:5’LTR/*tat* transcript was isolated from J-Lat6.3 using a forward primer complementary to PPP5C exon 3 and a reverse primer binding to the *tat* exon 2 region (**Supplementary Figure 2B**, bottom). The first encoding exon of Tat was incorporated into the chimeric mRNA through the use of canonical HIV-1 SD1 to SA3 splicing. The chimeric PPP5C:HIV mRNAs detected by Illumina contained only sequences of the 5’ LTR and thus we cannot determine if *tat* sequences were part of the original unfragmented transcripts. Additional chimeric transcripts isolated did not present any splicing within the span of the read but showed varying lengths of the PPP5C intron 4 sequence in the lead up to the 5’ LTR of HIV-1, starting upstream of the cryptic splice acceptor site (**Supplementary Figure 2B**, top - chimeric mRNA species #2). The successful detection of PPP5C:HIV chimeras using the J-Lat6.3 cell line model of HIV-1 latency validated the use of the enrichment strategy for detection of chimeric cellular:HIV mRNAs in more complex models of HIV-1 latency.

### Chimeric mRNA From Cell Line Models of HIV-1 Latency Can Transactivate the LTR in TZM-bl Cells

To determine whether the PPP5C:5’LTR/*tat* transcript can facilitate production of functional Tat, the chimeric fragment containing sequences of *tat* exon 2 encoding a 55-amino acid version of Tat protein was introduced into an expression vector. Derivatives of the isolated chimera were also generated, which encode the 72 amino acid version of Tat and/or contain the TAR C37T mutation based on the ACH2 cell line (Emiliani et al., 1996) and/or have the first 4 exons of the human gene PPP5C reconstituted without the intervening introns. Efficient transactivation of the LTR in TZM-bl cells is achieved by the unmodified version of the PPP5C:5’LTR/*tat* chimera (**Supplementary Figure 2C**). Introduction of the TAR C37T mutation reduces Tat transactivation of the HIV-1 LTR by 2- to 3-fold. In the PPP5C:5’LTR/*tat* system, this results in no more than a 30% reduction in production of Tat. This suggests that CMV-driven chimeric transcripts are produced initially followed by the translation of Tat from a chimeric mRNA. Thereafter, Tat is able to transactivate the 5’ LTR on the introduced construct, resulting in HIV-1 promoter derived transcripts. This contributes to an increase in the amount of functional Tat produced. In addition, the reconstitution of all the exons preceding the HIV-1 sequences in the chimera (PPP5C exons 1 to 4) did not change the level of Tat transactivation observed (6.3^P^ Tat 72 vs 6.3 Tat 72 and 6.3^P,ΔLTR^ Tat 72 vs 6.3^ΔLTR^ Tat 72, **Supplementary Figure 2C**). The PPP5C:5’ LTR/*tat* transcript isolated from J-Lat6.3 cells provides an example of functional Tat production from a chimeric cellular:HIV mRNA context.

### Authentic Chimeras Were Detected From the Primary Cell Model of HIV-1 Latency Where 52 Were Formed Through Aberrant Splicing

Cell line models only present one integration site of HIV-1 for study, and the understanding of the nature of readthrough transcripts in HIV-1 latency can be improved with use of a primary cell model. We used the CCL19 primary model of latency that have been previously described to mostly resemble rCD4+ T cells from aviraemic patients (Spina et al., 2013). HIV-1 latency in rCD4+ T cells is established through CCL19 induction where cells were subsequently infected with NL4-3 virus and left in culture for 4 to 7 days (**Supplementary Figure 3A**). The workflow for cDNA library preparation and enrichment as well as the downstream bioinformatic analysis is at that described in the previous figure (**Figure 1A**; **Supplementary Figure 1**). Two independent experiments were conducted for the detection of chimeric cellular:HIV mRNA with a total of four samples from the CCL19-induced primary cell model of HIV-1 latency sequenced (**Supplementary Figure 3B**). Only 59% of raw paired-end reads were retained in the analysis where they either aligned to HIV-1, human or both (**Supplementary Figure 3C**). The target capture procedure was highly efficient, with an overall on-target enrichment of 81.6% for the CCL19-induced latently infected primary cell samples (**Supplementary Figure 3C**). Together with 7,087 unstitched reads, 13,523 paired end reads were determined to be chimeric (**Figure 1B**). Of these initial 20,610 reads determined to be chimeric by Bowtie 2, only 7,632 were found to align to the human genome with high confidence by BLASTn (**Supplementary Figure 3D**). After elimination of contaminating plasmid sequences and mitochondrial RNA associated chimera, 7,494 reads across both experiments were determined to be chimeric cellular:HIV reads (0.24% of reads aligning to HIV-1, **Supplementary Figure 3D**). Whilst there have been no reports of HIV-1 integration sites in mitochondrial DNA (Wang et al., 2007), 1.8% of the pre-filter chimeras in the primary cell model dataset were associated with mitochondrial RNA (**Figure 1C**). This likely reflects the background recombination events that are occurring during PCR amplification (Zagordi et al., 2010; Di Giallonardo et al., 2013). Previous studies have shown that HIV-1 RNA can accumulate in the mitochondria (Somasundaran et al., 1994), potentially indicating that the proximity of HIV-1 RNA to mitochondrial RNA promotes the formation of these PCR recombinants. These chimeras formed between mitochondrial and HIV-1 sequences were also abundant in the previous study of chimeric mRNAs by Sherrill-Mix et al. (2015). The chimeras were then filtered on the basis of their junction sites between human and HIV-1 segments and a total of 4,123 unique chimeric cellular:HIV mRNAs were detected in this dataset (**Supplementary Figure 3D**). Finally, manual examination of all 4,123 unfiltered chimeras eliminated evident artefacts, mainly formed through recombination or associated with ribosomal RNA (rRNA) or pseudogenes without annotated RNA transcripts, leaving 3,599 chimeras that were potentially authentic (**Supplementary Figure 3D**). This accounts for only 0.12% of reads that aligned to the HIV-1 genome. More than half (58.4%, 2081/3599) of these presented with exact junctions. The 3,599 filtered chimeras all possessed an overlap between the human and HIV sequences of no more than 4 base pairs (bp) or unmapped intervening sequences not more than 2 bp in length (**Figure 1D**). These thresholds were arbitrarily chosen since these accounted for the vast majorities of the chimeras detected and would constitute a proportion much higher than the 2.3% of PCR recombinants detected in the J-Lat6.3 dataset. A biological mechanism for the formation of these species, given that they are not artefacts formed from reverse transcriptase template skipping (Cocquet et al., 2006), has not been determined. Stringent manual examination of the filtered reads resulted in annotation of 206 chimeras that were formed through known biological mechanisms (**Supplementary Figure 3D**): 154 directly show the integration sites of HIV-1 and are examples of the classical readthrough transcripts reported in the past, while the remaining 52 were formed through aberrant splicing, linking human and HIV-1 splice sites to create chimeric cellular:HIV mRNA.

### Integration of HIV-1 Into Introns and in the Same Orientation as Human Genes Give Rise to Readthrough Transcripts

**Figure 2:**
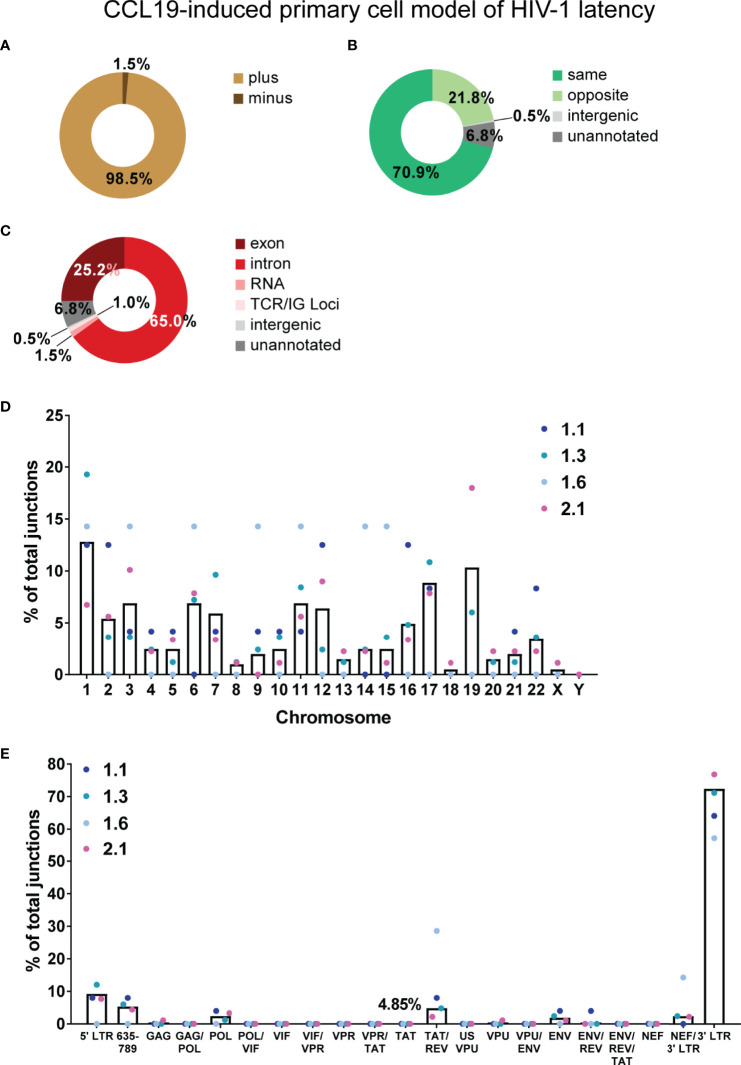
Overview of chimeric mRNAs detected in the primary cell model of HIV-1 latency. Summary of the genetic features of the authentic chimeric reads: HIV sense **(A)**, orientation of HIV integration relative to the chimera-associated human gene **(B)** and region of the human gene associated with the junctions in the chimeras **(C)**. The percentage of junctions represented by each chromosome for the four latent samples is shown by the coloured dots and mean of the four samples is shown by the black bar **(D)**. **(E)** The percentage of total junctions for each HIV-1 region associated with the 206 authentic chimeras. Several open reading frames (ORFs) of genes in the HIV-1 genome overlap, hence the genome of NL4-3 was divided into discrete regions, each described by all the associated genes to which the sequence belongs. Tat exon 2 is represented by 3 discrete regions (region overlapping with VPR - VPR/TAT, region with tat alone - TAT and region overlapping with rev exon 2 - TAT/REV), and in this analysis is taken from SA3 through to SD4 (nt 5777 to 6044). Similarly, Vpr is also represented by 3 discrete regions (region overlapping with Vif - VIF/VPR, region with Vpr alone - VPR and region overlapping with tat Exon 2 - VPR/TAT). There is an overlap in the filtered reads between the percentages of chimeras counted for Tat exon 2 and Vpr as they have a region in common. The percentage of junctions represented by each HIV-1 region for each of the four samples is shown as coloured dots and mean of the four samples is shown by the black bar. Percentages of chimeras are shown for the two regions of interest, Tat exon 2 and Vpr (filtered reads) and the TAT/REV region (authentic). ENV/REV/TAT refers to exons 3 of Rev and Tat.

The target enrichment workflow used in this study retained strand information, where three pieces of information (HIV-1 strand, human strand and location of human gene) allowed rapid determination of the orientation of HIV-1 integration relative to the associated human gene. Genetic features of the authentic chimeras are summarized in **Figure 2**. Generation of a cDNA library was completed prior to capture of HIV-1 specific sequence, meaning that any RNA transcript with an intact poly(A) tail could be captured in the target enrichment workflow once converted to cDNA regardless of whether it was sense (resemble plus strand) or antisense (resemble minus strand) for HIV-1. Only 1.5% of chimeras were detected with antisense sequences for HIV-1 (**Figure 2A**), suggesting a much lower frequency of transcription of these species of mRNA compared to sense HIV-1 RNA. The majority of the human sequences in the chimeras were sense (5’ → 3’) for the associated gene, meaning that the orientation of HIV-1 was more frequently in the same direction as the human gene (70.9% vs 21.8%, **Figure 2B**) than in the opposite. This is in agreement with previous studies describing readthrough transcription as a consequence of HIV-1 integration in the parallel orientation within a transcriptionally active gene (Han et al., 2008; Lenasi et al., 2008). Contrary to past reports, however, integration in the convergent orientation can also lead to readthrough transcription in some cases and was associated with 21.8% of the chimeras in this dataset. Integration within the human gene showed a preference for introns, where 65% of the chimeras were associated with the intronic region of the human gene compared to 25.2% with exonic regions (**Figure 2C**).

In the 206 authentic chimeras, junctions were observed with all human autosomal chromosomes and the X chromosome (**Figure 2D**; **Supplementary Table 1**). Chromosome 1 (12.8%) and chromosome 19 (10.3%) were the most frequently annotated (**Figure 2D**). There is a positive correlation between the percentage of integrations and number of genes associated with each chromosome (data not shown). This agrees with past observations that HIV-1 integration into the human genome is relatively random at the general chromosomal level (Schroder et al., 2002). Regions of the human genome that were associated with a specific chromosome, but not a specific gene were also detected: the human sequence in 1 chimera (0.49%) was mapped to an intergenic region, whilst 14 (6.8%) aligned to unannotated regions of the genome (**Figure 2C**).

To determine the frequency of each region of the HIV-1 genome in the chimeric reads, the NL4-3 genome was broken down into discrete segments to account for the overlapping nucleotides between different genes. Each of these regions were then described by all the genes to which those nucleotide sequences belonged. There was a bias towards the 3’ end of genome (3’ LTR) where most of the junctions were detected (**Figure 2E**; **Supplementary Table 2**). Tat exon 2, the primary region of interest for this study, is taken as nucleotides 5777 to 6044 in the NL4-3 genome (including the 53 nucleotides downstream of SA3 and upstream of the Tat AUG). This was broken up into three discrete segments – the region overlapping with *vpr* (VPR/TAT), the region with *tat* exon 2 sequence alone (TAT) and the region overlapping with *rev* exon 2 (TAT/REV). For *tat* exon 2 associated authentic chimeras, junctions were only detected in the TAT/REV region (10, **Supplementary Table 3**; 4.85%, **Figure 2E**).

Taken together these results suggest that there are specific orientations of integration of HIV-1 relative to the human gene that would promote readthrough transcription. In addition, these permissive configurations would likely only be present as part of a subset of the latently infected HIV-1 reservoir.

### 3’ LTR Transcriptional Start Site Activation Has a Big Contribution to the Pool of Chimeric Cellular: HIV mRNA in Latently Infected Cells

To understand the nature of transcription giving rise to chimeric mRNA transcripts, we further classified the chimeras by the order in which HIV-1 (virus) or human sequence appears in the read – these are described as vh or hv (v = virus, h = human); vh = virus at the 5’ end of the read and human at the 3’ end, hv = human at the 5’ end of the read and virus at the 3’ end. In terms of aberrant splicing, vh chimeras usually arise from the use of an HIV-1 splice donor and a human gene splice acceptor. Likewise, use of a human gene splice donor and an HIV-1 splice acceptor will usually give rise to hv chimeras. The vh and hv nomenclature describes the direction of the readthrough event or combination of splice elements used in the splicing event and should not be confused with the sense of the chimeras.

Of the 206 authentic chimeras detected, there were 196 in the vh configuration, where their junctions were mapped to 18 single nucleotides within the HIV-1 genome (**Supplementary Figure 4**). Two of these junctions represent the 5’ and 3’ integration sites (nucleotide (nt) 1 and 9709), four represent canonical HIV-1 splice sites (nt 726, 743, 4721 and 6044) whilst the remainder are previously uncharacterised cryptic HIV-1 splice donors (**Supplementary Figure 5**). Six junctions were annotated for the 6 authentic chimeras in the hv configuration representing the integration sites, two canonical splice acceptors (nt 9142 and 9162) and two cryptic splice acceptors (**Supplementary Figures 4** and **5B**).

**Figure 3:**
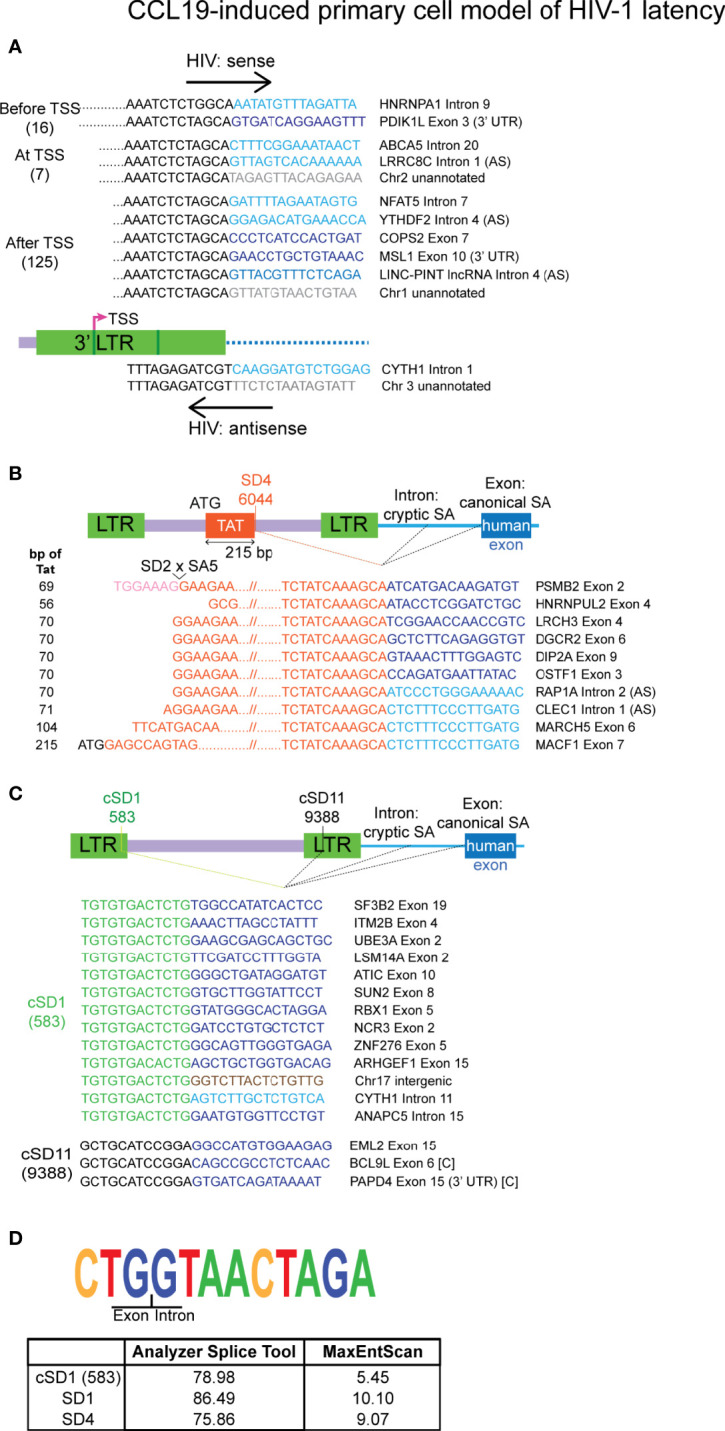
Authentic chimeras from the CCL19-induced primary cell model of HIV-1 latency are predominantly associated with the 3’ LTR. **(A)** Examples of the major classes of chimeric transcripts associated with the 3’ LTR, where the HIV sequences start before, at or after the 3’ LTR transcriptional start site (TSS), the number of each detected is shown in brackets. All possible combinations with human gene regions (intron, exon, exon (3’ UTR), lncRNA Intron, intergenic or unannotated) and human sense are represented. Chimeras that made use of the HIV SD4 site at the 3’ end of the first encoding exon of Tat is shown in **(B)**. The number of bp of Tat sequence that was detected in each read is shown on the left, where the full exon encoding 72 amino acids of Tat is 215 bp **(C)**. All chimeras associated with the frequently used cryptic splice donors (NL4-3 nucleotides 583: cSD1 and 9388: cSD11). **(D)** The sequence for the cryptic splice donor site at nucleotide 583 and comparison of the degree of similarity of this sequence and the two major HIV canonical splice donors to the human consensus sequence. The human sequences are sense, unless indicated with AS (= antisense). Human sequences are depicted in blue (dark = exon/lncRNA, light = intron), brown (intergenic) or grey (unannotated). All other colours represent HIV sequences as indicated in the corresponding diagram for each panel. In **(B, C)** human splice sites are usually canonical when the region of the gene associated with the chimera is an exon and cryptic when the associated region is an intron, intergenic or unannotated. The instances where an exon has been incorporated into the chimera through the use of a cryptic human splice site is indicated by [C]. UTR, untranslated region.

Most of the authentic chimeras were associated with the 5’ or 3’ integration sites (154/206). Six chimeras were associated with the 5’ LTR, with two of these antisense for the HIV-1 sequences (**Supplementary Figure 6A**). The nature of the four sense transcripts downstream of the 5’ LTR sequence observed in the short reads is not known. Hence, transcripts such as the major species detected in the J-Lat6.3 sample by Sanger sequencing that contained *tat* sequence would not be revealed by the Illumina sequencing methodology used here. In comparison to the few transcripts detected that were associated with the 5’ LTR, there was a disproportionate number of chimeras annotated to the 3’ LTR, implying that other factors may facilitate generation of these mRNA species. The 148 3’ LTR chimeras were differentiated by where the HIV-1 sequence started in relation to the 3’ transcriptional start site (TSS) (**Figure 3A**). Examination of the 5’ end of the HIV-1 region in these reads showed that 132 (89.1%) of these have arisen from transcriptional activation at the TSS. Seven of these begin at the exact TSS in the 3’ LTR (nucleotide 9530 of NL4-3) and the other 125 start at nucleotides downstream of 9530. This is a tentative observation, however, as the short reads of the sequencing methodology preclude detection of the entire HIV-1 region in the chimera. The remaining 16 3’ LTR associated chimeras may be products of poly(A) signal readthrough in the 3’ LTR, however whether transcription started at the 5’ LTR or from an upstream cellular gene promoter cannot be determined. These results suggest that the 3’ LTR is activated more frequently than readthrough of the human gene into the 5’ end of the HIV-1 genome. 3’ LTR chimeras were most often associated with introns and orientation of HIV-1 in the same direction as the human gene, however, readthrough into exons and unannotated regions of the genome were also detected (**Figure 3A**). Chimeras formed from transcription initiation at the 3’ LTR TSS would not be useful for the production of functional Tat.

### Aberrant Splicing Facilitates the Production of Chimeric mRNAs

Two major HIV-1 splice donor sites, SD1 and SD4, were involved in the generation of chimeras through aberrant splicing (**Supplementary Figure 6B** and **Figure 3B**) and both of these have been previously implicated in the generation of chimeric mRNAs in productive (Sherrill-Mix et al., 2015) and latent infection of HIV-1 (Pinzone et al., 2019). Two lesser used splice donor sites, SD1b and SD1a, were also annotated as junctions (**Supplementary Figure 6B**). Use of these splice donors sites in the 5’ end of the HIV-1 genome would not favour the incorporation of Tat sequence into the final processed product. Twelve new cryptic splice donors that have not been previously reported were detected (**Supplementary Figure 5B**). Of these, 10 were each detected in a single read (**Supplementary Figure 4**) and require additional verification of their authenticity as analysis with prediction softwares yielded scores that do not support their existence (**Supplementary Data 1**). The remaining two, cryptic splice donor 1 (cSD1, nt 583 - 5’ LTR) and cryptic splice donor 11 (cSD11, nt 9388 - NEF/3’ LTR) were detected in 13 and 3 chimeras, respectively (**Figure 3C**). cSD1 shows a 78.98% similarity to the consensus human SD sequence and is better predicted as a splice donor site than several other published minor splice donors in the HIV-1 genome (**Figure 3D**; **Supplementary Data 1**). This observation, together with the high frequency of its detection and ten instances of splicing to canonical human splice acceptors, strongly supports the authenticity of this splice site. Indeed, this splice site has also been described in the recent paper that detected chimeric mRNAs in single cells from HIV-1 infected donors (Liu et al., 2020). In general, the chimeras that were generated through activation of HIV-1 splice donors made use of canonical human splice acceptors when associated with a human exon and use of cryptic splice acceptors when associated with a human intron or intergenic or unannotated regions of the genome.

Two chimeras that were generated through the use of minor splice acceptors in the HIV-1 genome (SA8, SA8a) were associated with cryptic splice donors in the human genome (**Supplementary Figure 6C**). An additional two appear to be the result of activation of cryptic HIV-1 splice acceptors (**Supplementary Figures 4** and **5B**). All four of these chimeras require further validation as indicated by the assessment of the similarity of the involved cryptic splice sites (both HIV-1 and human) to the consensus human splice donor or acceptor sequences (**Supplementary Data 1**).

Within the pool of authentic chimeras, 10 were associated with *tat* exon 2 (**Figure 3B**). Use of the SD4 splice site in conjunction with a human splice acceptor would preserve the first encoding exon of Tat. This has the potential to form chimeras that are able to translate a functional Tat protein, given that the 5’ end is intact in the original unfragmented mRNA. The chimera associated with PSMB2 would not allow this to occur as the Tat 5’ region is truncated through splicing of SD2 to SA5 (**Figure 3B**). In addition, a further 6 chimeras may also have an involvement of SD2 to SA5 splicing (LRCH3, DGCR2, DIP2A, OSTF1, RAP1A and CLECl1, **Figure 3B**) as the HIV-1 sequence in the reads all start at the SA5 region. A single SD4 associated chimera retained the full-length *tat* exon 2 (MACF1, **Figure 3B**) and is the only chimera detected in this dataset that has a high potential to facilitate production of functional Tat in its original full-length form. We also examined the SD4 associated chimeras reported by Pinzone et al. (2019), but could confirm that only two of the 21 transcripts were accurately formed through activation of HIV-1 SD4. The remainder did not show exact junctions at this splice donor site.

Taken together, these results show that aberrant splicing occurs with the involvement of a variety of both canonical and cryptic NL4-3 splice sites and results in the generation of chimeric cellular:HIV mRNA.

### 69 Chimeric mRNAs Were Detected From Two Infected Individuals on Suppressive Therapy

The Illumina workflow captures the junctions of chimeric transcripts, but very little of the sequence upstream or downstream from the junction site is available in the same read. In addition, the CCL19 primary cell model of HIV-1 latency does not completely recapitulate the *in vivo* complexity of HIV-1 integration. To address these limitations, PacBio Iso-Seq was used to detect chimeras in samples from six infected individuals on suppressive therapy. PBMC samples from an Australian based cohort of HIV-1 infected individuals who had been virally suppressed for at least 2 years were obtained through leukapheresis. These individuals were all male, with a median age of 53.5 and had between 6.8 – 176.6 copies of integrated HIV-1 DNA/10^6^ CCR5 (median 114.6) and 1.3 - 120.7 copies/μL of cell-associated unspliced (CA US) HIV-1 RNA (median 15.2) at baseline (**Supplementary Figure 7**). rCD4+ T cells were isolated by negative selection and magnetic depletion from the collected PBMCs and from these, whole cell RNA was extracted. A validated and tested approach using target enrichment and the PacBio platform to sequence rare RNA transcripts from low numbers of HIV-1 latently infected cells did not previously exist. To massively improve the chances of detection of HIV-1 sequence, adaptations were made to a general workflow provided by PacBio (**Supplementary Figure 8** **A**). Whole cell RNA was oligo-dT primed reverse transcribed then were uniquely barcoded for each donor during first-strand synthesis. After large-scale PCR amplification, the cDNA library underwent size selection to retain just the 1.5-5 kb fraction. This was chosen on the basis of canonical HIV-1 mRNA containing *tat* sequences being 1.8 kb in length and human mRNAs having an average length of ~2.5 kb (Piovesan et al., 2016). The 1.5-5 kb fraction was then amplified and hybridised to a custom designed probe library based on 12 reference HIV-1 clade B genomes and also HXB2 mRNA sequences that provide splice junctions matching those described in Purcell & Martin 1993 and Ocwieja et al., 2012. A final large-scale PCR was conducted prior to template preparation for sequencing on the PacBio RS II platform. In total, seven SMRT cell runs with the same cDNA library were conducted for this study.

Initial clean-up of raw reads was completed through the PacBio SMRT Portal bioinformatic platform, which involved the processing of polymerase reads to generate reads of inserts (ROIs) that could be used for alignment to the HIV-1 and human genomes (**Supplementary Figure 8** **B**). The final number of ROIs processed was 362,629, with the mean of the read lengths at 646.3 bp (**Supplementary Figure 8** **C**). This was an improvement over the read lengths of Illumina, which were ~200 bp on average after stitching of the 150 bp paired-end reads. In total only 0.073% (266 of 362,629) of ROIs aligned with high confidence to one or more of the strains in the panel of HIV-1 clade B reference genomes (AD8, BAL, BRU_LAI, 89.6 and HXB2). Of the 266 reads, 195 had HIV-1 sequences only and the remaining 71 aligned to both human chromosome sequences and the HIV-1 genome (**Supplementary Figure 8** **C**). Representing 26.69% of all reads aligning to the HIV-1 genome, the chimeric reads were proportionally much higher in this dataset compared to the study of chimeras in the CCL19-induced primary cell model of HIV-1 latency (0.01%) (**Supplementary Figure 9**). The reasons for this discrepancy have not been examined.

Reads that aligned only to the human genome were retrieved from all six donors and relative numbers of these reads for each donor was proportional to the amount of input cDNA in the hybridisation reactions (**Supplementary Figure 7**). HIV-1 sequence could not be recovered from 2 of the 4 donors (LKA002 and LKA011) and may be due to the low input of RNA/cDNA into the enrichment workflow or patient specific characteristics. LKA004 returned the highest number of total reads (38.36% of donor assigned reads), however almost all reads (73,479) aligned to human sequences and only 3 contained HIV-1 sequence. The CA US HIV-1 RNA levels at baseline in this individual is low compared to the other donors (e.g., 6.2 copies/μL vs LKA003 41.8 copies/μL, **Supplementary Figure 7**) and duration of treatment on the current regimen for LKA004 is 7 years longer than LKA003. LKA003 and LKA012 were the only donors to have successful detection of chimeric cellular HIV mRNA which is likely due to the fact that their baseline CA US HIV-1 RNA levels were the highest out of the six donors (41.8 and 120.7 copies/μL, respectively). Seventy HIV-1 only and 25 chimeric reads were assigned to LKA003 and 104 HIV-1 only and 46 chimeric reads were assigned to LKA012 (**Supplementary Figure 7**).

**Figure 4:**
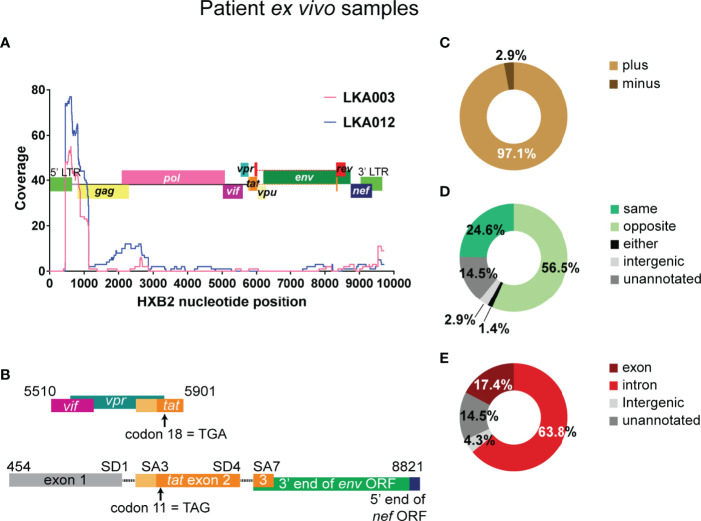
The 5’ end of the HIV-1 genome is detected very frequently in HIV-only reads from infected individuals on suppressive therapy. **(A)** In HIV-1 only reads, coordinates of best possible alignment to HXB2 was used to map coverage to the reference HIV-1 Clade B genome and shown as separate traces for the two donors from which the majority of the these reads were detected. The HXB2 genome is overlayed on the graph. **(B)** The structure of the two reads containing tat exon 2 sequence. In the absence of the full-length sequence it cannot be determined in the top sequence is derived from LTR-driven HIV-1 mRNA or if it belongs to a chimeric mRNA transcript. The bottom species represents an instance of HIV-1 5’ LTR initiated transcription and the use of authentic splicing (SD1 x SA3, SD4 x SA7) to generate the tat1 mRNA species, although it cannot be determined whether there is readthrough into the human genome at the 3’ end. Both transcripts appear to contain stop codons in the Tat encoding sequences, however, it is not clear if these are present in the provirus or arose during the template preparation and sequencing steps. The numbers indicate the positions within the HXB2 genome. Summary of genetic features of the authentic chimeric reads: HIV sense **(C)**, orientation of HIV integration relative to the chimera-associated human gene **(D)** and region of the human gene associated with the junctions in the chimeras **(E)**. The numbers shown are exact and not percentages.

The 5’ LTR beginning at the 5’ TSS (nucleotide 454) as well as the 5’ end of the *gag* region (nucleotides 790 to 1002) in the HXB2 genome had a coverage of 70 to 140 when considering all HIV-1 only reads in the dataset (**Figure 4A**; **Supplementary Figure 10** **A**), whereas a quarter of the genome received no coverage. This likely reflects the abundance of abortive transcripts that accumulate in latently infected cells rather than full-length transcription. At the other end of the genome, the last 179 nucleotides (9541 to 9719) of the 3’ LTR received coverage of between 15 to 20 in the whole dataset. The trend for the two donors, LKA003 and LKA012 from which the majority of HIV-1 only reads were detected was very similar (**Figure 4A**). When counting the number of representations of each open reading frame (ORF) in the HIV-1 only reads (with the possibility of a read containing sequences for more than one ORF), 5’ LTR and *gag* sequences were captured 133 and 127 times respectively (**Figure 4A**; **Supplementary Figure 10** **A**). All HIV-1 ORFs except for Vpu were represented in the HIV-1 only reads. Two *tat* sequence containing mRNAs were also detected (**Figure 4B**). It is unclear whether both transcripts arose from HIV-1 LTR derived mRNAs or are truncated sections of chimeric cellular:HIV mRNA. In addition, there are stop codons within the Tat coding regions (codon 11 and 18) of the reads, potentially indicating that these transcripts arose from Tat-defective proviruses.

### The Genetic Features of the Chimeras From Infected Individuals on Suppressive Therapy Are Similar to Those Detected in the Primary Cell Model Dataset

Altogether, 69 unique chimeras were detected from the two donors LKA003 and LKA012. The same pieces of genetic information obtained through the Illumina methodology could also be determined from PacBio sequencing. This dataset, however, is very small and cannot represent the full scope of the *in vivo* situation. Like the primary cell model dataset, the majority of reads incorporated the plus strand of HIV-1 (97.1%, **Figure 4C**).In addition, intergenic and unannotated regions were again associated with chimeras at low frequency (2.9% and 14.5%, **Figure 4D**), the human sequence was antisense more often than sense meaning that the orientation of HIV-1 integration relative to the human gene was more frequently observed to be in the convergent direction for chimeras detected from infected individuals on cART (56.5% vs 24.6%, **Figure 4D**). This was the only major difference with the primary cell model dataset where orientation was in the same direction. Introns were more frequently annotated than exons as the region incorporated into the chimeras (63.8% vs 17.4%, **Figure 4E**).

Junction sites were detected in 18 out of the 22 autosomal chromosomes, and in the X but not the Y chromosome (**Supplementary Figure 11** **A**). The percentage of junctions detected per chromosome was very similar to that observed in the primary cell model dataset (see **Figure 2D**) and again, had a positive correlation with the number of genes found on each chromosome (**Supplementary Figure 11** **B**, Spearman = 0.76, p<0.0001). This is consistent with the primary cell model dataset and the relatively random nature of HIV-1 integration at the chromosomal level.

### 3’ LTR Activation for Generation of Readthrough Transcripts Is Frequent in Latently Infected Cells From Infected Individuals on Suppressive Therapy

All chimeras detected made use of only 4 single nucleotide junctions mapped in the reference HXB2 genome (**Supplementary Figure 10** **B**). Nucleotides 1 and 9719 represent the 5’ and 3’ integration sites, respectively, while SD1 was the only canonical HIV-1 splice site annotated. The fourth junction involved activation of a cryptic splice donor, which was annotated at nucleotide 583 in HXB2, the identical location to that detected in NL4-3 for the primary cell model dataset (**Supplementary Figures 4** and **5B**).

**Figure 5:**
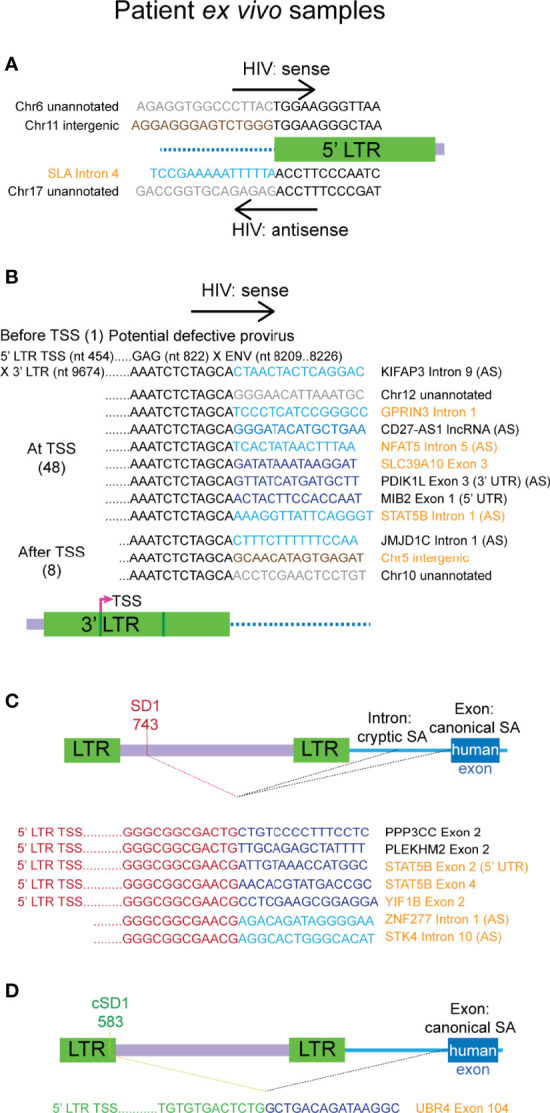
Authentic chimeras from the *ex vivo* samples of HIV-1 latency are predominantly associated with the 3’ LTR. **(A)** All chimeras detected that reflect the 5’ integration site, those with the plus strand of HIV (hv) are shown on top and the two with antisense HIV sequence (vh) are shown below. Examples of the major classes of chimeric transcripts associated with the 3’ LTR is shown in **(B)**, where the HIV sequences start before, at or after the 3’ LTR transcriptional start site (TSS), the number of each detected is shown in brackets. All possible combinations with human gene regions (intron, exon, exon (3’ UTR/5’ UTR), lncRNA, intergenic or unannotated) and human sense are represented. The single chimera with HIV-1 sequence starting before 3’ TSS appears to be a product transcribed from a defective provirus (top). All chimeras associated with the SD1 **(C)** cluster and the cryptic splice donor cSD1 **(D)** are shown. cSD1 is at the equivalent position in HXB2 to that detected in NL4-3 for the primary cell model of HIV-1 latency. The human sequences are sense, unless indicated with AS (= antisense). Human sequences are depicted in blue (dark = exon/lncRNA, light = intron), brown (intergenic) or grey (unannotated). All other colours represent HIV sequences as indicated in the corresponding diagram for each panel. Genes names and gene region descriptions that are highlighted in orange indicate chimeras detected from LKA003 and those in black are from LKA012. In **(C, D)** human splice sites were all canonical when the region of the gene associated with the chimera was an exon and cryptic when the associated region was an intron. UTR, untranslated region.

Out of the 69 unique chimeras, 61 were associated with either the 5’ or 3’ integration sites of HXB2 (**Supplementary Figure 10** **B**). In concordance with the preference of HIV-1 integration into introns of actively transcribed genes, 42 of the 61 chimeras were associated with the intron of a human gene. Both antisense and sense HIV-1 sequences were associated with the 5’ LTR chimeras and these involved readthrough of intronic, intergenic and unannotated regions (**Figure 5A**). The Chr11 intergenic:5’ LTR chimera is associated with the RPLP2 gene, however, as shown in the figure, the sequences at the junction with HIV-1 are those immediately downstream of the gene. This chimera also appears to terminate in the 5’ LTR poly(A) signal which can be activated when downstream splice donors are mutated (Ashe et al., 1995; Ashe et al., 1997). A sample of the 57 3’ LTR chimeras are shown in **Figure 5B**, with instances of association with introns, exons and intergenic regions of the human chromosome. The majority of these chimeras (48 out of 57) start at the 3’ TSS (nucleotide 9539) of HXB2, whereas 6 additional chimeras start downstream of 9539. This strongly suggests that all of these chimeras are actually products of 3’ LTR activation and readthrough into the human region. Only one 3’ LTR associated chimera had sequences upstream of the 3’ LTR. Transcription started at the 5’ LTR TSS, transcribing into *gag*, then intervening sequences were skipped before incorporation of what is potentially *env* sequences, followed by skipping of more sequence before ending in the 3’ LTR and readthrough into the flanking cellular intronic sequence (**Figure 5B**, top chimera). It is not clear if this transcript was generated by splicing or is a transcript arising from a defective provirus with large internal deletions.

Seven chimeras were associated with SD1 site activation and use of a canonical human gene exon SA site occurred in 5 of these 7 chimeras (**Figure 5C**). These chimeras were on average, longer than those associated with the 3’ LTR, and all start at or downstream of the 5’ TSS (nucleotide 454). This indicates that they arose from authentic HIV-1 LTR mediated transcription but resulted in readthrough into the human gene at the 3’end of the HIV-1 genome. Alternative splicing then processed the transcripts to remove the majority of the HIV-1 genome, linking SD1 with human gene splice acceptors. The full-length proviral genome in these cases could potentially contain large deletions and may, therefore, be defective. In four of these reads, there were also many successive splice events between the downstream exons of the human gene. Consistent with the chimeras seen in the primary cell model dataset, SD1 chimeras generated through activation of a cryptic splice acceptor usually incorporate sequences of a human gene intron instead of an exon (**Figure 5C**, last two chimeras).

We again observed the activation of the same cryptic splice donor cSD1, annotated at nucleotide 583 in the NL4-3 genome, leading to the generation of an aberrantly spliced transcript in a latently infected cell from LKA003 (**Figure 5D**). In HXB2, this cryptic splice donor is annotated to the same nucleotide number (**Supplementary Figure 5B**). The sequence aligns identically to both LTRs of HXB2, however, which would result in annotation to nucleotide 9668 as well. The chimeric transcript detected was generated through activation of a canonical human splice acceptor, hence providing more supporting evidence for the authenticity of this cryptic splice donor. This LTR sequence aligned with high confidence to other sequences assigned to LKA003 and had substantial mismatches to the NL4-3 LTRs, verifying that this transcript was not a contaminant from the previous template preparation process with the primary cell model samples.

No cellular:*tat* chimeras were detected in the samples from individuals on suppressive therapy, the sampling for this rare species may be limited by the small number of patients tested and high level of defective proviruses in patients on cART (Ho et al., 2013; Bruner et al., 2016). Overall, the two major patterns of chimera formation observed (3’ LTR or SD1 activation) are non-permissive for the incorporation of *tat* sequences into the processed transcript.

### The Frequently Reported HIV-1 Integration Site Associated Gene, STAT5B Was Annotated in Three Distinct Chimeras

Several studies in the past have examined integration sites of HIV-1 in samples from infected individuals on suppressive therapy. STAT5B has been reported in all three studies we examined (Ikeda et al., 2007; Maldarelli et al., 2014; Wagner et al., 2014) and is of particular interest because of its putative link to clonal proliferation of cells carrying integrated latent HIV. In addition, two studies examining aberrant splicing caused by HIV-1 integration reported the observation of STAT5B-associated chimeras in primary cells infected with NL4-3 (Pinzone et al., 2019) and cells from infected individuals (Cesana et al., 2017). In our study, STAT5B was annotated in three distinct chimeras assigned to LKA003 (orange chimeras, **Figures 5B, C**), where at least two separate integrants of HIV-1 are responsible. Two of the chimeras resulted from incorporation of STAT5B through use of SD1 and are associated with HIV-1 integrated in the same orientation. The third STAT5B chimera is a product of readthrough from the 3’ integration site where the sequences of STAT5B are antisense and could not have arisen from the same proviral integration site as the other two chimeras.

Overall, in our *ex vivo* dataset, 56 genes were associated with chimeras, and of these 22 have been published previously as integration sites in individuals on suppressive therapy (Ikeda et al., 2007; Maldarelli et al., 2014; Wagner et al., 2014) while 5 were reported to be associated with chimeras derived from integrated lentivectors (LV) in cell lines (Cesana et al., 2012; Moiani et al., 2012) (**Supplementary Figure 12**, **Supplementary Data 2**). Due to the small sample sizes (56 and 16 genes), we did not see any overlap in our dataset with the recent study that examined cellular:HIV chimeras in HIV-1-infected individuals on suppressive therapy (Liu et al., 2020). Of 182 genes annotated in the chimeras derived from the primary cell model, 71 have been reported previously as integration sites detected from individuals on suppressive therapy and 15 were associated with chimeras isolated from cell lines stably integrated with LVs (**Supplementary Data 2**). Three genes, HELZ, NFAT5 and PDIK1L were detected in both our datasets. All detected genes associated with the authentic chimeras from the PacBio and Illumina sequencing are listed in **Supplementary Data 3**.

General classification into broad biological function GO slims of the genes associated with our authentic chimeras showed a slightly higher representation of genes involved in cell proliferation than the 13.2% reported in the past for HIV-1 integration sites (Maldarelli et al., 2014; Wagner et al., 2014; Sunshine et al., 2016). 15.4% of genes in the primary cell model dataset and 17.9% of the genes in the *ex vivo* dataset had annotated functions in regulation of cell proliferation (**Supplementary Figure 13**). The differences here may be attributed to the specific subset of latently infected cells that were sampled for our investigations, where it is assumed that the integrations lie in genes that are being actively transcribed and are capable of permitting readthrough transcription. When statistical overrepresentation of biological function GO annotations were assessed with PANTHER, no class of genes were found to be significantly enriched in the genes annotated for chimeras detected from infected individuals on cART. In the authentic chimeras from primary cells, genes involved in regulation of mitotic cell cycle phase transition was found to be significantly enriched (28.07-fold, p=0.036) (**Supplementary Data 4**). Interestingly, genes associated with mRNA processing and metabolism were highly enriched in the set of genes annotated with the larger filtered Illumina dataset (**Supplementary Data 4**). Finally, we examined cryptic splice sites reported in the 5’ LTR in the LV used for the detection of chimeras in the study by Moiani et al. (2012) as well as those reported in patient defective proviruses by Pollack et al. (2017), but did not observe the equivalent splice sites in our chimeras. These may be strain-specific or dependent on the context of HIV-1 integration or require further sampling.

## Discussion

In our study of chimeric cellular:HIV mRNA in latently infected cells from the CCL19-induced primary cell model of HIV-1 latency and from infected individuals on cART, we showed that transcription is frequently activated at the TSS at the 3’ LTR and that formation of chimeric transcripts through aberrant splicing involving HIV splice donor sites is often observed. This agrees with previous studies that have examined chimeras from primary cell models (Pinzone et al., 2019) and patient samples (Cesana et al., 2017; Liu et al., 2020). We have also characterised a novel cryptic splice donor site in the HIV-1 genome that was detected from both the primary cell model and *ex vivo* samples and annotated to nucleotide 583 of both clade B strains, NL4-3 and HXB2. A recent study reported the detection of this cryptic splice donor, but did not highlight its significance as a novel splice site in the HIV-1 genome (Liu et al., 2020).

In the CCL19-induced primary cell model of HIV-1 latency, we detected 10 chimeras that were the products of aberrant splicing involving activation of SD4. This resulted in the incorporation of the 3’ end of Tat exon 2, which would result in C-terminal fusions to amino acids encoded by downstream gene exons. Depending on the reading frame of the human gene, these may result in chimeric proteins and Tat function may be impacted. Our investigation of chimeric cellular:*tat* mRNAs suggests that they are very rare (0.24% of unique chimeras) in latently infected cells of the CCL19 primary cell model, and their existence in *ex vivo* samples remains unknown. We speculate that lack of detection of cellular:*tat* mRNAs in *ex vivo* samples may be due to one or both of technical and biological factors. More efficient methods of enrichment or a higher depth of sequencing may allow the detection of these rare Tat-containing fusion transcripts. For detection of rare isoforms of human gene transcripts, it is estimated that 16-50 SMRT cell runs on the PacBio platform would have to be performed. In addition, sampling of PBMCs from more donors or different cohorts may increase the possibility of finding *tat* chimeric mRNAs. At the biological level, protein products expressed by chimeric cellular:*tat* mRNAs may be detrimental to cell survival or may lead to recognition and clearance of infected cells by the immune system in HIV-1-infected individuals on suppressive therapy.

In both the primary cell model and *ex vivo* samples, we observed a large contribution from 3’ LTR TSS activation to the HIV-1 RNA footprint associated with readthrough transcription. Past reports of activation of the 3’ LTR when the 5’ LTR is repressed supports these observations (Ashe et al., 1995; Greger et al., 1998; Lenasi et al., 2008). The activation of the 3’ LTR has no effect on HIV-1 latency as no viral protein products can be synthesised. This appears to be a phenomenon strongly associated with HIV-1 integrated in the opposite orientation to the human gene in patient cells, but not in the CCL19-induced primary cell model of HIV-1 latency. The study by Maldarelli et al. (2014) with samples from individuals on suppressive therapy also showed a weak but statistically significant preference for integration of HIV-1 in the opposite orientation to the human gene.

Twelve putative cryptic splice donors in the Clade B HIV-1 genomes (NL4-3 or HXB2) were identified in our study (**Supplementary Figure 5B**). A single cryptic splice donor (cSD1) was responsible for 14 species of chimeric cellular:HIV mRNA across both systems. Its presence in multiple chimeras derived from two vastly different types of latent HIV samples is a strong supporter of its authenticity. This is further reinforced by the fact that 11 of the 14 species detected were associated with the use of a canonical human splice acceptor to form the chimeras. In addition, this has been detected in a study of an unrelated cohort (Liu et al., 2020). Splicing involving the HIV-1 genome is a complex system and previously characterised splice sites, especially splice acceptors, do not always conform to those described for human gene sequences. In addition, major HIV-1 splice acceptors such as A2 and A3 are often intrinsically weak and are heavily regulated by *cis*-elements and *trans*-acting factors such as exonic splicing silencers (ESS) and hnRNP proteins that reduce their activation (Caputi et al., 1999; Bilodeau et al., 2001; Jacquenet et al., 2001; Kammler et al., 2006). As shown in past investigations of HIV-1 alternative splicing (Bilodeau et al., 1999; Carrera et al., 2010; Ocwieja et al., 2012), many minor splice sites in the HIV-1 genome have been reported. We detected a total of 60 chimeras formed through aberrant splicing and more than half of these (62.5%) involved the use of at least one cryptic splice site (human or HIV-1). This indicates that there is a significant disruption to canonical splicing patterns of the cell and weak cryptic splice sites may be activated with high frequency.

Aberrant splicing may change the function of the human protein associated with the integration site of HIV-1 or disrupt pathways in the cell. Evidence for this has been shown in the past where clinical trials with lentiviral vectors for correction of genetic disorders resulted in alterations to cell proliferation (Cavazzana-Calvo et al., 2010; Cesana et al., 2012; Moiani et al., 2012; Cesana et al., 2017). In *ex vivo* samples, integration of HIV-1 into NFATC3, a cancer-related gene, was shown to disrupt canonical transcription of the human gene downstream of the HIV-1 integration site (Liu et al., 2020). Several of the chimeras we detected that were formed through aberrant splicing (**Figure 3C**; **Supplementary Figures 6B, C**; **Figures 5** **C, D** **)** are associated with genes that have roles in malignant transformation or control of cell proliferation. These include the tumour suppressors, PDCD4, RB1, STK2, transcriptional regulators with reported roles in cancer, BCL9L, STAT5B and proteins that are involved in control of the cell cycle, ANAPC4, ANAPC5, RBX1. These aberrant transcripts may play a role in clonal proliferation or persistence of the HIV-1 latently infected clone. We also speculate that clonal expansion of these cells driven by aberrant splicing may have contributed to the easier detection of these chimeric transcripts as they would be produced by a larger population of cells.

In the model of transcriptional interference described for HIV-1 integrants in introns of actively transcribed genes, the opposite orientation is usually described as non-permissive for 5’ LTR-mediated transcription initiation and readthrough of RNA polymerase II (Han et al., 2008). This is true for most chimeras we detected, and this convergent orientation appears to favour 3’ LTR activation instead. A small subset of the chimeras generated through aberrant splicing, however, are products of HIV-1 integrants in the opposite orientation to the human gene. These incorporate introns in the chimeric transcripts through activation of cryptic splice acceptors. We also found an overwhelming majority of chimeras with HIV-1 sequences in the 5’ end of the read (vh), especially in those with aberrant splicing involved. Two factors may account for this. Firstly, as discussed above, the splice acceptors in the HIV genome are very tightly regulated and may not be active in aberrant splicing. This would favour the removal of the entire HIV-1 genome from the transcript. Secondly, there may be a significant contribution of reads that are initiating at the 5’ LTR and bypassing the 3’ LTR poly(A) signal. The 3’ poly(A) signal may be suboptimal or altered in these provirus sequences. This is the apparent mechanism that is giving rise to the SD1-associated chimeras from the patient samples. Production of readthrough transcripts and processing with involvement of SD1 would again, result in no perturbation to latency, and may be a characteristic of integrants of HIV-1 that are translationally, but not transcriptionally silent. Multiple post-transcriptional blocks to reactivation of latent HIV-1 including HIV transcriptional elongation, completion, and splicing have only gained attention recently (Yukl et al., 2018). The 3’ readthrough resulting in defective splicing of the HIV-1 genome constitutes another mechanism for maintenance of latency. Finally, we detected very few reads that were antisense for the HIV-1 region. Antisense transcription was estimated to only account for 0.9% of viral transcripts produced in actively infected cells (Lefebvre et al., 2011) and to a similar extent in latently infected cells based on the current study. This suggests that readthrough into the HIV-1 genome from a gene orientated in reverse appears to be very inefficient.

In four donors, 5’ LTR-associated HIV-1 mRNA was detectable, which agrees with recent the revision of HIV-1 latency as a heterogeneous phenomenon where proviruses reside in different states of silence (Baxter et al., 2016; Zhang et al., 2018). In contrast to the nature of the HIV-1 sequences detected in the chimeric reads from infected individuals on suppressive therapy, most HIV-1 only reads aligned to the 5’ LTR or gag ORF. This appears to reflect the high rates of abortive transcription that do occur from the HIV-1 promoter in latently infected cells. We aimed to sample the subset of the latent reservoir that contributes HIV sequences to the RNA footprint in the cell through readthrough transcription and aberrant splicing, however, full-length chimeric mRNAs were not isolated, even with the use of PacBio sequencing. Hence, the HIV-1 only reads cannot be concluded to all arise from 5’ LTR-mediated transcription since some of these may have been part of chimeric transcripts and cannot be annotated as such as the junction has not been captured. Knowledge of the matching DNA integration sites of the proviruses in these infected individuals would facilitate determination of the abundance of chimeric mRNAs relative to the number of intact proviruses. Defective proviruses have been extensively studied by other groups, are able to facilitate expression of aberrant protein products and appear to have a role in influencing the immune response against HIV-1 (Bruner et al., 2016; Imamichi et al., 2016; Pollack et al., 2017). This subset of proviruses is not clinically relevant for the aim of eradicating latent HIV, and their presence in the samples from infected individuals may confound our analysis of chimeric cellular:HIV mRNA. We were greatly limited from drawing conclusions based on the small number of infected donors from which chimeric mRNAs were detected. It is not known whether experimental or biological factors contributed to the lack of detection of chimeras from the other donors sampled.

In the three systems of HIV-1 latency studied, we observed highly variable levels of chimeric reads detected as a proportion of the reads aligning to the HIV-1 genome (**Supplementary Figure 9**). The two methods of HIV-1 mRNA enrichment and library preparation for sequencing used in this study rely on the same principles but some steps in the workflow may have an influence on the detection of chimeric reads. Library preparation for Illumina sequencing involves fragmentation of all RNA and reads that have only HIV-1 sequence or only human sequence may be fragments of chimeric transcripts. These cannot be annotated as such since the junction is not captured in the short reads yielded from the Illumina platform and hence, our detection of chimeras is an underestimate as described previously by Sherrill-Mix et al (Sherrill-Mix et al., 2015). Biologically, *ex vivo* samples are derived from individuals who have been on suppressive therapy for multiple years, whereas the primary cell model is expected to have higher rates of infection where it is more likely HIV-1 sequences will be detected. In addition, despite showing a negative reverse transcriptase (RT) activity in the supernatant, there may be a subset of cells still transcriptionally active at the HIV-1 LTR in the primary cell model.

**Figure 6:**
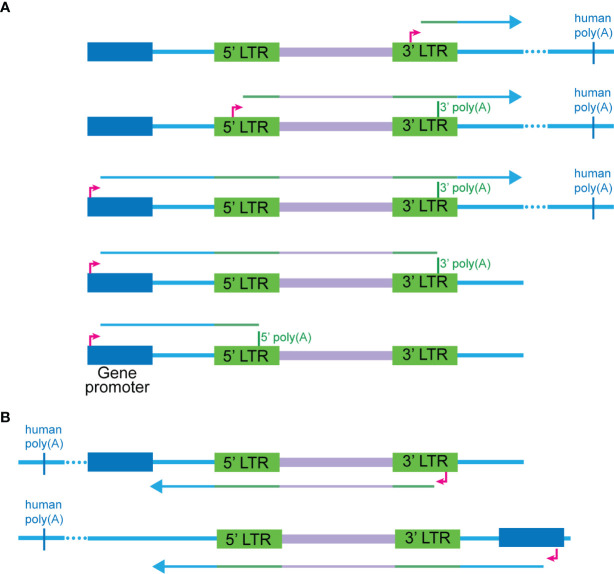
Modes of transcription that can give rise to cellular:HIV chimeras. **(A)** Five scenarios are possible for where the HIV sequence is sense, and the human sequence can be either sense or antisense. These are ordered from highest to lowest in the relative frequency detected in our study, although for most of the chimeras, the exact mode cannot be determined due to the lack of complete RNA transcripts. The first two modes involve transcription initiating at the 5’ or 3’ TSS of HIV respectively and reading through into the human sequence at the 3’ end of the gene. The third to fifth scenarios initiate at the human promoter and readthrough into the HIV-1 genome, before progressing to different extents: terminating at the 5’ poly(A), terminating at the 3’ poly(A) or reading through into the human sequence at the 3’ end. Scenarios one through three are most likely to give rise to vh chimeras including those generated by 3’ LTR readthrough into the flanking human sequences or HIV-1 splice donor to human splice acceptor aberrant splicing. Transcription in the first three scenarios will terminate at a human poly(A) sequence. **(B)** Two main scenarios are possible for where the HIV-1 sequence is antisense, and the human sequence can be either sense or antisense. The first involves transcription initiating at the HIV-1 antisense promoter in the 3’ LTR and then readthrough of the 5’ end of the HIV-1 genome into the human sequence. The second involves initiation of transcription from a human gene promoter and then readthrough into the HIV-1 genome from the 3’ end.

From our data, we observed five general modes of transcription that can give rise to chimeras with positive sense HIV-1 sequence (**Figure 6A**) and two modes for negative sense HIV-1 sequence (**Figure 6B**). These are described by the human or HIV-1 promoter used for transcription initiation and the HIV-1 poly(A) signal used for termination of transcription. The relative frequency of each mode is uncertain, although antisense HIV transcription is rare. In addition, some chimeras cannot be confidently assigned to only one specific mode. We speculate that within a given context of integration, transcription can only be initiated from specific promoters, and this is likely influenced by additional mechanisms related to transcriptional interference. In addition, aberrant splicing is also dependent on the environment surrounding the integrant of HIV-1, the interplay between human and viral splice sites and the presence of cellular splicing factors.

Our method facilitates the examination of chimeric cellular:HIV mRNAs using samples from HIV-1-infected individuals on suppressive therapy without perturbation of latency. The caveat here is that we are restricted to bulk RNA-Seq and cannot examine the cellular transcriptome of the individual, latently infected cells. Use of our target enrichment technique in parallel to other technologies, such as HIV-1 SortSeq (Liu et al., 2020), may add more dimensions to the study of HIV-1 latency.

Overall, we showed that 3’ LTR activation is a common phenomenon at the latent HIV provirus where integrants of HIV are often in reverse orientation to the human gene. In addition, aberrant splicing is detectable at low levels, using both cryptic and canonical splice sites in the HIV-1 genome. The major trends observed suggest at a lack of potential for HIV-1 protein expression through these pathways. Dysregulation of the gene involved in chimera formation may result as a consequence of aberrant splicing and may impact upon cellular pathways in which the gene is involved. In this study, we have generated a dataset that provides clues to the characteristics of latent HIV-1 integrants that promote the generation of chimeric cellular:HIV mRNAs. In addition, a better understanding of the HIV-1 RNA footprint in latently infected cells has been gained.

## Methods

### PBMC Isolation From Whole Blood and Negative Selection of rCD4+ T Cells

Buffy coats provided by the Australian Red Cross Blood Service (Southbank, Melbourne, Australia) were processed by use of Ficoll-Paque Plus (GE Healthcare Life Sciences) per manufacturer’s instructions to isolate Peripheral blood mononuclear cells (PBMCs). Washes were carried out with 2 mM EDTA pH 8.0 (Sigma Aldrich) in 1X PBS. For red blood cell (RBC) lysis, PBMCs were incubated with at least 3 mL of chilled 0.83% ammonium chloride (VWR International) on ice for a maximum of 5 minutes. Resting CD4+ (rCD4+) T cells were isolated by negative selection with an antibody cocktail targeting CD19, CD11b, CD14, HLA-DR, CD16, GlyA and CD69 Pure (Saleh et al., 2007) (incubation on ice, 40 minutes) where the positive fraction was depleted using LS or LD columns (Miltenyi Biotec) after incubation with goat anti-mouse IgG microbeads (Miltenyi Biotec) at 100 μL/10^8^ PBMCs (15 minutes at 4°C). All washes after RBC lysis were performed with PBS Deficient (PD) Buffer (140 mM NaCl, 2.7 mM KCl, 8 mM disodium orthophosphate (Na_2_HPO_4_) 1.8 mM potassium dihydrogen phosphate (KH_2_PO_4_) supplemented with 1 mM EDTA, 1% Heat-inactivated FBS (HI-FBS)). rCD4+ T cells were resuspended in RF10 after the final wash. The purity of rCD4+ T cells was assessed by flow cytometry by staining for Alexa Fluor 700-anti-human CD3 (1:20) (UCHT1, eBioscience/Affymetrix, Santa Clara, CA, USA), FITC-anti-human CD4 (1:50) (RPA-T4, eBioscience/Affymetrix), V450-anti-HLA-DR (1:50) (L243, BD Biosciences) and APC-anti-CD69 (1:125) (L78, BD Biosciences) on ice for 20 minutes. Analysis was done using the LSR II Flow Cytometer (BD BioSciences, San Jose, CA, USA).

### CCL19-Induced Latency and Treatment With Latency Reversing Agents

rCD4+ T cells isolated from healthy donor PBMCs were distributed into separate flasks for activation or CCL19 treatment, at concentrations of 1x10^6^ and 2x10^6^ cells/mL respectively. Recombinant human IL-2 10,000 U/mL (RayBiotech) and PHA 1 mg/mL (Remel/Thermo Fisher Scientific) at final concentrations of 10 U/mL and 10 μg/mL, respectively were added to the flask of cells for activation and incubated at 37°C. Resting cells were maintained in RF10 with 1 U/mL IL-2 at 37°C. The following day, the cells were treated with recombinant human CCL19 2.7 μM (R&D Systems) at 30 nM final concentration and placed at 37°C immediately for overnight incubation. Cells were infected with NL4-3 at a MOI of 0.1 (determined by radioactivity-based Reverse Transcription assay) for 2 hours at 37°C before a wash with PD. At four days post-infection, cells were reactivated with latency-reversing agents (LRAs) or treated with DMSO (Merck Millipore) alone. Cells were collected at 6, 24, 48 or 72 hours post-treatment, homogenised in TRIzol (ThermoFisher Scientific) and RNA extraction performed.

### SureSelect Target Enrichment and Illumina Mi-Seq

The custom probe library designed by Agilent Technologies was based upon the genome of the HIV-1 Clade B strain, NL4-3 (Genbank accession no. M19921). The probes were 120 bp long and spaced at 15 bp intervals along the entire genome (with a total pool of 438 distinct probes). Probes were enriched in the 5’ LTR and Tat exon 1 regions, and additional probes spanning the D1 to A3 junction were also included. Two independent experiments using this system was carried out. The protocol was conducted according to manufacturer’s instructions (outlined in the SureSelect^XT^ RNA Target Enrichment for Illumina Multiplexed Sequencing manual, Agilent Technologies) using the SureSelect Strand Specific RNA Library Prep and SureSelect Target Enrichment Kits. Additional reagents used were Agencourt AMPure XP (Beckman Coulter), Dynabeads MyOne Streptavidin T1 (Thermo Fisher Scientific) and Actinomycin D (Sigma-Aldrich). RNA extracted from Jurkat and J-Lat6.3 cells were used as controls in the target enrichment/sequencing workflow. Samples were pooled from several donors and time points after DMSO or drug treatment and between 200 ng and 4 μg of RNA was used for cDNA library preparation and target enrichment. Brief overview of the protocol: poly(A) RNA was purified from total RNA using oligo-dT magnetic particles, then chemically fragmented. This was followed by first-strand synthesis, second strand synthesis and end repair. The 3’ ends of the cDNA were then adenylated, followed by adapter ligation and subsequently amplification of the cDNA library before hybridisation. 100 ng of the cDNA libraries were carried through for hybridisation with the custom designed probe library and incubated for 24 hours at 65°C. cDNA library/capture library hybrids were captured using streptavidin beads, followed by post capture PCR indexing of samples to allow for multiplexed sequencing. Cluster amplification, preparation of templates for sequencing and sequencing of samples using the Illumina Mi-Seq RNA Sequencing platform was carried out by the Australian Genome Research Facility (Melbourne, Australia). Raw data received was 150 bp paired-end reads that were demultiplexed to generate separate datasets for the original samples.

### Thawing of Leukapheresis PBMC Samples and Isolation of rCD4+ T Cells

Leukapheresis samples were collected from HIV-1 infected individuals at the Alfred Hospital (Melbourne, Australia) with informed consent and under institutional guidelines. For maximum viability of thawed samples, the frozen PBMCs from 6 donors were processed according to the Immunovirology Research Network (IVRN, Australia) Laboratory Manual. Viability was determined by trypan blue (Thermo Fisher Scientific) staining. Isolation of rCD4+ T cells were done by using the T Cell Isolation Kit, human (Miltenyi Biotec) supplemented with purified mouse anti-Human CD69 and hybridoma supernatant containing α-HLA-DR antibodies (Clone 2-O6) (kindly provided by Associated Professor Paul Cameron, Peter Doherty Institute, Melbourne, Australia). Supplemented antibodies were bound with Anti-Mouse IgG Microbeads (Miltenyi Biotec). The negative fraction was collected by passing samples through LS columns (Miltenyi Biotec). Purity of rCD4+ T cells were assessed as described above. % rCD4+ T cell recovery ranged from 3% to 23%, and all recovered rCD4+ T cells were homogenised in TRIzol for RNA extraction.

### Roche/NimbleGen SeqCap Target Enrichment and PacBio RS II Iso-Seq

As the leukapheresis donors are an Australian-based cohort, these individuals are likely to be infected with strains of HIV-1 from clade B. We provided the sequences for 12 strains of clade B HIV-1 (Strain, Genbank accession no.: 89.6, U39362.2; AD8, AF004394.1; BAL, AB221005.1; BRU_LAI, K02013.1; HXB2, K03455; IIIB, KJ925006.1; MCK, D86068.1; MN, M17449; NL4-3, M19921; PM213, D86069.1; PV22, K02083.1; SF2, K02007.1) as well as the sequences of 69 mRNA species generated by HIV-1 that have been described previously (Purcell and Martin, 1993; Ocwieja et al., 2012). 50 bp probes spanning the entirety of the sequences provided were synthesised. Probes for the 5’ LTR, Tat exon 1 and Tat exon 2 regions were overrepresented by copy number in the total pool of probes. Three protocols provided by Pacific Biosciences were adapted for the workflow followed – 1. Isoform Sequencing (Iso-Seq™) Using the Clontech^®^ SMARTer^®^ PCR cDNA Synthesis Kit and BluePippin™ Size-Selection System, 2. Barcoding Samples for Isoform Sequencing (Iso-Seq™ Analysis) and 3. Full-length cDNA Target Sequence Capture Using SeqCap^®^ EZ Libraries. For the initial reverse transcription reactions, 10 reactions each with 1.5 μg of RNA were prepared for each donor. cDNA was generated as per manufacturer’s instructions with the SMARTer™ PCR cDNA Synthesis Kit (Clontech Laboratories/Takara Bio) with the 3’ SMART CDS Primer II-A replaced with each of the six individual barcoded Oligo-dT primers (Sigma-Aldrich, HPLC purified, see below). Samples were diluted with Elution Buffer (EB, Tris-HCl 10 mM pH 8.5, Invitrogen/Thermo Fisher Scientific). First-strand DNA was amplified with the KAPA HiFi PCR kit (Takara Bio) following manufacturer’s instructions where the Fidelity Buffer and 5’ PCR Primer IIA from the SMARTer kit were used. 16 reactions were done per donor sample with the following conditions: 95°C for 2 minutes, 16 cycles of 98°C for 20 seconds, 65°C for 15 seconds, 72°C for 4 minutes and a final extension of 72°C for 5 minutes. The PCRs for each donor were pooled and cleaned by addition of 1x volume of AMPure PB beads (Pacific Biosciences) which were allowed to bind DNA by shaking on the Eppendorf ThermoMixer C (Eppendorf) at 2000 rpm for 10 minutes at RT. The liquid was removed from the beads by incubation on MagneSphere Technology Magnetic Separation Stands (Promega) followed by two washes with 70% ethanol. DNA was eluted from the dried beads with 40 μL of EB by vortexing at 2000 rpm for 10 minutes at RT. Concentrations of DNA was determined using the Qubit 2.0 Fluorometer. Size selection was carried out on all the material obtained from the large-scale PCR reaction following instructions outlined in the BluePippin Operations Manual (Sage Science) using the 0.75% DF 1-6 kb Marker S1 cassette file to obtain our fraction of 1.5-5 kb (programmed cut-offs: BP start 1000 and BP end 5000). 40 μL of eluted DNA per sample run was collected from the BluePippin size selection process. 8 large scale PCR amplifications were done for each eluted sample following a similar cycling protocol to that described previously where 12 cycles instead of 16 and an extension time of 3 minutes were used, this was followed by purification with AMPure PB magnetic beads. The custom NimbleGen SeqCap EZ Library (Roche/NimbleGen) was used for hybridisation following manufacturer’s instructions. The cDNA from all six donors were pooled and distributed evenly for 6 separate hybridisation reactions and dried down with 1 μL of the SMARTer PCR Oligo and 1 μL of PolyT blocker (both at 1000 μM, see below) in a Savant DNA SpeedVac (Thermo Fisher Scientific) at RT. Hybridisation occurred at 47°C for 20 hours. The following day, DNA capture was done using Dynabeads M-270 Streptavidin (Thermo Fisher Scientific) and the SeqCap Hybridisation and Wash Kit (Roche/NimbleGen) following manufacturer’s instructions (NimbleGen SeqCap EZ Library User’s Guide). DNA was eluted off beads in 50 μL of EB where beads and eluate were both carried through into a final large-scale PCR reaction. For each target-enriched sample, 4 reactions were prepared with the KAPA HiFi PCR kit and SMARTer PCR Oligo (see below) using the following cycling conditions: 95°C for 2 minutes, 12 cycles of 95°C for 20 seconds, 65°C for 15 seconds, 72°C for 3 minutes 30 seconds, and a final extension at 72°C for 5 minutes. PCRs were cleaned up with 1x AMPure PB beads with incubations done at RT without a vortex mixer. Final elutions of cDNA from beads were done in 40 μL per hybridisation reaction. The elutions for all 6 hybridisations were pooled, quantified by Qubit and size distribution assessed on the Agilent 2100 Bioanalyzer and subsequently carried forward into cDNA SMRTbell Template Preparation and PacBio RS II Iso-form sequencing.

### Primers Sequences for PacBio Workflow

All primers were HPLC purified and purchased from Sigma-Aldrich

Barcode 1 (ODP3305) 5’-AAGCAGTGGTATCAACGCAGAGTACTCAGACGATGCGTCATTTTTTTTTTTTTTTTTTTTTTTTTTTTTTTVN-3’

Barcode 2 (ODP3306) 5’-AAGCAGTGGTATCAACGCAGAGTACCTATACATGACTCTGCTTTTTTTTTTTTTTTTTTTTTTTTTTTTTTVN-3’

Barcode 3 (ODP 3307) 5’-AAGCAGTGGTATCAACGCAGAGTACTACTAGAGTAGCACTCTTTTTTTTTTTTTTTTTTTTTTTTTTTTTTVN-3’

Barcode 4 (ODP 3308) 5’-AAGCAGTGGTATCAACGCAGAGTACTGTGTATCAGTACATGTTTTTTTTTTTTTTTTTTTTTTTTTTTTTTVN-3’

Barcode 5 (ODP 3309) 5’-AAGCAGTGGTATCAACGCAGAGTACGATCTCTACTATATGCTTTTTTTTTTTTTTTTTTTTTTTTTTTTTTVN-3’

Barcode 6 (ODP 3310) 5’-AAGCAGTGGTATCAACGCAGAGTACACAGTCTATACTGCTGTTTTTTTTTTTTTTTTTTTTTTTTTTTTTTVN-3’

SMARTer PCR Oligo (ODP 3311) 5’-AAGCAGTGGTATCAACGCAGAGTAC-3’

PolyT blocker (ODP 3312) 5’-TTTTTTTTTTTTTTTTTTTTTTTTTTTTTT/3INVDT/-3’

SMARTer II A from SMARTer PCR cDNA Synthesis Kit

(Clontech) 5’-AAGCAGTGGTATCAACGCAGAGTACGCGGG-3’

5’ PCR Primer II 5’-AAAGCAGTGGTATCAACGCAGAGT-3’

### Quantification of Total HIV-1 DNA, Integrated HIV-1 DNA and Cell-Associated (CA) Unspliced (US) HIV-1 RNA

High molecular weight DNA was extracted from patient cells using Qiagen AllPrep Kit where quantitative real-time PCR was performed with primers specific for the 5’LTR and gag. A separate nested real-time Alu-LTR assay was used to quantify integrated HIV DNA and normalization on cell equivalents was determined by detecting the copy number of CCR5 in a parallel reaction. Total cellular RNA was extracted from PBMCs using TRIzol (Life Technologies), converted to cDNA using random hexamers, oligo(dT) and Superscript III reverse transcriptase (Life Technologies) and CA US HIV-1 RNA quantified by quantitative real-time PCR. These assays were all performed as described previously (Lewin et al., 1999; Lewin et al., 2008; Elliott et al., 2014; Zerbato et al., 2021).

### Bioinformatic Analysis

#### Illumina RNA-Seq

The methodology used in the SureSelect^XT^ RNA sequencing library preparation workflow preserves strand information – Read 2 of each pair of paired-end reads corresponds to the original RNA poly(A) transcript. For stitching of paired-end reads: Read 1 is reverse-complemented and the overlap between the 3’ end of Read 2 and 5’ end of reverse-complemented Read 1 is found, where stitching was done if at least 10 bp of overlap was found. Each fragment (stitched reads) was separately aligned to HIV-1 (reference: NL4-3) and the human genome (hg19 assembly) using Bowtie 2 (Langmead and Salzberg, 2012) in local mode. Fragments found to align to both genome with mapping quality ≥ 1 were BLASTed (BLASTn (Altschul et al., 1990),) against a concatenated hg19 + NL4-3 genome. Fragments were deemed to be chimeras by using an e-value cutoff of < 1.0^-10^ and requiring an identity of at least 90%. Fragments for which there were more than one possible alignment to hg19 and the e-value ratio between the best and second-best human alignments were ≥ 1.0^-10^ were filtered out.

#### PacBio Iso-Seq

Raw polymerase read data were processed through the SMRT Portal (PacBio) platform using the RS_ReadsOfInsert protocol to generate Reads of Inserts (ROI) for mapping; filtering parameters of *minimum full passes* and *minimum predicated accuracy* were set at 1 and 90 respectively. ROIs were then aligned to the human and HIV-1 genomes as described above using Bowtie 2 followed by BLASTn. Five HIV-1 clade B reference genomes were used for alignment (89.6, AD8, BAL, BRU_LAI, HXB2).

#### Annotations of Chimeric Reads to Specific HIV-1 Regions and Analysis of Trends in HIV-1 Integration

Downstream analyses of chimeric reads were done using the Snapgene molecular biology software (GSL Biotech LLC, Chicago, Illnois, USA) and Microsoft Excel functions. The web-based gene set analysis toolkit [WebGestalt 2017 (Wang et al., 2017)] platform was used to classify genes by their GO Biological Process slim annotations.

#### Gene Ontology Analysis

Statistical overrepresentation test was performed using PANTHER (http://pantherdb.org/) (Mi et al., 2019) with the following settings: *Analysis Type:* PANTHER Overrepresentation Test (Released 20190701)*, Annotation Version and Release Date:* PANTHER version 14.1 Released 2019-03-12, *Database:* PANTHER GO-Slim Biological Process, *Reference genome*: Homo sapiens (all genes in database), Fisher’s Exact with Bonferroni correction for multple testing. Broad GO slim evaluation was performed using *WEB-based GEne SeT AnaLysis Toolkit/*WEB GESTALT (http://www.webgestalt.org/) (Liao et al., 2019) with the following settings: *Organism of interest:* Homo sapiens, *Method of interest:* Overrepresentation Enrichment Analysis (ORA), *Functional database:* Gene ontology + Biological process, *Reference gene list*: genome, *Advanced parameters*: default.

#### Splice Site Analysis

For assessment of similarity to human consensus splice sequences, we used the Analyzer Splice Tool (host-ibis2.tau.ac.il/ssat/SpliceSiteFrame.htm) (Carmel et al., 2004). For scoring of splice sites using the Maximum Entrophy Model, we used the MaxEntScan resource (Hollywood.mit.edu/burgelab/maxent/Xmaxentscan_scoreseq.html and Hollywood.mit.edu/burgelab/maxent/Xmaxentscan_scoreseq_acc.html) (Yeo and Burge, 2004).

### Cloning of PPP5C:5’LTR/*tat* Expression Constructs

J-Lat6.3 cells (NIH AIDS Reagent Program Division of AIDS, NIAID, Dr Eric Verdin) were maintained in Roswell Park Memorial Institute (RPMI) 1640 Medium (Gibco/Thermo Fisher Scientific) supplemented with 10% (v/v) Foetal Bovine Serum (FBS) (Gibco/Thermo Fisher Scientific) (RF10) and cultured at 37°C in 5% (v/v) CO_2_. The J-Lat6.3 clone contains one copy of the HXB2 HIV-1 genome (R7/E-/GFP) where the *env* contains a frameshift mutation and the *nef* ORF (nt positions 8796-9050) has been replaced with the eGFP gene (Jordan et al., 2003). After harvest of suspension cells, whole cell RNA was homogenised in TRIzol (ThermoFisher Scientific) and RNA extracted according to the manufacturer’s instructions. cDNA was synthesised with 500 ng – 5 μg of isolated whole cell RNA using SuperScript III Reverse Transcriptase (Thermo Fisher Scientific) per manufacturer’s instructions. Oligo(dT)15 primer (Promega) and random hexamers (Sigma-Aldrich) were used at 2 μM and 100 ng final for SuperScript III reactions respectively. When required, RNA was first treated with RQ1 RNase-free DNase (Promega) to remove contaminating DNA per supplier’s instructions. The GoTaq HotStart Polymerase Kit (Promega) was used for amplification where the 5X Green GoTaq Flexi Buffer, 25 mM MgCl_2_, 10 mM dNTPs were used at final concentrations of 1X, 1.5 mM and 0.2 mM, respectively in 50 μL reactions. The forward primer bound in PPP5C Exon 3 (ODP2631 5’-TGACAAGGATGCCAAAATGA-3’) and reverse primer in Tat exon 2, (ODP2630 5’-CGTCGCTGTCTCCGCTTCTTCCTGCCATAGGAGATGCC-3’) was used at final concentrations of 0.5 μM to amplify the chimeric transcripts. In general, the cycling conditions were: initial denaturation at 95°C for 2 minutes, followed by 35 cycles of 95°C (15 seconds), 45-72°C (15 seconds) and 72°C (1 minute/kb) and a final extension step of 5 minutes at 72°C. Additional primer sequences that were used for cloning of the derivative constructs using pNL4-3 (NIH AIDS Reagent Program, Division of AIDS, NIAID, NIH from Dr Malcolm Martin) or whole cell genomic DNA as template - for introducing LTR TAR C37T: ODP2835 5’-GACCAGATCTGAGCCTGGGAGTTCTCTGGCTAACTAGGGAACCC-3’ and ODP 2836 5’-GGGTTCCCTAGTTAGCCAGAGAACTCCCAGGCTCAGATCTGGTC-3; for reconstitution of Tat exon 2: ODP3173 5’-GGATCCACTAGTCCAGTGTGGTGGAATTCTGCAGATTTGCTTTGATAGAGAAGC-3’ and ODP 3174 5’-GGATCCACTAGTCCAGTGTGGTGGAATTCTGCAGATCTATTGCTTTGATAGAGAAGC-3’; and for reconstitution of PPP5C Exons 1 – 4: ODP3175 5’-GCGGCCGCCACTGTGCTGGATACGACACTTGTGCGGCAGCGGC-3’, ODP3176 5’-ATGACTACTTCAAAGCCAAGGACTACGAGA-3’, ODP 3177 5’-TCTCGTAGTCCTTGGCTTTGAAGTAGTCAT-3’, ODP 3178 5’-CGAGACTACGAGACGGTGGTCAAGGTGAAG-3’, ODP 3179 5’-CTTCACCTTGACCACCGTCTCGTAGTCTCG-3’, ODP 3180 5’-ACATCGAGAGCATGACCATTGAGGATGAGT-3’, ODP 3181 5’-ACTCATCCTCAATGGTCATGCTCTCGATGT-3’, ODP 3182 5’-CTGGTAGGCACATTTCCGGTGCAGT-3’.

#### Transfection of PPP5C:5’LTR/*tat* Expression Constructs

TZM-bl cells (NIH AIDS Reagent Program, Division of AIDS, NIAID, NIH, Dr John C. Kappes, Dr. Xiaoyun Wu and Tranzyme Inc.) were seeded into 24 well plates at 1x10^5^ cells per well in Dulbecco’s Modified Eagle’s Medium (DMEM) (MPU) supplemented with 1X GlutaMax™-1 (Gibco/Thermo Fisher Scientific) and 10% (v/v) Foetal Bovine Serum (FBS) (Gibco/Thermo Fisher Scientific) (DF10) and left overnight at 37°C in 5% (v/v) CO_2_ to reach 70-80% confluence for transfection. Lipofectamine 2000 (Life Technologies/Thermo Fisher Scientific) and DNA complexes (1.25 μL Lipofectamine/μg of DNA) were left for 20 minutes to form complexes at RT after dilutions of both components were prepared in separate tubes, left for 5 minutes at RT, and combined by drop-wise addition of DNA to the Lipofectamine 2000. Each well received 800 ng of DNA. CMV-LucR was co-transfected as a normalisation control for transfection efficiency. The transfection mixes were added dropwise to the wells of the plate and incubated at 37°C for 48 hours. After 48 hours, cells were lysed in 1X Passive Lysis Buffer (PLB) (Promega) and incubated for 15 minutes at RT on a rocking platform. 20 μL of the lysate was transferred to each well of a Costar^®^ 96-well white Microplate. 50 μL of each of the reagents in the DLR Assay Kit, LARII and Stop & Glo were injected into each well of the plate by use of the injectors in the FLUOstar Omega microplate reader where 35 second readouts were conducted with the injections timed at 10 (LARII) and 20 (Stop & Glo) seconds. Gain was set by first assaying additional wells with lysates from the positive control (or treatment wells known beforehand to have high luciferase activity).

## Data Availability Statement

The original contributions presented in the study are publicly available. This data can be found here: https://www.ncbi.nlm.nih.gov/sra/?term=PRJNA810450, NCBI Sequence Read Archive (SRA), Accession # PRJNA810450.

## Ethics Statement

The studies involving human participants were reviewed and approved by Alfred Hospital (HREC214/15). Protocol Version 6.0, University of Melbourne ethics letter of registration: Project number 1545227.1. The patients/participants provided their written informed consent to participate in this study.

## Author Contributions

GK, SS and DP contributed to conception and design of the study. ML, GK, SS and GC performed experiments, JM and SL obtained clinical samples, ML, GK, GC, TS, and MO organized the genomics database and ML and MO performed the bioinformatic and statistical analysis. ML wrote the first draft of the manuscript. GK, MO, SS and DP wrote sections of the manuscript. All authors contributed to the article and approved the submitted version.

## Funding

This study was supported by the National Health and Medical Research Council of Australia (NHMRC) program and project grants (#=GNT1052979 and GNT1129320 to DP and SL; and GNT1149991 to TPS for genomics), and Australian Centre for HIV and Hepatitis Virology Research (ACH2) (2016-17 to SS).

## Conflict of Interest

The authors declare that the research was conducted in the absence of any commercial or financial relationships that could be construed as a potential conflict of interest.

## Publisher’s Note

All claims expressed in this article are solely those of the authors and do not necessarily represent those of their affiliated organizations, or those of the publisher, the editors and the reviewers. Any product that may be evaluated in this article, or claim that may be made by its manufacturer, is not guaranteed or endorsed by the publisher.

## Supplementary Material

Supplementary Figure 1 Detailed workflow for SureSelectXT RNA NGS Target Enrichment Workflow and bioinformatics pipeline for detection of chimeric mRNAs

Supplementary Figure 2 Cryptic splice acceptor site used in the generation of PPP5C:HIV chimeras from J-Lat6.3 cells. RNA samples from latently infected cells with a known integration site (J-Lat6.3, PPP5C) were incorporated into the sequencing workflow to assess suitability of chosen methodology to detect chimeric cellular:HIV mRNA. **(A)** Yield of chimeras from Illumina RNA-Seq of J-Lat6.3 samples. **(B)** The single chimeric PPP5C:tat mRNA isolated and confirmed *via* Sanger sequencing from untreated J-Lat6.3 cells where a cryptic splice acceptor upstream of the HIV-1 integration site was activated, followed by splicing from SD1 to SA3 (bottoms). Several chimeric PPP5C:5’ LTR chimeras were detected *via* the NGS workflow described in **Figure S1**, where the major species observed (chimeric species 1. with splicing) utilised the same cryptic splice acceptor as that detected by Sanger sequencing (top). All other isolated species had varying lengths of the PPP5C intronic sequence upstream of the HIV-1 integration site, with no splicing observed within the short reads (chimeric species 2. no splicing). **(C)** The chimeric J-Lat6.3 mRNA sequence was introduced into a CMV-driven expression vector system with derivatives generated by reconstitution of the first encoding exon of Tat (72 amino acids), reconstitution of exons 1 to 4 of PPP5C and/or mutation of cytosine 37 to thymine in the TAR bulge of the 5’ LTR. Constructs were co-transfected into TZM-bl cells with constitutively expressed LucR and luciferase activity was measured 48 hours later. Luciferase Activity (LucF/LucR) is shown relative to Tat WT (first and second encoding exons, 86 amino acids). Data shows mean of three independent experiments, error bars = SEM.

Supplementary Figure 3 Filtering of reads for authentic chimeric mRNAs from the CCL19-induced primary cell model of HIV-1 latency. **(A)** Workflow for establishment of CCL19-induced HIV-1 latency in resting CD4+ T cells (rCD4+ T cells) isolated by negative selection and magnetic depletion of positive fraction from healthy donor peripheral blood mononuclear cells (PBMCs). rCD4+ T cells were treated with 30 nM CCL19 for two days prior to infection with NL4-3 (Saleh et al., 2007). Post-infection, cells were maintained in IL-2 at 1 U/mL for four days allowing for establishment of latent infection. Infected cells were treated with latency-reversing agents (LRAs) or DMSO on day 4 and collected at 6, 24, 48 and 72 hours post-treatment for RNA extraction. **(B)** Four samples with HIV-1 latency established through CCL19 treatment were sequenced over two independent experiments. **(C)** Outcome of overall bioinformatic alignment to HIV and human sequences. **(D)** Numbers of chimeras retained after each filtering step, bioinformatic (alignment to both HIV and human – Bowtie 2, BLASTn) and manual (mitochondria-associated, artifacts, duplicates).

Supplementary Figure 4 Breakdown of CCL19-induced primary cell model of HIV-1 latency derived chimeras by vh and hv arrangement. The chimeras detected in the primary cell model of HIV-1 latency can be further characterized by the order in which HIV-1 (virus) or human sequence appears in the read – these are described as vh or hv (v = virus, h = human); vh = virus at the 5’ end of the read and human at the 3’ end, hv = human at the 5’ end of the read and virus at the 3’ end. This nomenclature describes the direction of transcription or the readthrough event as well as the combination of splice sites used in generation of the chimeras. In the figure, the number of chimeras associated with each mapped single nucleotide junction in the NL4-3 genome for vh (left) and hv (right) chimeras is shown. IS, integration site; cSD, cryptic splice donor; cSA, cryptic splice acceptor; canonical splice sites are underlined. The NL4-3 coordinate for splice sites is given as the most 3’ nucleotide in the splice donor sequence and the most 5’ nucleotide in the splice acceptor sequence.

Supplementary Figure 5 Genome of HIV-1 with locations of previously reported splice sites and cryptic splice sites detected in this study. **(A)** The locations of canonical splice sites in the HIV-1 genome annotated according to Purcell & Martin 1993 and Ocwieja et al., 2012. **(B)** The locations of the cryptic splice sites detected in this study with the two most frequently detected, cD1 and cD11 shown in bold. In both NL4-3 (the strain of HIV-1 used to infect cells in the primary cell model) and HXB2 (the reference strain used for annotation of chimeras from *ex vivo* patient samples), cryptic splice donor 1 (cD1) is annotated to nucleotide 583. The genomes in this figure are based on NL4-3 with sizes of ORFs drawn to scale, the splice sites have also been indicated as close as possible to their location at the nucleotide level.

Supplementary Figure 6 Authentic chimeras from the CCL19-induced primary cell model of HIV-1 latency are predominantly associated with the 3’ LTR. **(A)** All chimeras detected that reflect the 5’ integration site, those with the plus strand of HIV (hv) are shown on top and the two with antisense HIV sequence (vh) are shown below. **(B)** All the SD1 chimeras associated with the SD1 cluster (use of SD1b, SD1 or SD1a). The only two hv chimeras that were generated through the activation of canonical HIV splice acceptors (SA8a and SA8) are shown in **(C)**.

Supplementary Figure 7 Metadata of HIV-1 infected leukapheresis donors on suppressive therapy.

Supplementary Figure 8 Workflow for detection of chimeric mRNAs in samples from HIV-infected individuals on suppressive therapy. Overview of the procedures followed for target enrichment and PacBio Iso-Seq for detection of cellular:HIV chimera mRNA transcripts in rCD4+ T cells from HIV-1 infected individuals on cART **(A)**. Bioinformatic pipeline for generation of reads of inserts (ROI) and detection of reads aligning to HIV-1 or aligning to both the HIV-1 and human genomes **(B)**. Summary of the output of bioinformatic processing and manual confirmation of HIV-1 aligning reads and chimeras **(C)**.

Supplementary Figure 9 Table showing summary of HIV aligned reads and chimeric reads in the three HIV-1 latency systems sequenced in this study

Supplementary Figure 10 Representation of HIV regions in PacBio reads from patient *ex vivo* samples. **(A)** In HIV-1 only reads, coordinates of best possible alignment to HXB2 was used to map coverage to the reference HIV-1 Clade B genome and the number of times each HIV region/ORF was detected is shown. **(B)** Single nucleotide coordinates corresponding to the HIV-1 junction site for each chimera are shown with the number of detected reads. cSD1 IS, integration site; cSD, cryptic splice donor; conventional splice sites are underlined.

Supplementary Figure 11 Chromosomes associated with the chimeric reads detected from HIV-infected individuals on suppressive therapy. The percentage of junctions represented by each chromosome for the two donors is shown by the coloured dots and mean of the two samples is shown by the black bar **(A)**. Correlation between number of genes per chromosome and percentage of junctions is shown in **(B)**, where only autosomal chromosomes were included. Number of genes per chromosomes obtained from Ensembl, release 91.

Supplementary Figure 12 Genes associated with cellular:HIV chimeras in the *ex vivo* dataset that have been reported as HIV-1 integration sites in previous studies. Genes that were also detected in our *in vitro* dataset are highlighted in the greyed out column on the right. The last column does not include the count in our *in vitro* dataset.

Supplementary Figure 13 Classification into major GO slim terms of genes found associated with chimeras. Genes associated with chimeras in the Illumina filtered **(A)**, Illumina authentic **(B)** and PacBio **(C)** datasets were put through gene ontology analysis for broad biological process terms using the WEB-based GEne SeT AnaLysis Toolkit and shown as number of genes associated with each class. The proportion of genes associated with cell proliferation is shown in each case.
