# Supplementary figures and images for: Detection of Chimeric Cellular: HIV mRNAs Generated Through Aberrant Splicing in HIV-1 Latently Infected Resting CD4+ T Cells
Source: Front Cell Infect Microbiol. 2022 Apr 28;12:855290. doi: 10.3389/fcimb.2022.855290 (PMC9096486; doi:10.3389/fcimb.2022.855290)

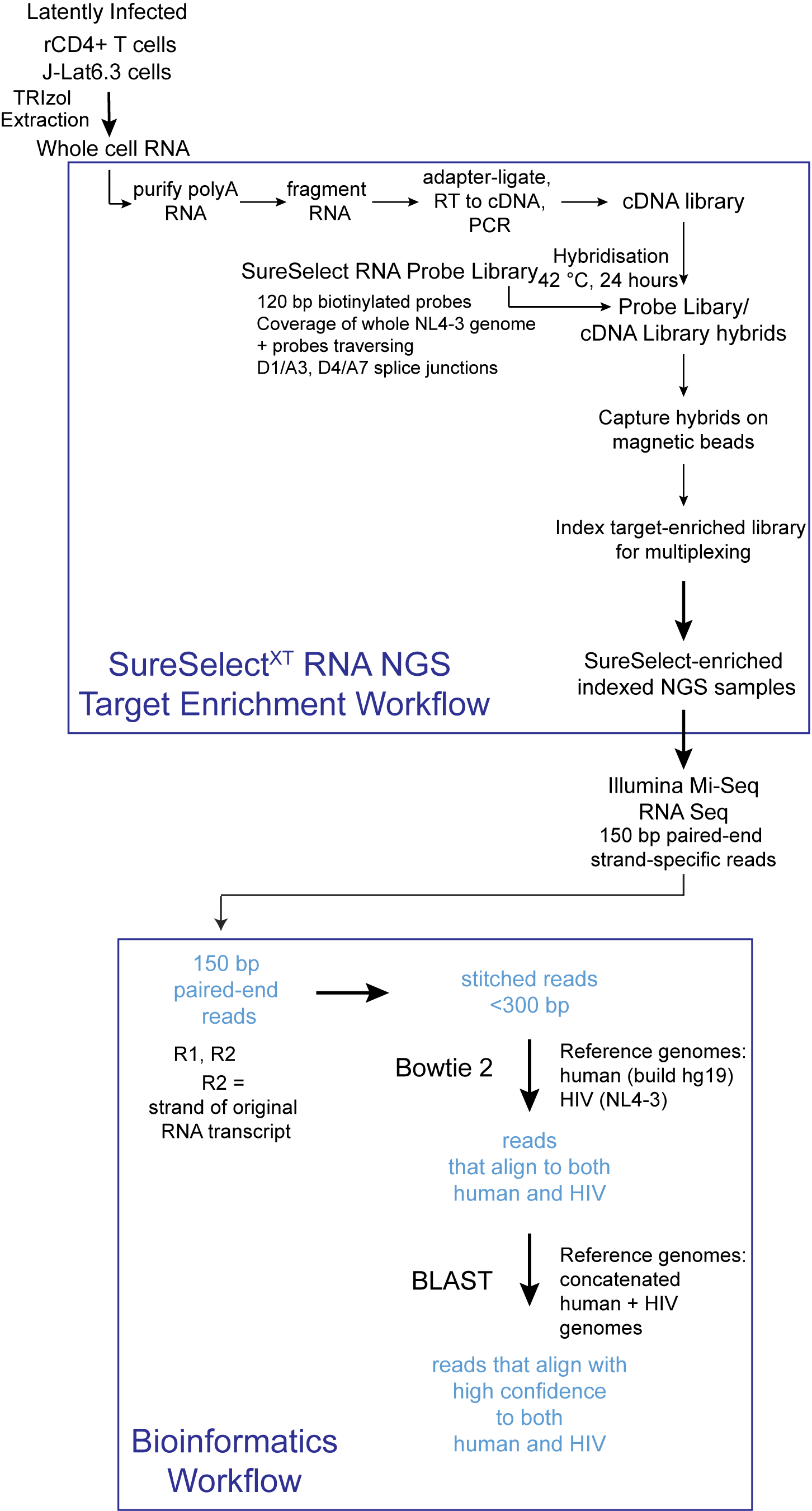

Supplement: Supplementary Figure 1 — Detailed workflow for SureSelectXT RNA NGS Target Enrichment Workflow and bioinformatics pipeline for detection of chimeric mRNAs [file Image_1.tif]

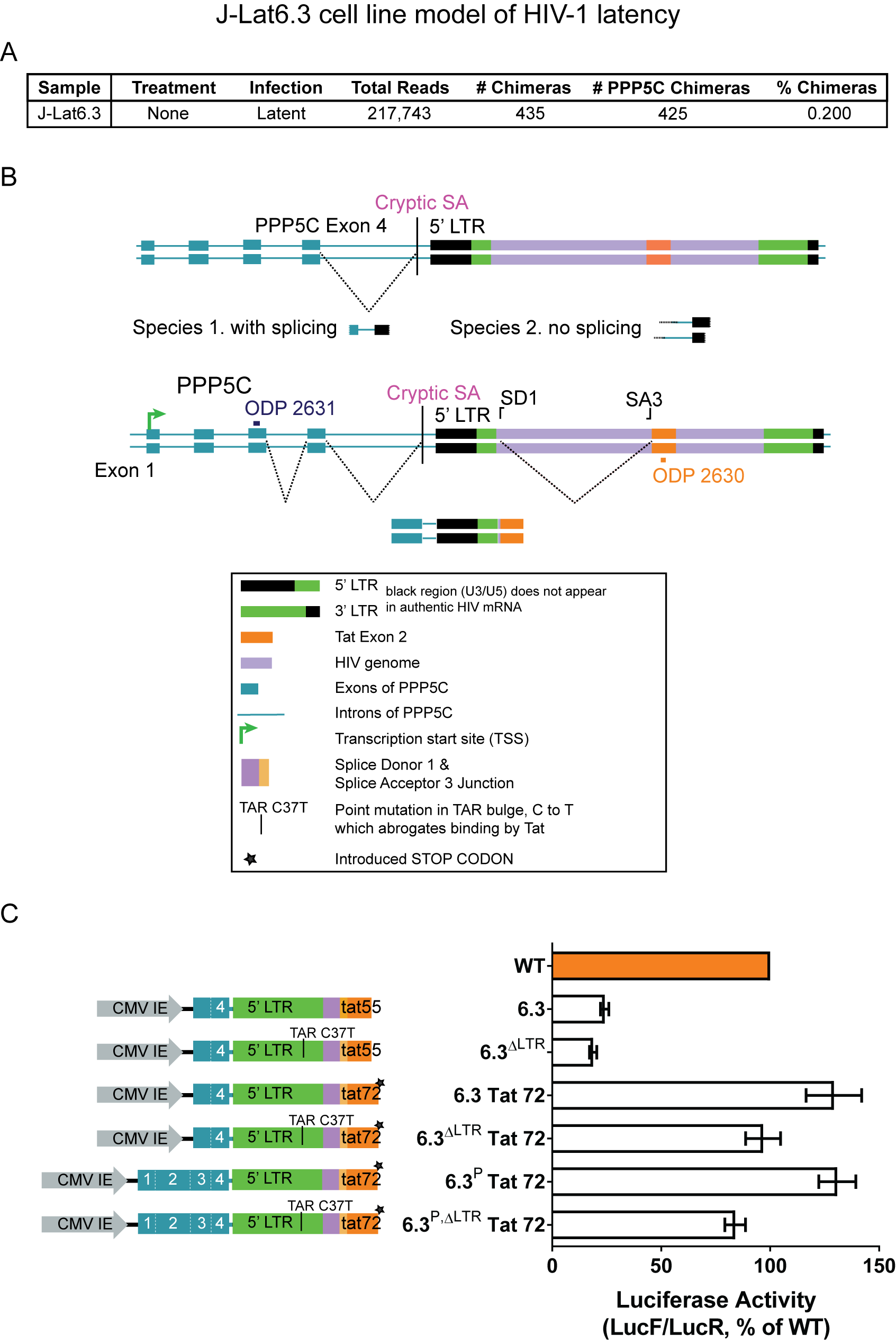

Supplement: Supplementary Figure 2 — Cryptic splice acceptor site used in the generation of PPP5C:HIV chimeras from J-Lat6.3 cells. RNA samples from latently infected cells with a known integration site (J-Lat6.3, PPP5C) were incorporated into the sequencing workflow to assess suitability of chosen methodology to detect chimeric cellular:HIV mRNA. (A) Yield of chimeras from Illumina RNA-Seq of J-Lat6.3 samples. (B) The single chimeric PPP5C:tat mRNA isolated and confirmed via Sanger sequencing from untreated J-Lat6.3 cells where a cryptic splice acceptor upstream of the HIV-1 integration site was activated, followed by splicing from SD1 to SA3 (bottoms). Several chimeric PPP5C:5’ LTR chimeras were detected via the NGS workflow described in Figure S1, where the major species observed (chimeric species 1. with splicing) utilised the same cryptic splice acceptor as that detected by Sanger sequencing (top). All other isolated species had varying lengths of the PPP5C intronic sequence upstream of the HIV-1 integration site, with no splicing observed within the short reads (chimeric species 2. no splicing). (C) The chimeric J-Lat6.3 mRNA sequence was introduced into a CMV-driven expression vector system with derivatives generated by reconstitution of the first encoding exon of Tat (72 amino acids), reconstitution of exons 1 to 4 of PPP5C and/or mutation of cytosine 37 to thymine in the TAR bulge of the 5’ LTR. Constructs were co-transfected into TZM-bl cells with constitutively expressed LucR and luciferase activity was measured 48 hours later. Luciferase Activity (LucF/LucR) is shown relative to Tat WT (first and second encoding exons, 86 amino acids). Data shows mean of three independent experiments, error bars = SEM. [file Image_2.tif]

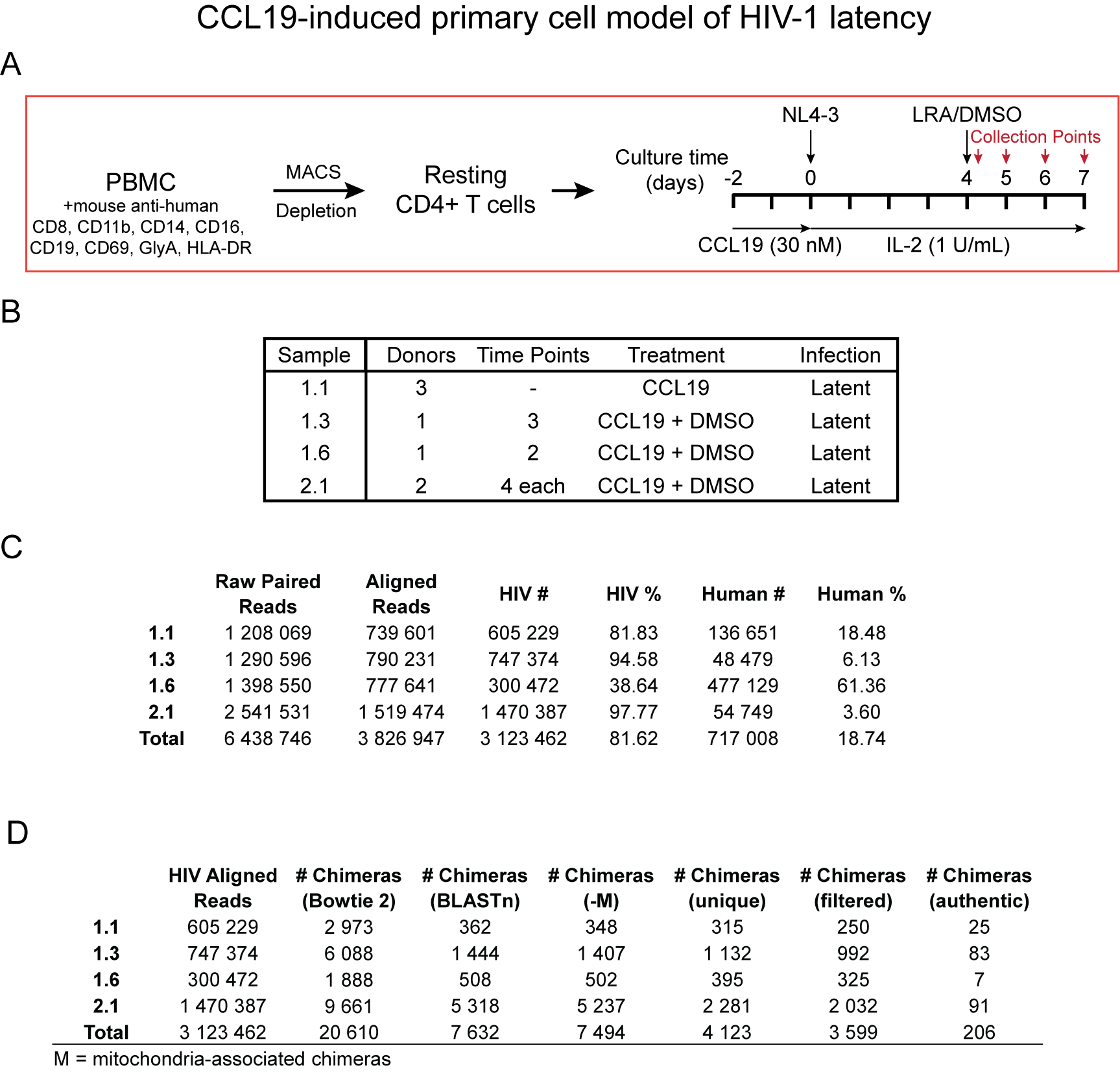

Supplement: Supplementary Figure 3 — Filtering of reads for authentic chimeric mRNAs from the CCL19-induced primary cell model of HIV-1 latency. (A) Workflow for establishment of CCL19-induced HIV-1 latency in resting CD4+ T cells (rCD4+ T cells) isolated by negative selection and magnetic depletion of positive fraction from healthy donor peripheral blood mononuclear cells (PBMCs). rCD4+ T cells were treated with 30 nM CCL19 for two days prior to infection with NL4-3 (Saleh et al., 2007). Post-infection, cells were maintained in IL-2 at 1 U/mL for four days allowing for establishment of latent infection. Infected cells were treated with latency-reversing agents (LRAs) or DMSO on day 4 and collected at 6, 24, 48 and 72 hours post-treatment for RNA extraction. (B) Four samples with HIV-1 latency established through CCL19 treatment were sequenced over two independent experiments. (C) Outcome of overall bioinformatic alignment to HIV and human sequences. (D) Numbers of chimeras retained after each filtering step, bioinformatic (alignment to both HIV and human – Bowtie 2, BLASTn) and manual (mitochondria-associated, artifacts, duplicates). [file Image_3.tif]

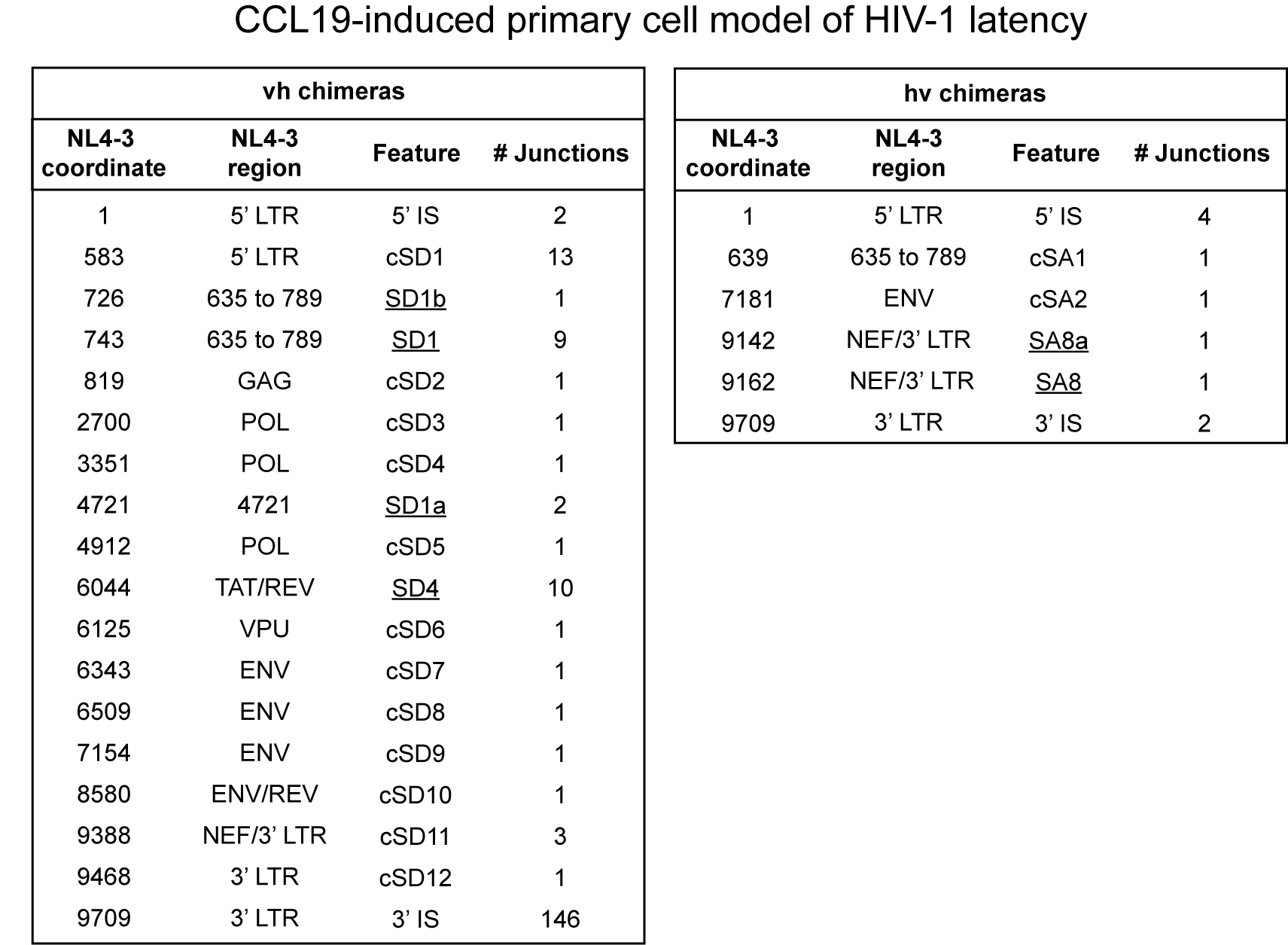

Supplement: Supplementary Figure 4 — Breakdown of CCL19-induced primary cell model of HIV-1 latency derived chimeras by vh and hv arrangement. The chimeras detected in the primary cell model of HIV-1 latency can be further characterized by the order in which HIV-1 (virus) or human sequence appears in the read – these are described as vh or hv (v = virus, h = human); vh = virus at the 5’ end of the read and human at the 3’ end, hv = human at the 5’ end of the read and virus at the 3’ end. This nomenclature describes the direction of transcription or the readthrough event as well as the combination of splice sites used in generation of the chimeras. In the figure, the number of chimeras associated with each mapped single nucleotide junction in the NL4-3 genome for vh (left) and hv (right) chimeras is shown. IS, integration site; cSD, cryptic splice donor; cSA, cryptic splice acceptor; canonical splice sites are underlined. The NL4-3 coordinate for splice sites is given as the most 3’ nucleotide in the splice donor sequence and the most 5’ nucleotide in the splice acceptor sequence. [file Image_4.tif]

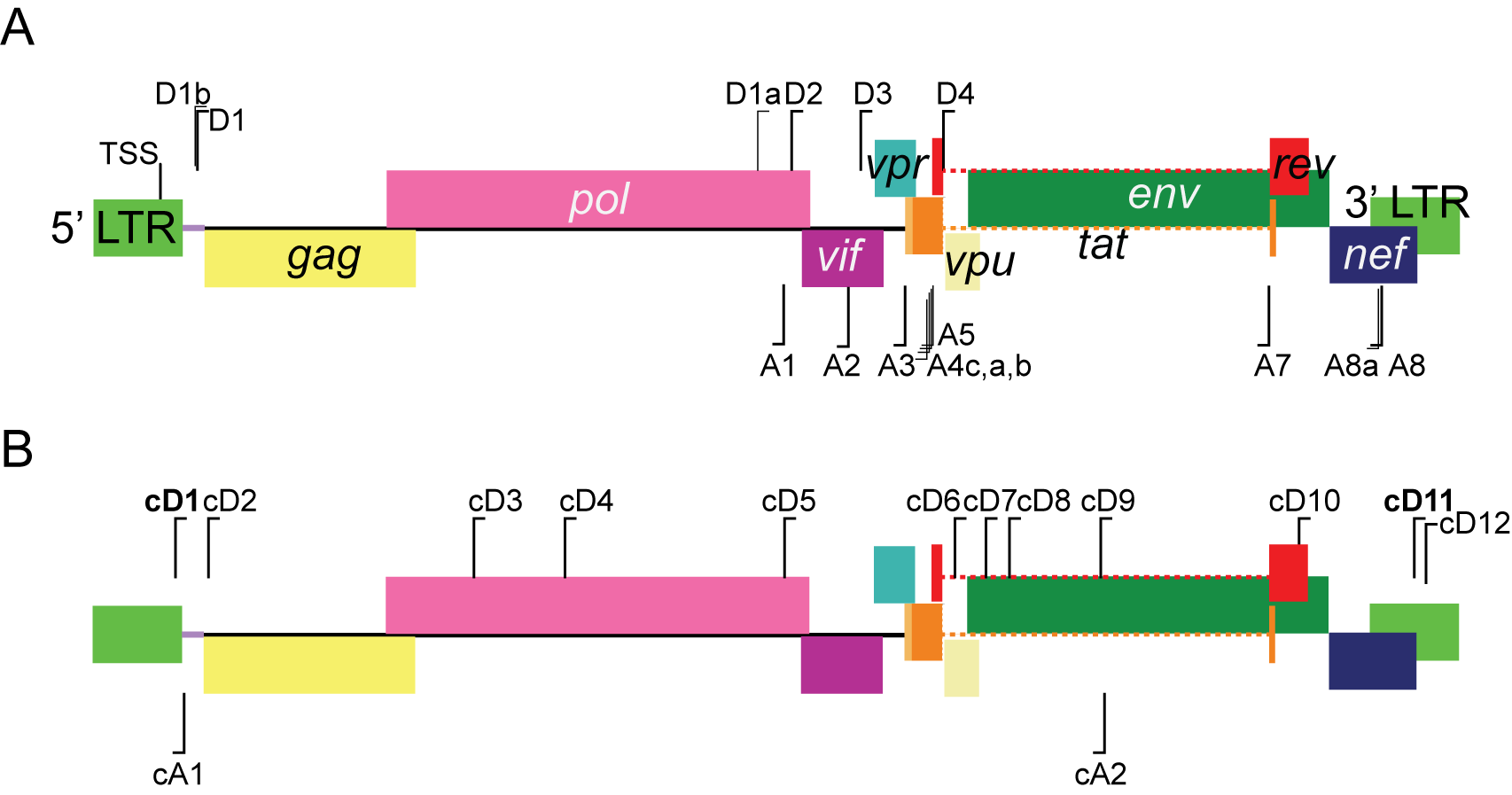

Supplement: Supplementary Figure 5 — Genome of HIV-1 with locations of previously reported splice sites and cryptic splice sites detected in this study. (A) The locations of canonical splice sites in the HIV-1 genome annotated according to Purcell & Martin 1993 and Ocwieja et al., 2012. (B) The locations of the cryptic splice sites detected in this study with the two most frequently detected, cD1 and cD11 shown in bold. In both NL4-3 (the strain of HIV-1 used to infect cells in the primary cell model) and HXB2 (the reference strain used for annotation of chimeras from ex vivo patient samples), cryptic splice donor 1 (cD1) is annotated to nucleotide 583. The genomes in this figure are based on NL4-3 with sizes of ORFs drawn to scale, the splice sites have also been indicated as close as possible to their location at the nucleotide level. [file Image_5.tif]

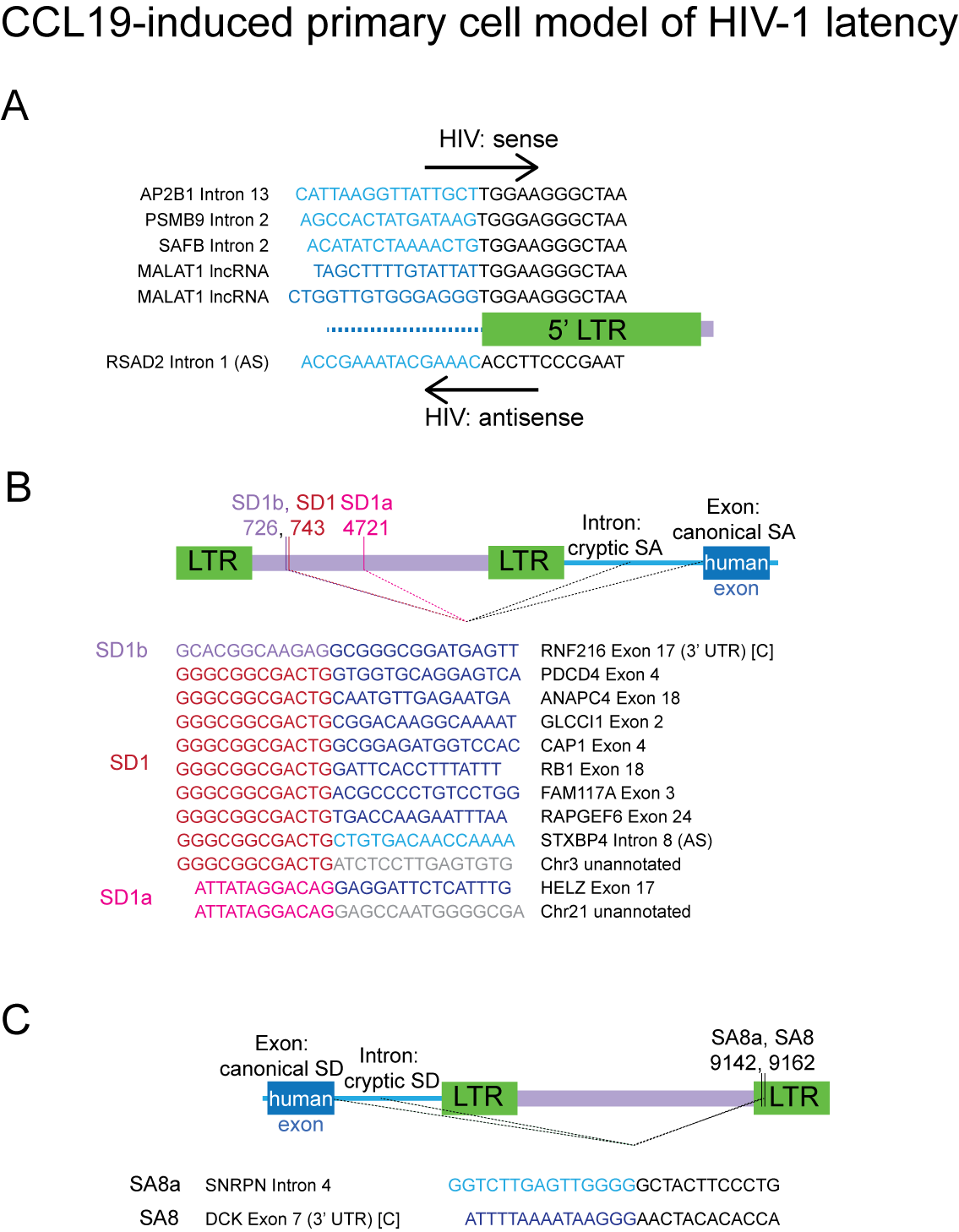

Supplement: Supplementary Figure 6 — Authentic chimeras from the CCL19-induced primary cell model of HIV-1 latency are predominantly associated with the 3’ LTR. (A) All chimeras detected that reflect the 5’ integration site, those with the plus strand of HIV (hv) are shown on top and the two with antisense HIV sequence (vh) are shown below. (B) All the SD1 chimeras associated with the SD1 cluster (use of SD1b, SD1 or SD1a). The only two hv chimeras that were generated through the activation of canonical HIV splice acceptors (SA8a and SA8) are shown in (C). [file Image_6.tif]

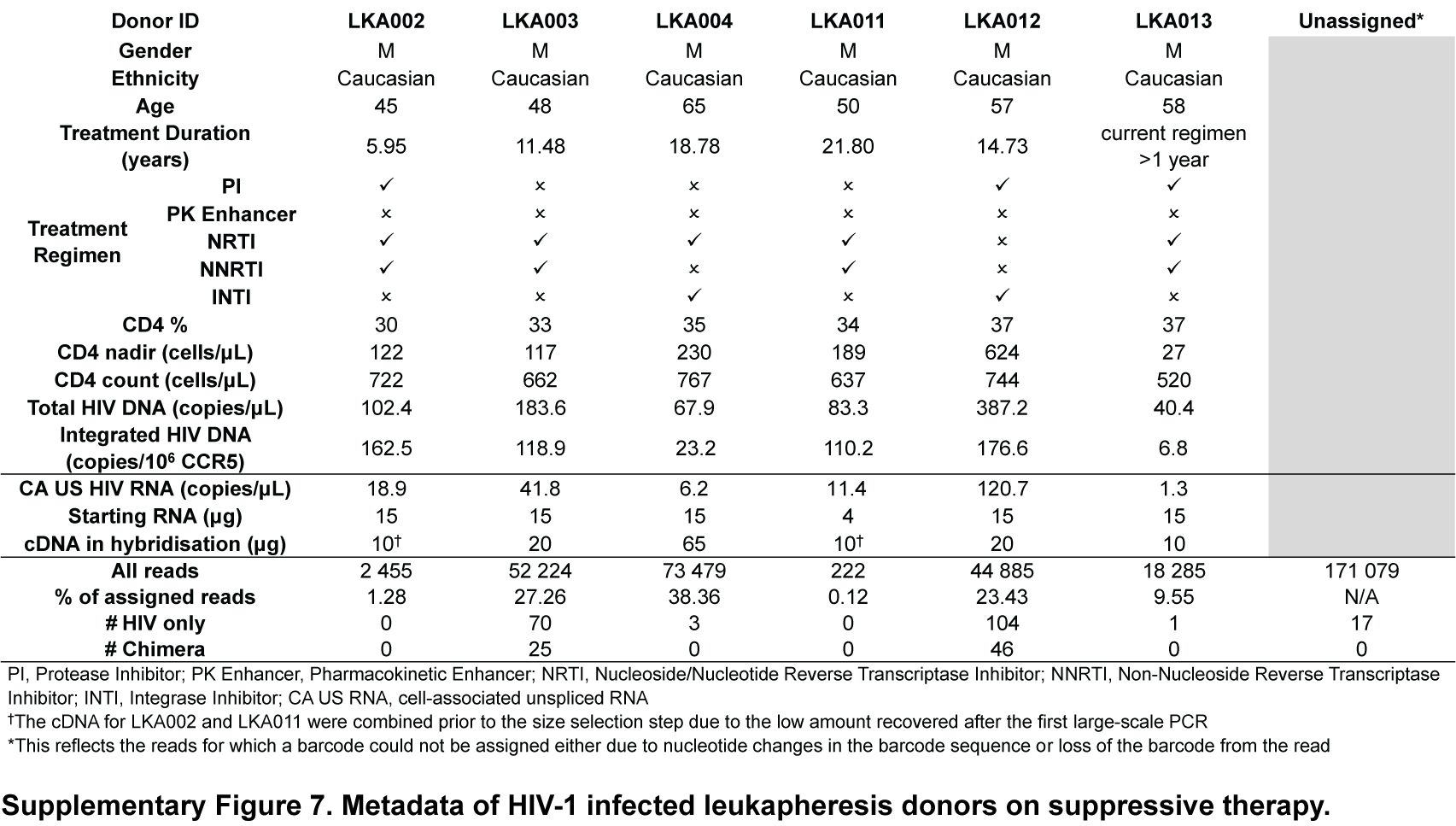

Supplement: Supplementary Figure 7 — Metadata of HIV-1 infected leukapheresis donors on suppressive therapy. [file Image_7.tif]

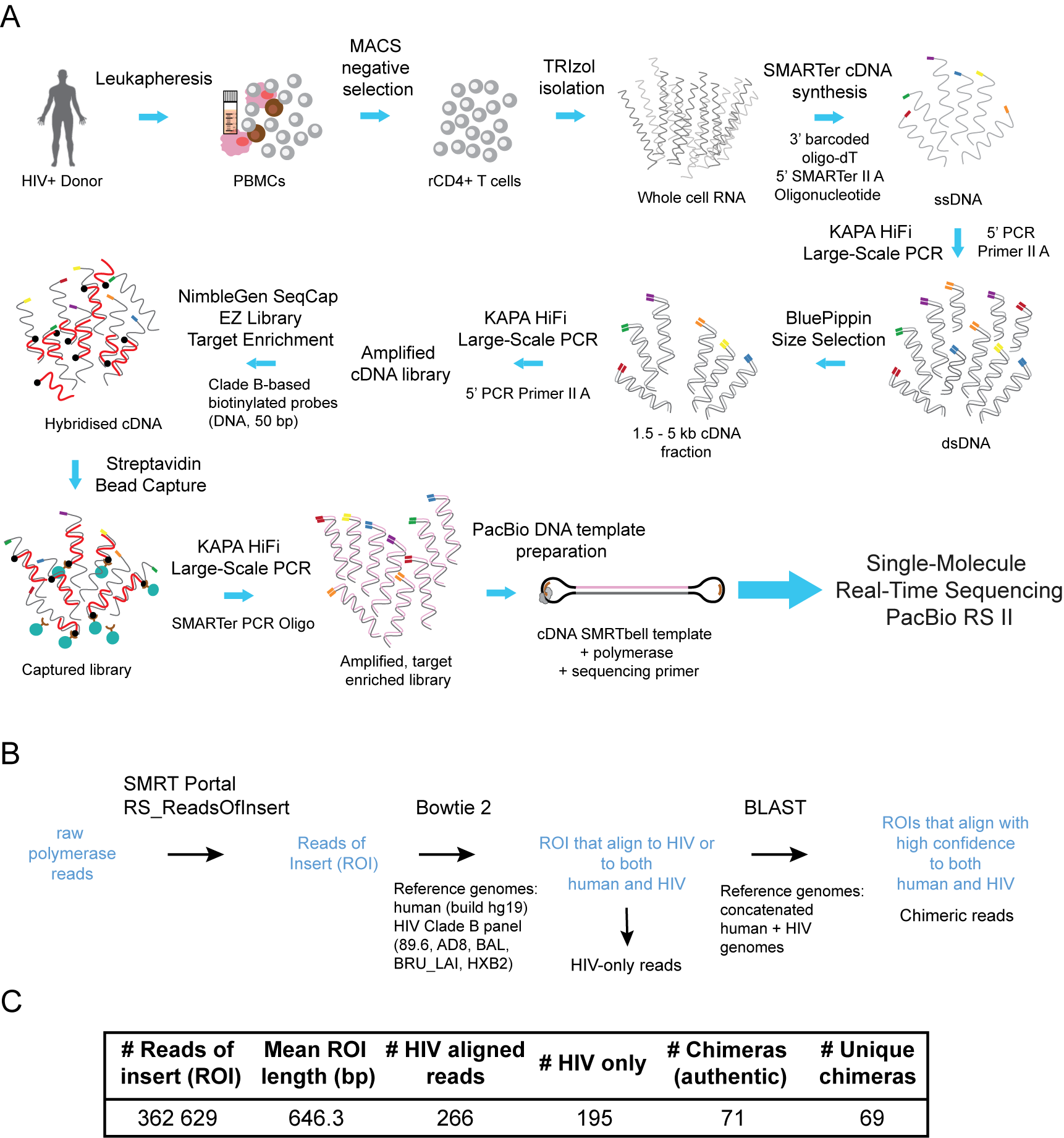

Supplement: Supplementary Figure 8 — Workflow for detection of chimeric mRNAs in samples from HIV-infected individuals on suppressive therapy. Overview of the procedures followed for target enrichment and PacBio Iso-Seq for detection of cellular:HIV chimera mRNA transcripts in rCD4+ T cells from HIV-1 infected individuals on cART (A). Bioinformatic pipeline for generation of reads of inserts (ROI) and detection of reads aligning to HIV-1 or aligning to both the HIV-1 and human genomes (B). Summary of the output of bioinformatic processing and manual confirmation of HIV-1 aligning reads and chimeras (C). [file Image_8.tif]

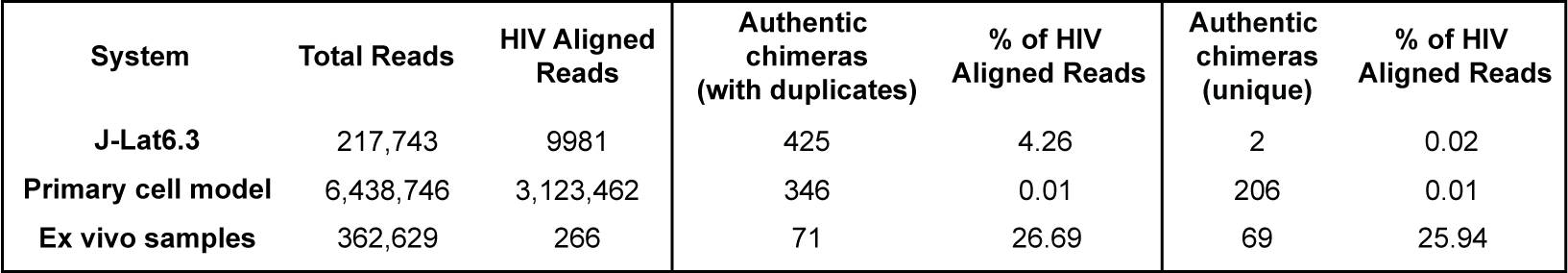

Supplement: Supplementary Figure 9 — Table showing summary of HIV aligned reads and chimeric reads in the three HIV-1 latency systems sequenced in this study [file Image_9.tif]

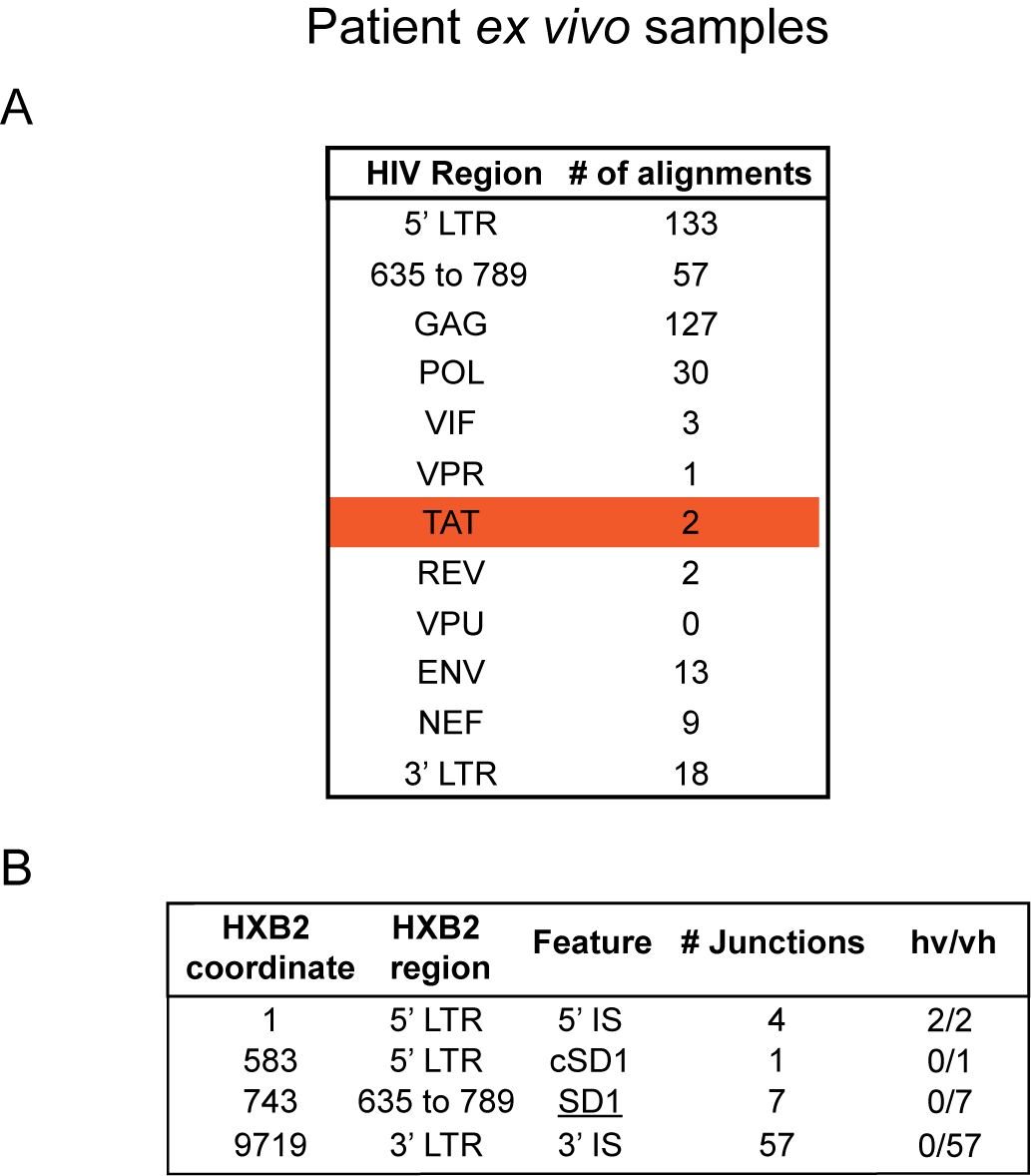

Supplement: Supplementary Figure 10 — Representation of HIV regions in PacBio reads from patient ex vivo samples. (A) In HIV-1 only reads, coordinates of best possible alignment to HXB2 was used to map coverage to the reference HIV-1 Clade B genome and the number of times each HIV region/ORF was detected is shown. (B) Single nucleotide coordinates corresponding to the HIV-1 junction site for each chimera are shown with the number of detected reads. cSD1 IS, integration site; cSD, cryptic splice donor; conventional splice sites are underlined. [file Image_10.tif]

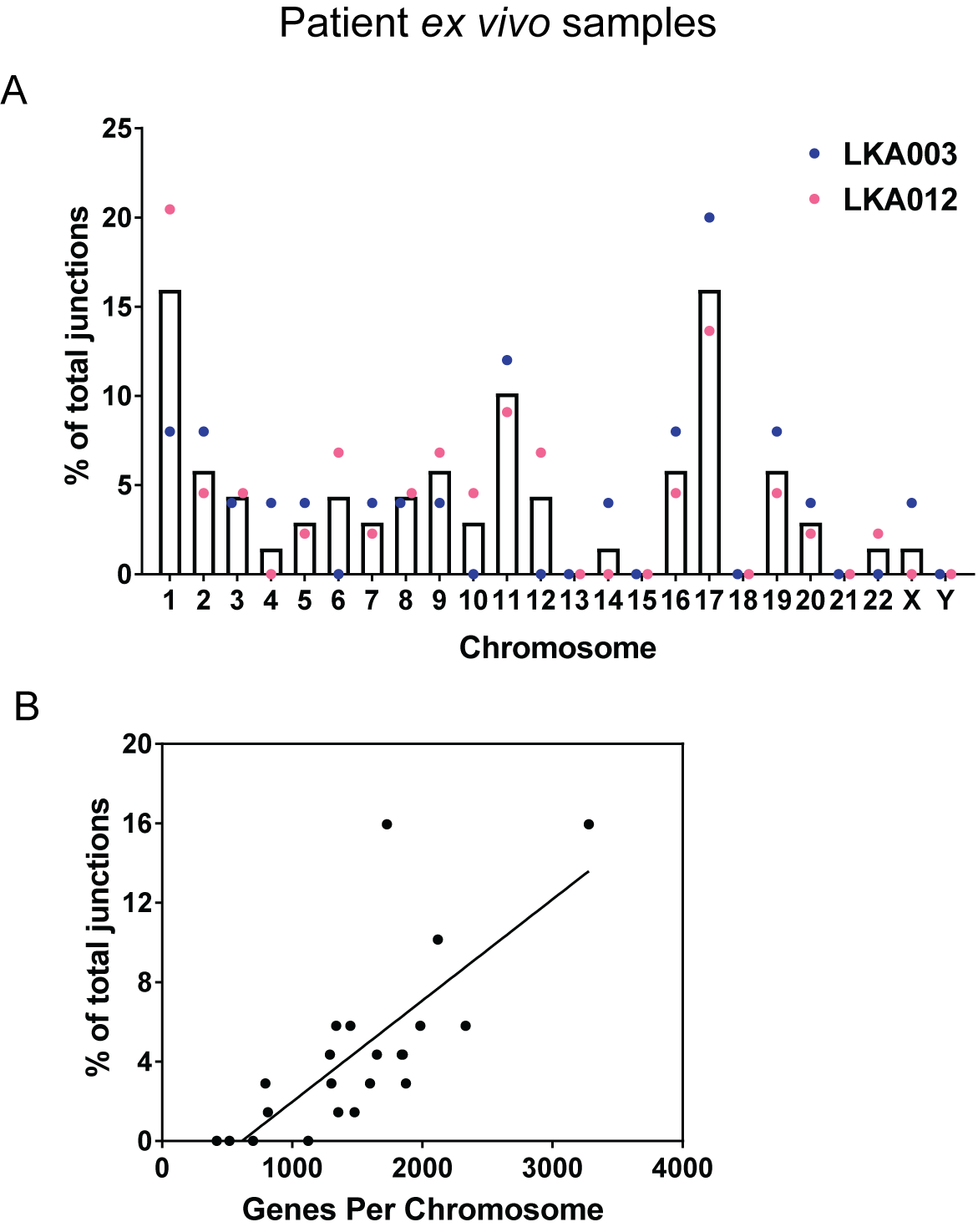

Supplement: Supplementary Figure 11 — Chromosomes associated with the chimeric reads detected from HIV-infected individuals on suppressive therapy. The percentage of junctions represented by each chromosome for the two donors is shown by the coloured dots and mean of the two samples is shown by the black bar (A). Correlation between number of genes per chromosome and percentage of junctions is shown in (B), where only autosomal chromosomes were included. Number of genes per chromosomes obtained from Ensembl, release 91. [file Image_11.tif]

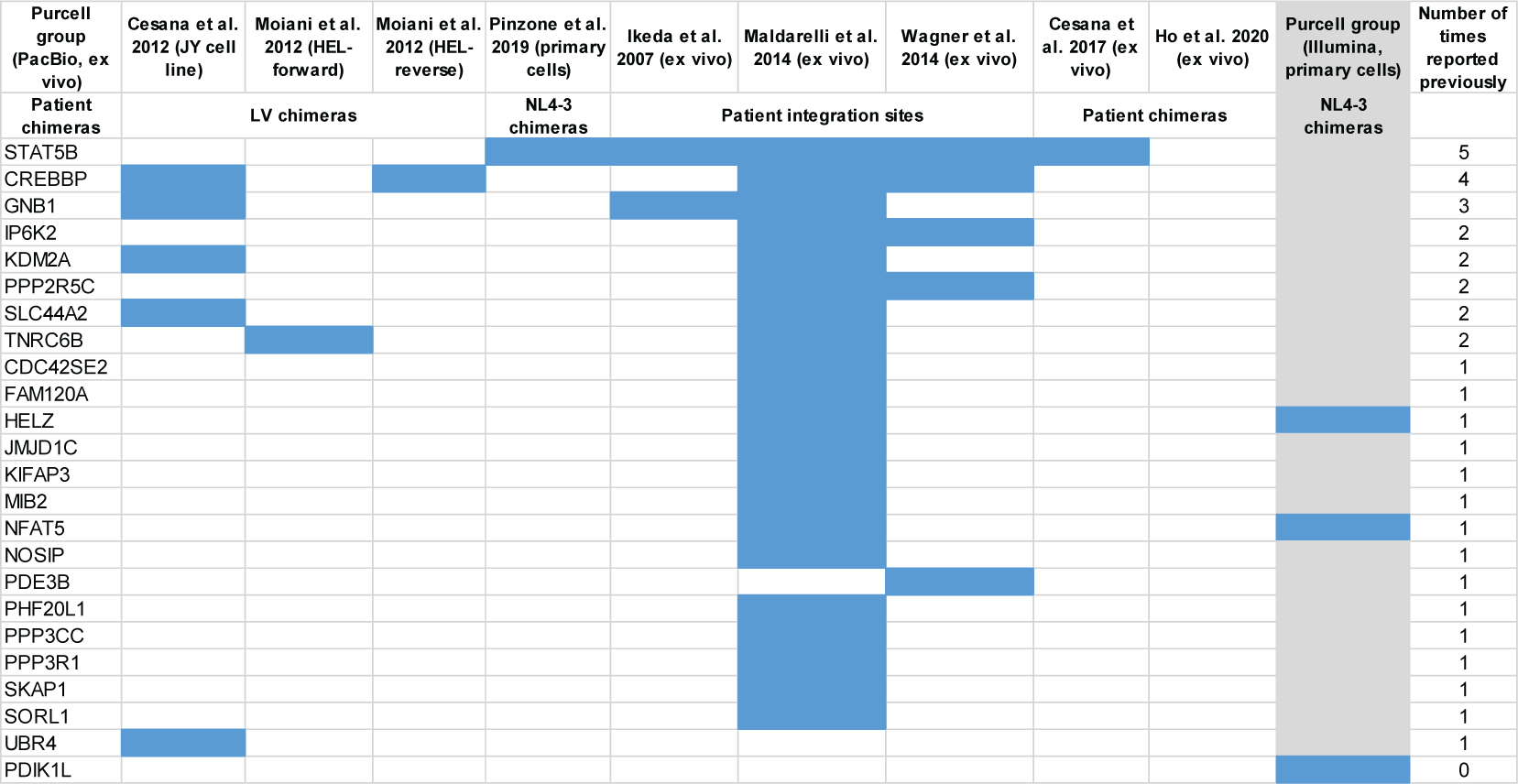

Supplement: Supplementary Figure 12 — Genes associated with cellular:HIV chimeras in the ex vivo dataset that have been reported as HIV-1 integration sites in previous studies. Genes that were also detected in our in vitro dataset are highlighted in the greyed out column on the right. The last column does not include the count in our in vitro dataset. [file Image_12.tif]

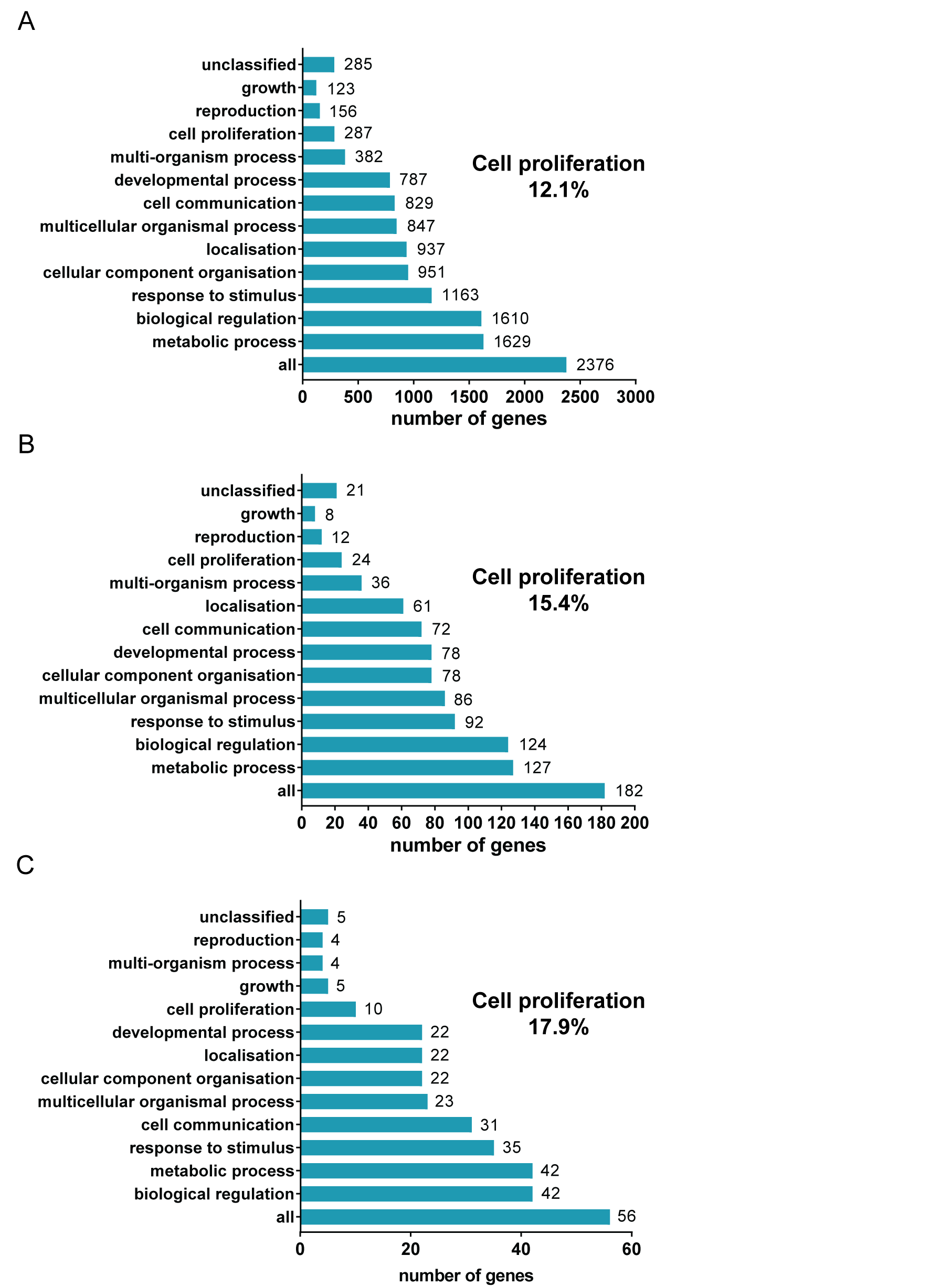

Supplement: Supplementary Figure 13 — Classification into major GO slim terms of genes found associated with chimeras. Genes associated with chimeras in the Illumina filtered (A), Illumina authentic (B) and PacBio (C) datasets were put through gene ontology analysis for broad biological process terms using the WEB-based GEne SeT AnaLysis Toolkit and shown as number of genes associated with each class. The proportion of genes associated with cell proliferation is shown in each case. [file Image_13.tif]
