# Supplementary material for: Detection of Chimeric Cellular: HIV mRNAs Generated Through Aberrant Splicing in HIV-1 Latently Infected Resting CD4+ T Cells
Source: Front Cell Infect Microbiol. 2022 Apr 28;12:855290. doi: 10.3389/fcimb.2022.855290 (PMC9096486; doi:10.3389/fcimb.2022.855290)
Supplement: Supplementary file 14 [file Presentation_1.pptx]

## Slide 1
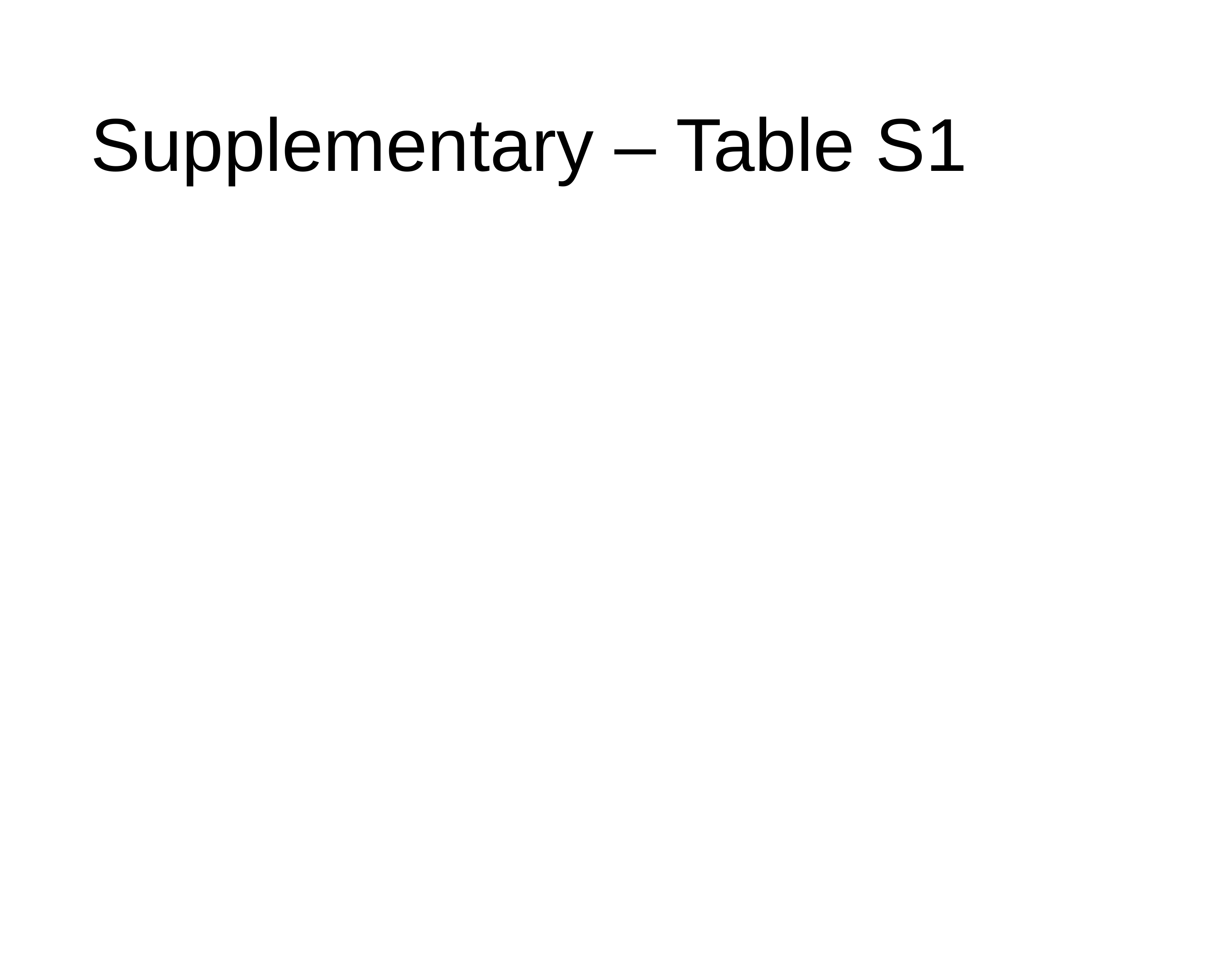

# Supplementary – Table S1

## Slide 2
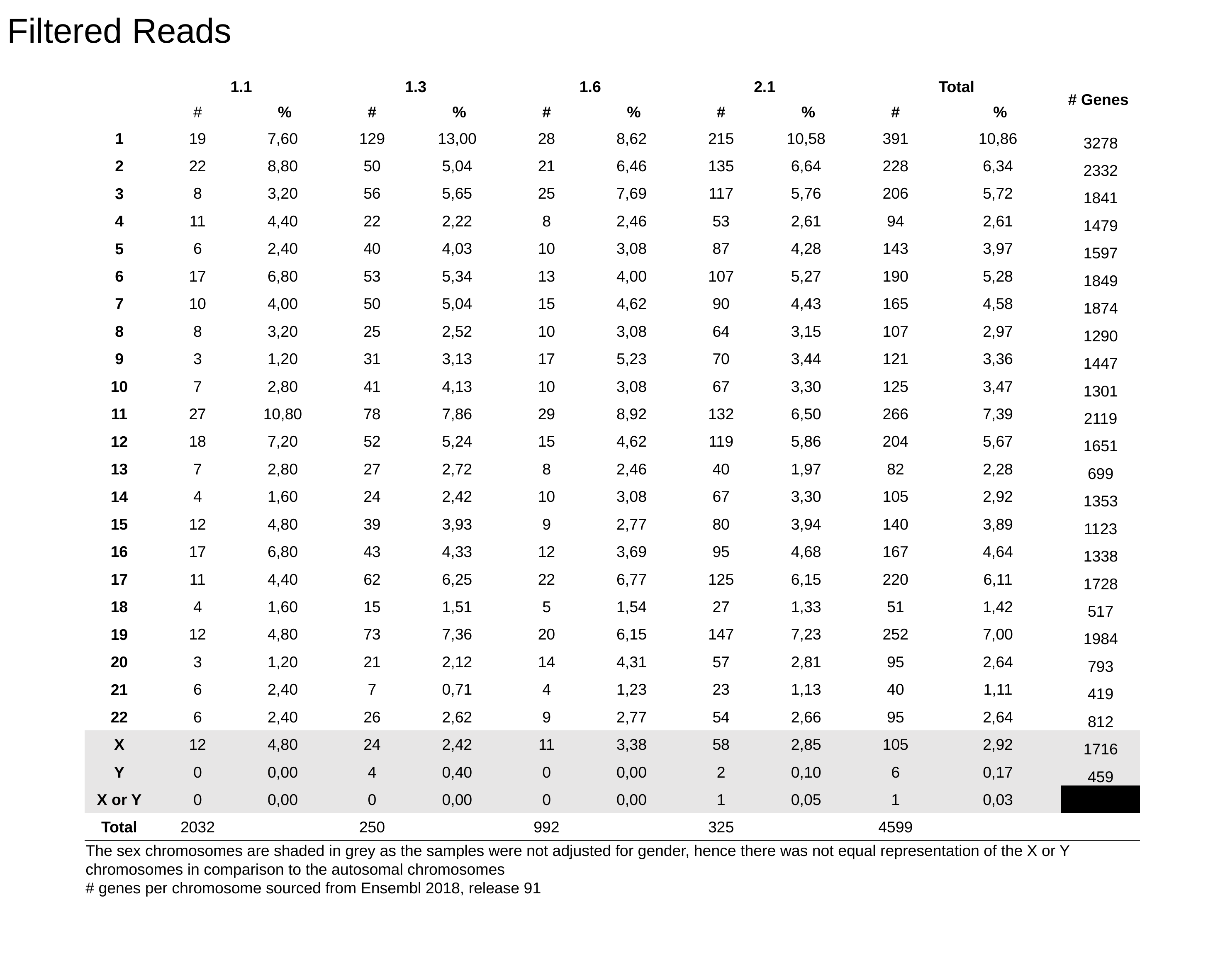

Filtered Reads
| | 1.1 | | 1.3 | | 1.6 | | 2.1 | | Total | | # Genes |
| --- | --- | --- | --- | --- | --- | --- | --- | --- | --- | --- | --- |
| | # | % | # | % | # | % | # | % | # | % | |
| 1 | 19 | 7,60 | 129 | 13,00 | 28 | 8,62 | 215 | 10,58 | 391 | 10,86 | 3278 |
| 2 | 22 | 8,80 | 50 | 5,04 | 21 | 6,46 | 135 | 6,64 | 228 | 6,34 | 2332 |
| 3 | 8 | 3,20 | 56 | 5,65 | 25 | 7,69 | 117 | 5,76 | 206 | 5,72 | 1841 |
| 4 | 11 | 4,40 | 22 | 2,22 | 8 | 2,46 | 53 | 2,61 | 94 | 2,61 | 1479 |
| 5 | 6 | 2,40 | 40 | 4,03 | 10 | 3,08 | 87 | 4,28 | 143 | 3,97 | 1597 |
| 6 | 17 | 6,80 | 53 | 5,34 | 13 | 4,00 | 107 | 5,27 | 190 | 5,28 | 1849 |
| 7 | 10 | 4,00 | 50 | 5,04 | 15 | 4,62 | 90 | 4,43 | 165 | 4,58 | 1874 |
| 8 | 8 | 3,20 | 25 | 2,52 | 10 | 3,08 | 64 | 3,15 | 107 | 2,97 | 1290 |
| 9 | 3 | 1,20 | 31 | 3,13 | 17 | 5,23 | 70 | 3,44 | 121 | 3,36 | 1447 |
| 10 | 7 | 2,80 | 41 | 4,13 | 10 | 3,08 | 67 | 3,30 | 125 | 3,47 | 1301 |
| 11 | 27 | 10,80 | 78 | 7,86 | 29 | 8,92 | 132 | 6,50 | 266 | 7,39 | 2119 |
| 12 | 18 | 7,20 | 52 | 5,24 | 15 | 4,62 | 119 | 5,86 | 204 | 5,67 | 1651 |
| 13 | 7 | 2,80 | 27 | 2,72 | 8 | 2,46 | 40 | 1,97 | 82 | 2,28 | 699 |
| 14 | 4 | 1,60 | 24 | 2,42 | 10 | 3,08 | 67 | 3,30 | 105 | 2,92 | 1353 |
| 15 | 12 | 4,80 | 39 | 3,93 | 9 | 2,77 | 80 | 3,94 | 140 | 3,89 | 1123 |
| 16 | 17 | 6,80 | 43 | 4,33 | 12 | 3,69 | 95 | 4,68 | 167 | 4,64 | 1338 |
| 17 | 11 | 4,40 | 62 | 6,25 | 22 | 6,77 | 125 | 6,15 | 220 | 6,11 | 1728 |
| 18 | 4 | 1,60 | 15 | 1,51 | 5 | 1,54 | 27 | 1,33 | 51 | 1,42 | 517 |
| 19 | 12 | 4,80 | 73 | 7,36 | 20 | 6,15 | 147 | 7,23 | 252 | 7,00 | 1984 |
| 20 | 3 | 1,20 | 21 | 2,12 | 14 | 4,31 | 57 | 2,81 | 95 | 2,64 | 793 |
| 21 | 6 | 2,40 | 7 | 0,71 | 4 | 1,23 | 23 | 1,13 | 40 | 1,11 | 419 |
| 22 | 6 | 2,40 | 26 | 2,62 | 9 | 2,77 | 54 | 2,66 | 95 | 2,64 | 812 |
| X | 12 | 4,80 | 24 | 2,42 | 11 | 3,38 | 58 | 2,85 | 105 | 2,92 | 1716 |
| Y | 0 | 0,00 | 4 | 0,40 | 0 | 0,00 | 2 | 0,10 | 6 | 0,17 | 459 |
| X or Y | 0 | 0,00 | 0 | 0,00 | 0 | 0,00 | 1 | 0,05 | 1 | 0,03 | |
| Total | 2032 | | 250 | | 992 | | 325 | | 4599 | | |
| The sex chromosomes are shaded in grey as the samples were not adjusted for gender, hence there was not equal representation of the X or Y chromosomes in comparison to the autosomal chromosomes # genes per chromosome sourced from Ensembl 2018, release 91 | | | | | | | | | | | |

## Slide 3
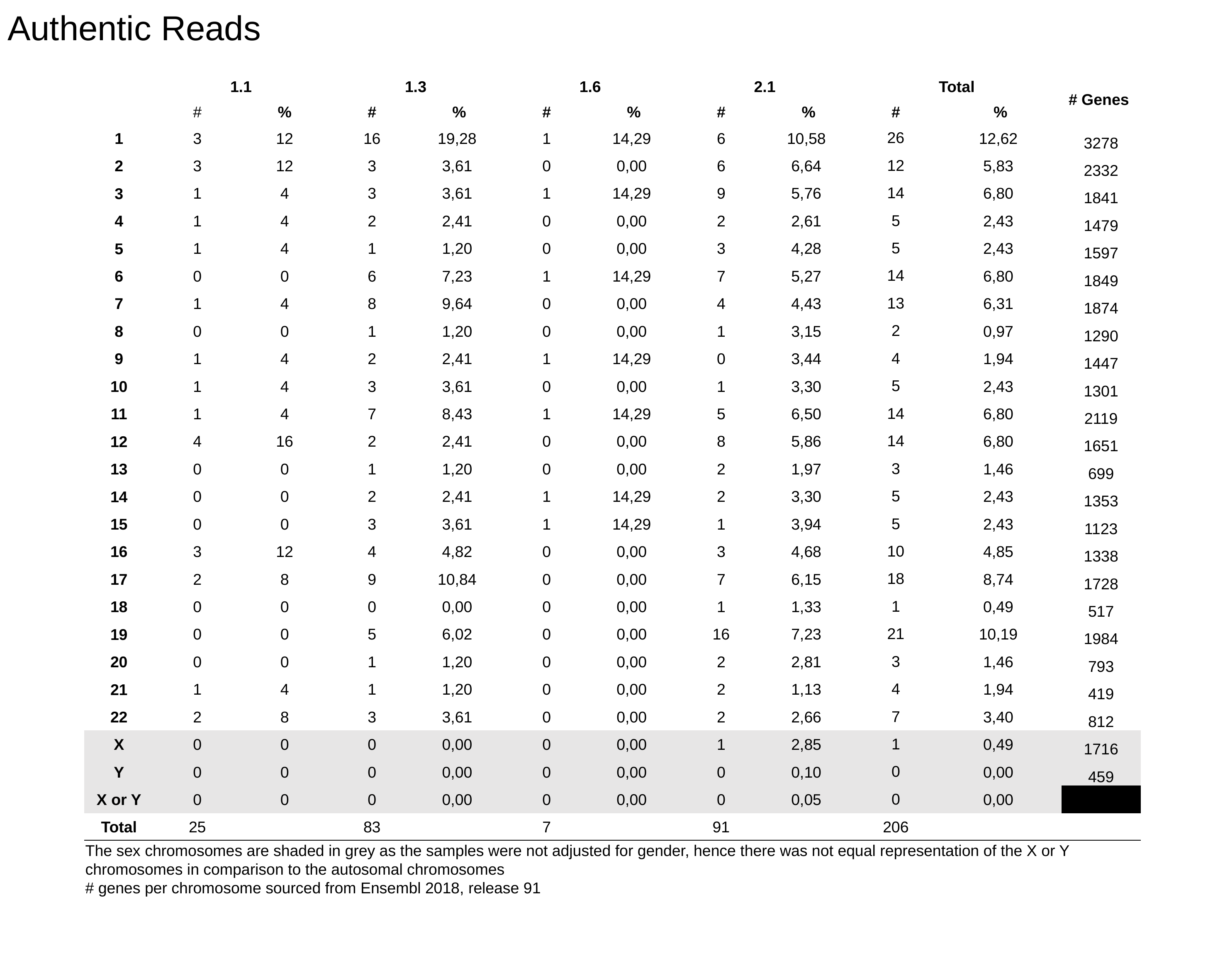

Authentic Reads
| | 1.1 | | 1.3 | | 1.6 | | 2.1 | | Total | | # Genes |
| --- | --- | --- | --- | --- | --- | --- | --- | --- | --- | --- | --- |
| | # | % | # | % | # | % | # | % | # | % | |
| 1 | 3 | 12 | 16 | 19,28 | 1 | 14,29 | 6 | 10,58 | 26 | 12,62 | 3278 |
| 2 | 3 | 12 | 3 | 3,61 | 0 | 0,00 | 6 | 6,64 | 12 | 5,83 | 2332 |
| 3 | 1 | 4 | 3 | 3,61 | 1 | 14,29 | 9 | 5,76 | 14 | 6,80 | 1841 |
| 4 | 1 | 4 | 2 | 2,41 | 0 | 0,00 | 2 | 2,61 | 5 | 2,43 | 1479 |
| 5 | 1 | 4 | 1 | 1,20 | 0 | 0,00 | 3 | 4,28 | 5 | 2,43 | 1597 |
| 6 | 0 | 0 | 6 | 7,23 | 1 | 14,29 | 7 | 5,27 | 14 | 6,80 | 1849 |
| 7 | 1 | 4 | 8 | 9,64 | 0 | 0,00 | 4 | 4,43 | 13 | 6,31 | 1874 |
| 8 | 0 | 0 | 1 | 1,20 | 0 | 0,00 | 1 | 3,15 | 2 | 0,97 | 1290 |
| 9 | 1 | 4 | 2 | 2,41 | 1 | 14,29 | 0 | 3,44 | 4 | 1,94 | 1447 |
| 10 | 1 | 4 | 3 | 3,61 | 0 | 0,00 | 1 | 3,30 | 5 | 2,43 | 1301 |
| 11 | 1 | 4 | 7 | 8,43 | 1 | 14,29 | 5 | 6,50 | 14 | 6,80 | 2119 |
| 12 | 4 | 16 | 2 | 2,41 | 0 | 0,00 | 8 | 5,86 | 14 | 6,80 | 1651 |
| 13 | 0 | 0 | 1 | 1,20 | 0 | 0,00 | 2 | 1,97 | 3 | 1,46 | 699 |
| 14 | 0 | 0 | 2 | 2,41 | 1 | 14,29 | 2 | 3,30 | 5 | 2,43 | 1353 |
| 15 | 0 | 0 | 3 | 3,61 | 1 | 14,29 | 1 | 3,94 | 5 | 2,43 | 1123 |
| 16 | 3 | 12 | 4 | 4,82 | 0 | 0,00 | 3 | 4,68 | 10 | 4,85 | 1338 |
| 17 | 2 | 8 | 9 | 10,84 | 0 | 0,00 | 7 | 6,15 | 18 | 8,74 | 1728 |
| 18 | 0 | 0 | 0 | 0,00 | 0 | 0,00 | 1 | 1,33 | 1 | 0,49 | 517 |
| 19 | 0 | 0 | 5 | 6,02 | 0 | 0,00 | 16 | 7,23 | 21 | 10,19 | 1984 |
| 20 | 0 | 0 | 1 | 1,20 | 0 | 0,00 | 2 | 2,81 | 3 | 1,46 | 793 |
| 21 | 1 | 4 | 1 | 1,20 | 0 | 0,00 | 2 | 1,13 | 4 | 1,94 | 419 |
| 22 | 2 | 8 | 3 | 3,61 | 0 | 0,00 | 2 | 2,66 | 7 | 3,40 | 812 |
| X | 0 | 0 | 0 | 0,00 | 0 | 0,00 | 1 | 2,85 | 1 | 0,49 | 1716 |
| Y | 0 | 0 | 0 | 0,00 | 0 | 0,00 | 0 | 0,10 | 0 | 0,00 | 459 |
| X or Y | 0 | 0 | 0 | 0,00 | 0 | 0,00 | 0 | 0,05 | 0 | 0,00 | |
| Total | 25 | | 83 | | 7 | | 91 | | 206 | | |
| The sex chromosomes are shaded in grey as the samples were not adjusted for gender, hence there was not equal representation of the X or Y chromosomes in comparison to the autosomal chromosomes # genes per chromosome sourced from Ensembl 2018, release 91 | | | | | | | | | | | |

## Slide 4
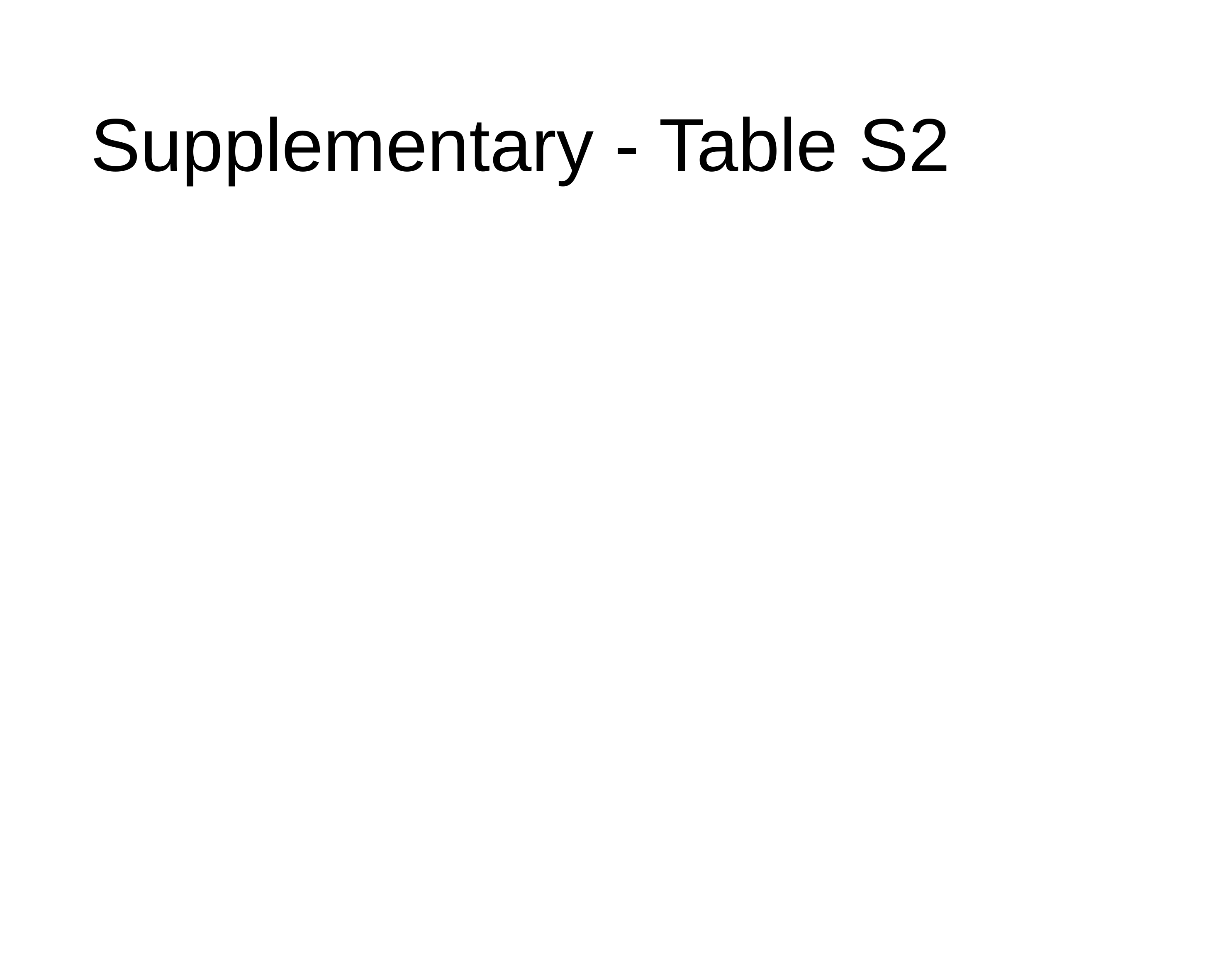

# Supplementary - Table S2

## Slide 5
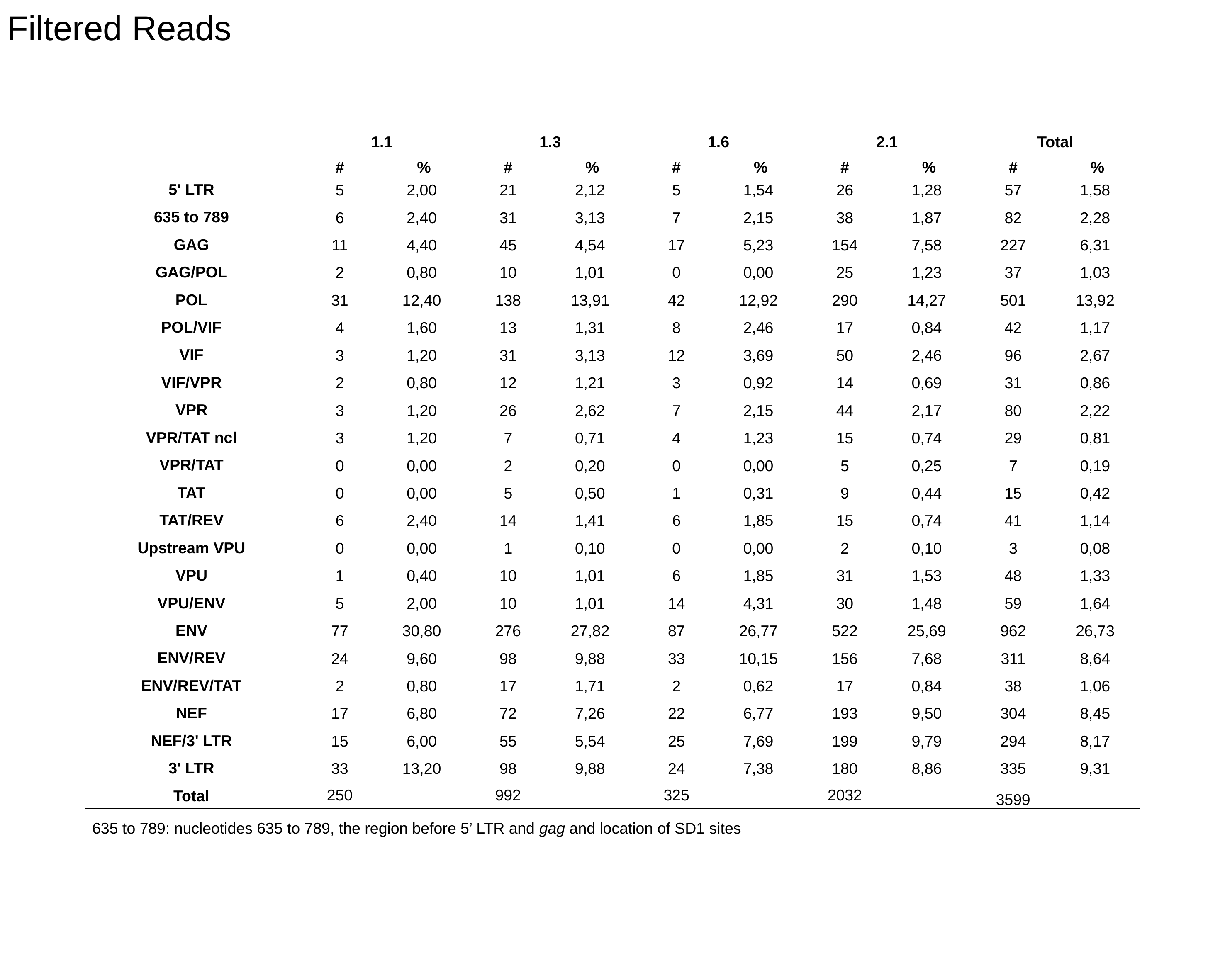

Filtered Reads
| | 1.1 | | 1.3 | | 1.6 | | 2.1 | | Total | |
| --- | --- | --- | --- | --- | --- | --- | --- | --- | --- | --- |
| | # | % | # | % | # | % | # | % | # | % |
| 5' LTR | 5 | 2,00 | 21 | 2,12 | 5 | 1,54 | 26 | 1,28 | 57 | 1,58 |
| 635 to 789 | 6 | 2,40 | 31 | 3,13 | 7 | 2,15 | 38 | 1,87 | 82 | 2,28 |
| GAG | 11 | 4,40 | 45 | 4,54 | 17 | 5,23 | 154 | 7,58 | 227 | 6,31 |
| GAG/POL | 2 | 0,80 | 10 | 1,01 | 0 | 0,00 | 25 | 1,23 | 37 | 1,03 |
| POL | 31 | 12,40 | 138 | 13,91 | 42 | 12,92 | 290 | 14,27 | 501 | 13,92 |
| POL/VIF | 4 | 1,60 | 13 | 1,31 | 8 | 2,46 | 17 | 0,84 | 42 | 1,17 |
| VIF | 3 | 1,20 | 31 | 3,13 | 12 | 3,69 | 50 | 2,46 | 96 | 2,67 |
| VIF/VPR | 2 | 0,80 | 12 | 1,21 | 3 | 0,92 | 14 | 0,69 | 31 | 0,86 |
| VPR | 3 | 1,20 | 26 | 2,62 | 7 | 2,15 | 44 | 2,17 | 80 | 2,22 |
| VPR/TAT ncl | 3 | 1,20 | 7 | 0,71 | 4 | 1,23 | 15 | 0,74 | 29 | 0,81 |
| VPR/TAT | 0 | 0,00 | 2 | 0,20 | 0 | 0,00 | 5 | 0,25 | 7 | 0,19 |
| TAT | 0 | 0,00 | 5 | 0,50 | 1 | 0,31 | 9 | 0,44 | 15 | 0,42 |
| TAT/REV | 6 | 2,40 | 14 | 1,41 | 6 | 1,85 | 15 | 0,74 | 41 | 1,14 |
| Upstream VPU | 0 | 0,00 | 1 | 0,10 | 0 | 0,00 | 2 | 0,10 | 3 | 0,08 |
| VPU | 1 | 0,40 | 10 | 1,01 | 6 | 1,85 | 31 | 1,53 | 48 | 1,33 |
| VPU/ENV | 5 | 2,00 | 10 | 1,01 | 14 | 4,31 | 30 | 1,48 | 59 | 1,64 |
| ENV | 77 | 30,80 | 276 | 27,82 | 87 | 26,77 | 522 | 25,69 | 962 | 26,73 |
| ENV/REV | 24 | 9,60 | 98 | 9,88 | 33 | 10,15 | 156 | 7,68 | 311 | 8,64 |
| ENV/REV/TAT | 2 | 0,80 | 17 | 1,71 | 2 | 0,62 | 17 | 0,84 | 38 | 1,06 |
| NEF | 17 | 6,80 | 72 | 7,26 | 22 | 6,77 | 193 | 9,50 | 304 | 8,45 |
| NEF/3' LTR | 15 | 6,00 | 55 | 5,54 | 25 | 7,69 | 199 | 9,79 | 294 | 8,17 |
| 3' LTR | 33 | 13,20 | 98 | 9,88 | 24 | 7,38 | 180 | 8,86 | 335 | 9,31 |
| Total | 250 | | 992 | | 325 | | 2032 | | 3599 | |
| 635 to 789: nucleotides 635 to 789, the region before 5’ LTR and gag and location of SD1 sites | | | | | | | | | | |

## Slide 6
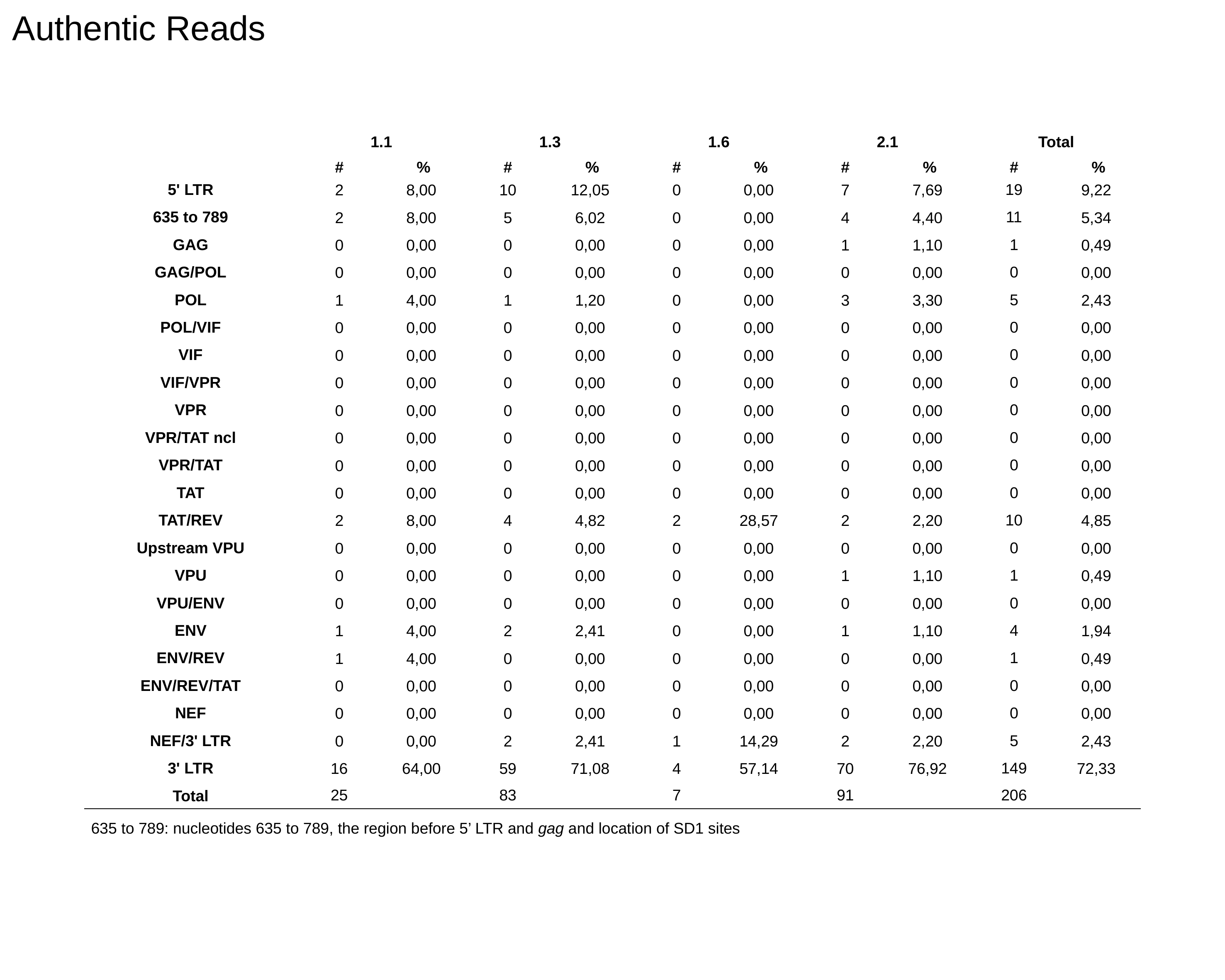

Authentic Reads
| | 1.1 | | 1.3 | | 1.6 | | 2.1 | | Total | |
| --- | --- | --- | --- | --- | --- | --- | --- | --- | --- | --- |
| | # | % | # | % | # | % | # | % | # | % |
| 5' LTR | 2 | 8,00 | 10 | 12,05 | 0 | 0,00 | 7 | 7,69 | 19 | 9,22 |
| 635 to 789 | 2 | 8,00 | 5 | 6,02 | 0 | 0,00 | 4 | 4,40 | 11 | 5,34 |
| GAG | 0 | 0,00 | 0 | 0,00 | 0 | 0,00 | 1 | 1,10 | 1 | 0,49 |
| GAG/POL | 0 | 0,00 | 0 | 0,00 | 0 | 0,00 | 0 | 0,00 | 0 | 0,00 |
| POL | 1 | 4,00 | 1 | 1,20 | 0 | 0,00 | 3 | 3,30 | 5 | 2,43 |
| POL/VIF | 0 | 0,00 | 0 | 0,00 | 0 | 0,00 | 0 | 0,00 | 0 | 0,00 |
| VIF | 0 | 0,00 | 0 | 0,00 | 0 | 0,00 | 0 | 0,00 | 0 | 0,00 |
| VIF/VPR | 0 | 0,00 | 0 | 0,00 | 0 | 0,00 | 0 | 0,00 | 0 | 0,00 |
| VPR | 0 | 0,00 | 0 | 0,00 | 0 | 0,00 | 0 | 0,00 | 0 | 0,00 |
| VPR/TAT ncl | 0 | 0,00 | 0 | 0,00 | 0 | 0,00 | 0 | 0,00 | 0 | 0,00 |
| VPR/TAT | 0 | 0,00 | 0 | 0,00 | 0 | 0,00 | 0 | 0,00 | 0 | 0,00 |
| TAT | 0 | 0,00 | 0 | 0,00 | 0 | 0,00 | 0 | 0,00 | 0 | 0,00 |
| TAT/REV | 2 | 8,00 | 4 | 4,82 | 2 | 28,57 | 2 | 2,20 | 10 | 4,85 |
| Upstream VPU | 0 | 0,00 | 0 | 0,00 | 0 | 0,00 | 0 | 0,00 | 0 | 0,00 |
| VPU | 0 | 0,00 | 0 | 0,00 | 0 | 0,00 | 1 | 1,10 | 1 | 0,49 |
| VPU/ENV | 0 | 0,00 | 0 | 0,00 | 0 | 0,00 | 0 | 0,00 | 0 | 0,00 |
| ENV | 1 | 4,00 | 2 | 2,41 | 0 | 0,00 | 1 | 1,10 | 4 | 1,94 |
| ENV/REV | 1 | 4,00 | 0 | 0,00 | 0 | 0,00 | 0 | 0,00 | 1 | 0,49 |
| ENV/REV/TAT | 0 | 0,00 | 0 | 0,00 | 0 | 0,00 | 0 | 0,00 | 0 | 0,00 |
| NEF | 0 | 0,00 | 0 | 0,00 | 0 | 0,00 | 0 | 0,00 | 0 | 0,00 |
| NEF/3' LTR | 0 | 0,00 | 2 | 2,41 | 1 | 14,29 | 2 | 2,20 | 5 | 2,43 |
| 3' LTR | 16 | 64,00 | 59 | 71,08 | 4 | 57,14 | 70 | 76,92 | 149 | 72,33 |
| Total | 25 | | 83 | | 7 | | 91 | | 206 | |
| 635 to 789: nucleotides 635 to 789, the region before 5’ LTR and gag and location of SD1 sites | | | | | | | | | | |

## Slide 7
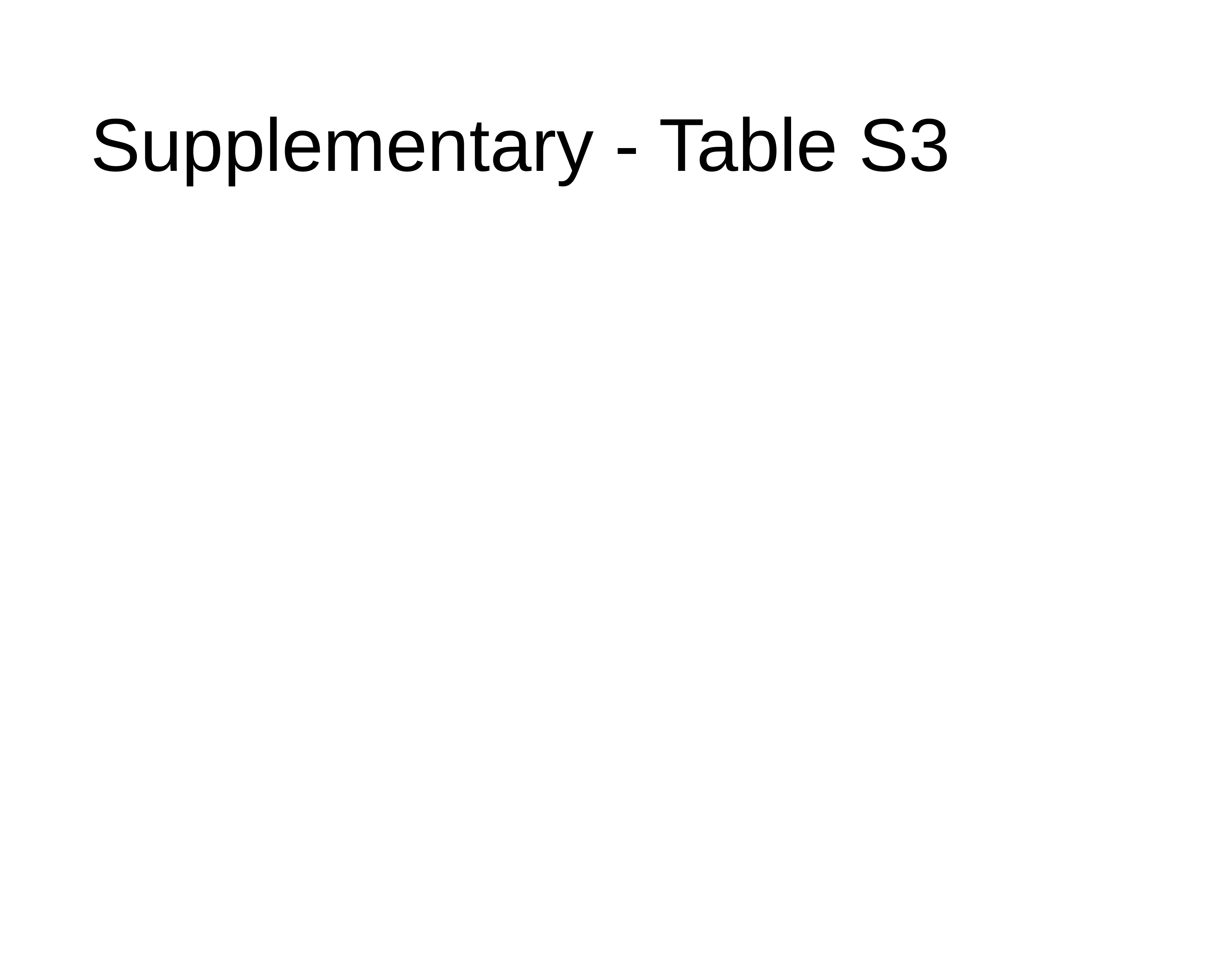

# Supplementary - Table S3

## Slide 8
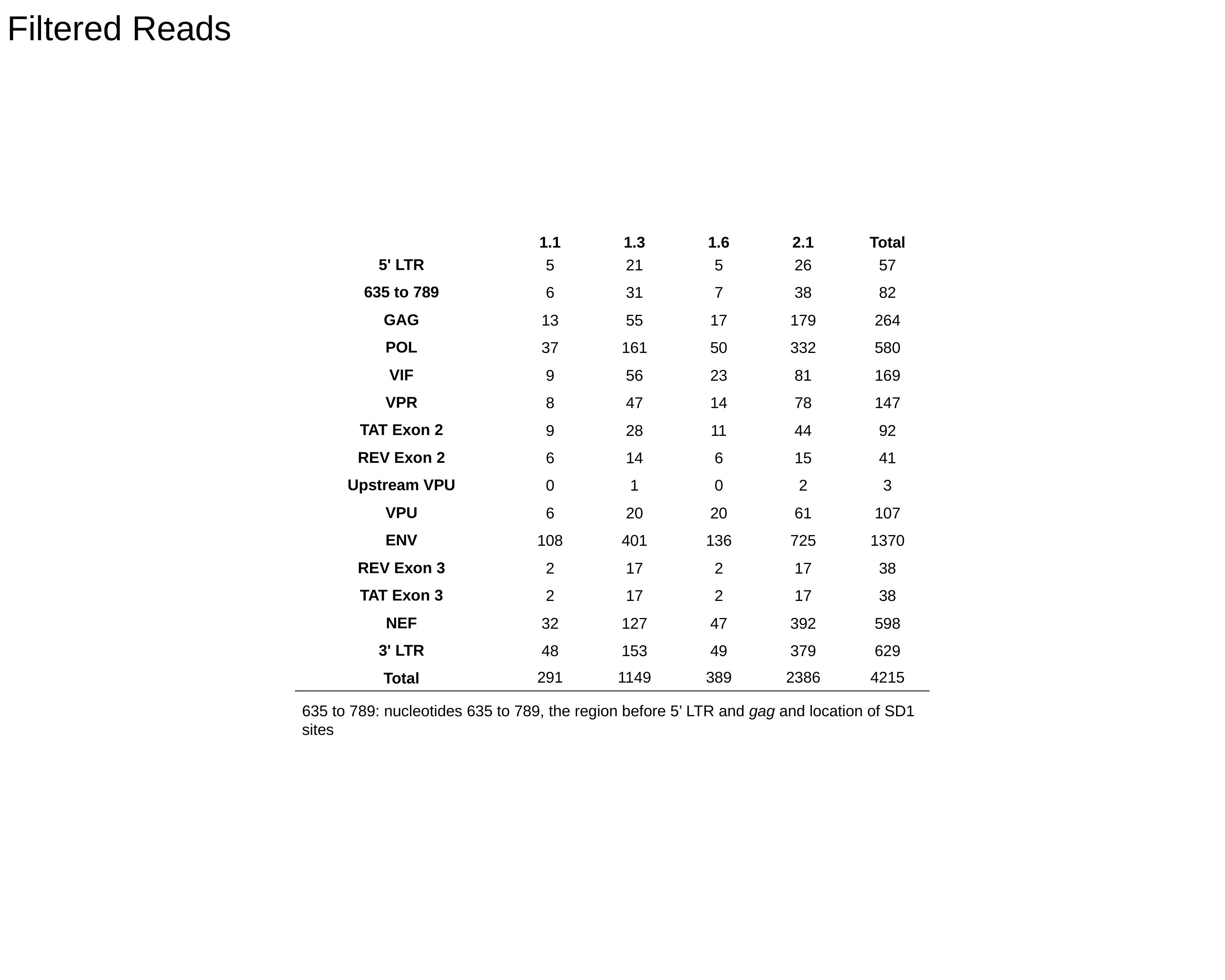

Filtered Reads
| | 1.1 | 1.3 | 1.6 | 2.1 | Total |
| --- | --- | --- | --- | --- | --- |
| 5' LTR | 5 | 21 | 5 | 26 | 57 |
| 635 to 789 | 6 | 31 | 7 | 38 | 82 |
| GAG | 13 | 55 | 17 | 179 | 264 |
| POL | 37 | 161 | 50 | 332 | 580 |
| VIF | 9 | 56 | 23 | 81 | 169 |
| VPR | 8 | 47 | 14 | 78 | 147 |
| TAT Exon 2 | 9 | 28 | 11 | 44 | 92 |
| REV Exon 2 | 6 | 14 | 6 | 15 | 41 |
| Upstream VPU | 0 | 1 | 0 | 2 | 3 |
| VPU | 6 | 20 | 20 | 61 | 107 |
| ENV | 108 | 401 | 136 | 725 | 1370 |
| REV Exon 3 | 2 | 17 | 2 | 17 | 38 |
| TAT Exon 3 | 2 | 17 | 2 | 17 | 38 |
| NEF | 32 | 127 | 47 | 392 | 598 |
| 3' LTR | 48 | 153 | 49 | 379 | 629 |
| Total | 291 | 1149 | 389 | 2386 | 4215 |
| 635 to 789: nucleotides 635 to 789, the region before 5’ LTR and gag and location of SD1 sites | | | | | |

## Slide 9
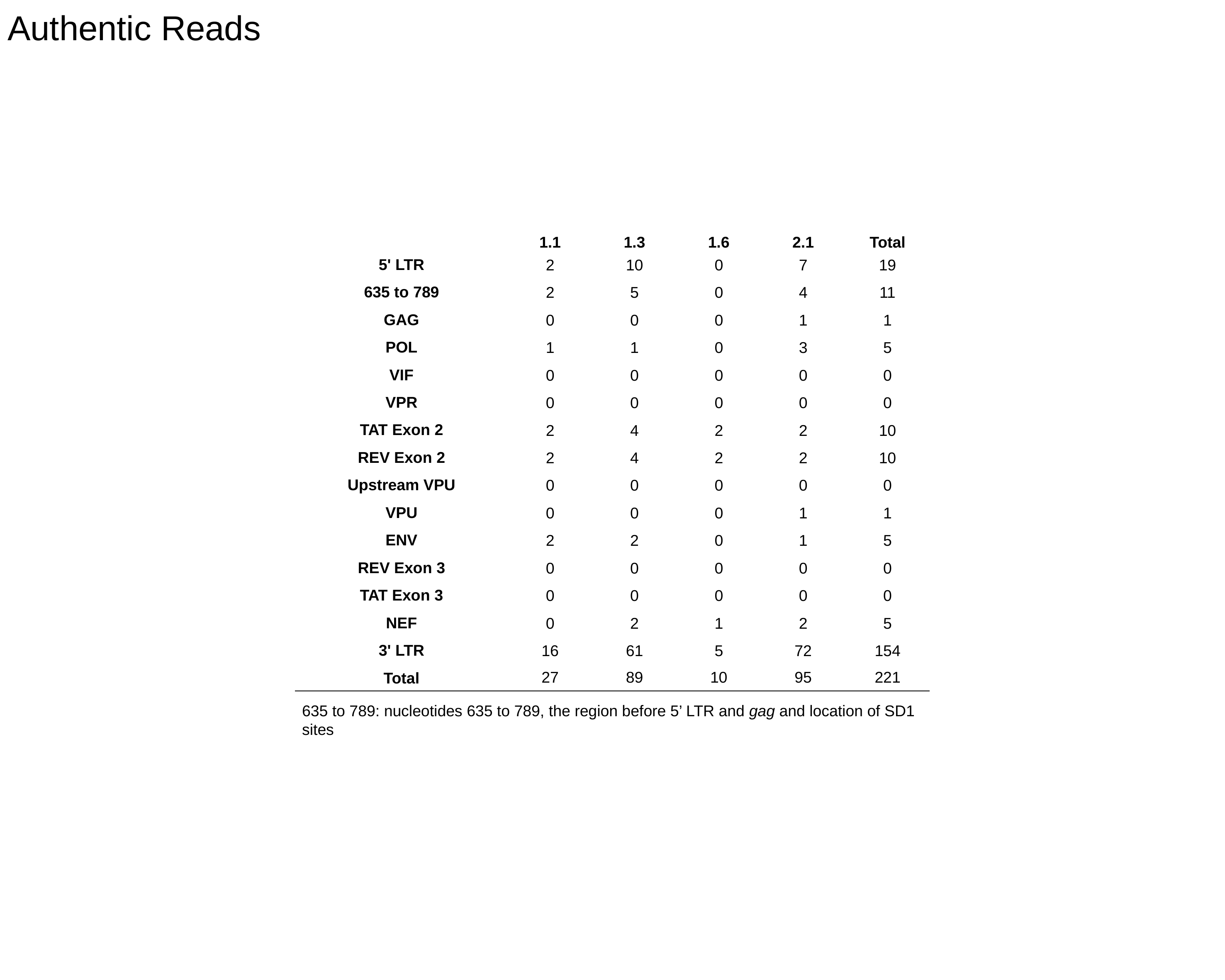

Authentic Reads
| | 1.1 | 1.3 | 1.6 | 2.1 | Total |
| --- | --- | --- | --- | --- | --- |
| 5' LTR | 2 | 10 | 0 | 7 | 19 |
| 635 to 789 | 2 | 5 | 0 | 4 | 11 |
| GAG | 0 | 0 | 0 | 1 | 1 |
| POL | 1 | 1 | 0 | 3 | 5 |
| VIF | 0 | 0 | 0 | 0 | 0 |
| VPR | 0 | 0 | 0 | 0 | 0 |
| TAT Exon 2 | 2 | 4 | 2 | 2 | 10 |
| REV Exon 2 | 2 | 4 | 2 | 2 | 10 |
| Upstream VPU | 0 | 0 | 0 | 0 | 0 |
| VPU | 0 | 0 | 0 | 1 | 1 |
| ENV | 2 | 2 | 0 | 1 | 5 |
| REV Exon 3 | 0 | 0 | 0 | 0 | 0 |
| TAT Exon 3 | 0 | 0 | 0 | 0 | 0 |
| NEF | 0 | 2 | 1 | 2 | 5 |
| 3' LTR | 16 | 61 | 5 | 72 | 154 |
| Total | 27 | 89 | 10 | 95 | 221 |
| 635 to 789: nucleotides 635 to 789, the region before 5’ LTR and gag and location of SD1 sites | | | | | |
